# Supplementary material for: Neoadjuvant or concurrent atezolizumab with chemoradiation for locally advanced cervical cancer: a randomized phase I trial
Source: Nat Commun. 2025 Jan 9;16:553. doi: 10.1038/s41467-024-55200-2 (PMC11718273; doi:10.1038/s41467-024-55200-2)
Supplement: Supplementary file 1 — Supplementary Information [file 41467_2024_55200_MOESM1_ESM.pdf]

Supplementary Figure 1

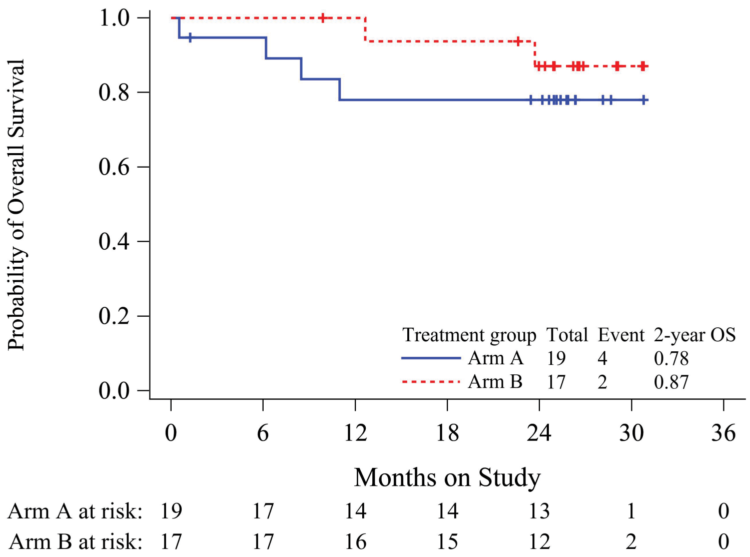

Supplementary Figure 1: Kaplan-Meier curves for overall survival by treatment.

Supplementary Figure 2

A.

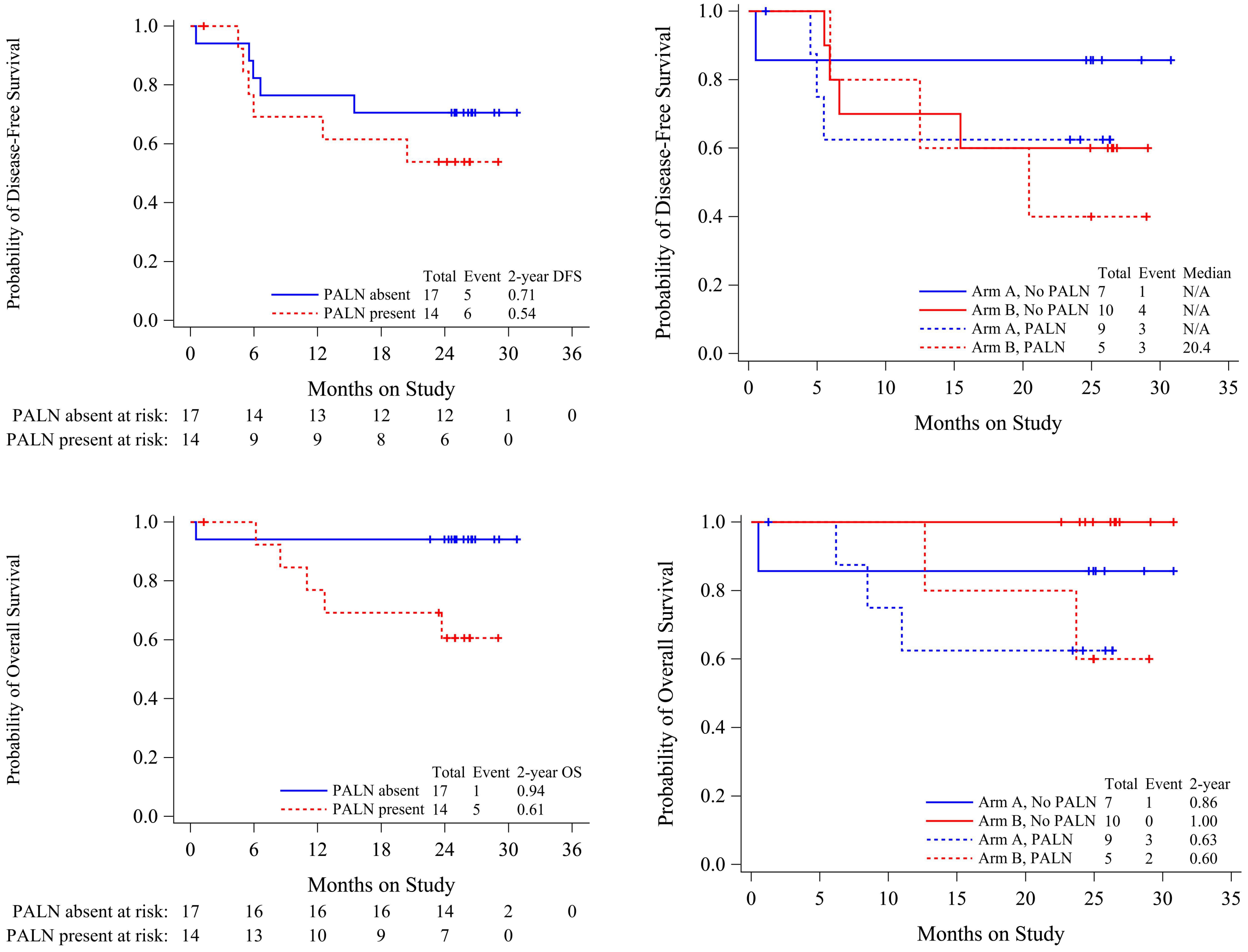

Supplementary Figure 2: Clinical outcomes as a function of PALN status. A. Kaplan-Meier curves of Disease-Free Survival (DFS) by PALN and Treatment. Left: DFS of overall cohort. Right: DFS by treatment arm. B. Kaplan-Meier curves of Overall Survival by PALN and Treatment. Left: overall survival of the overall cohort. Right: overall survival by treatment arm.

Supplementary Figure 3

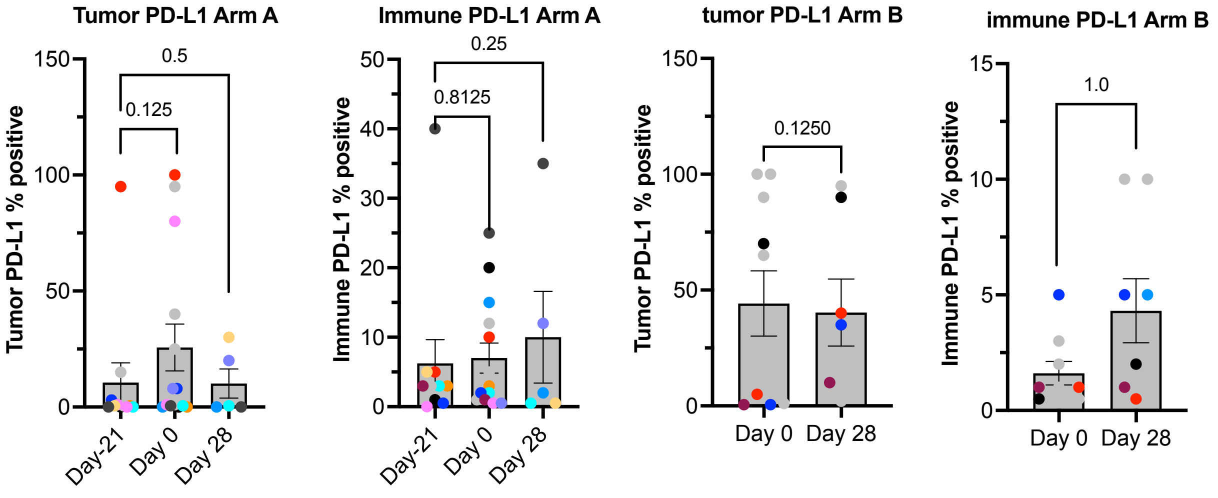

Supplementary Figure 3: Evolution of PD-L1 positivity over treatment course. PD-L1 positivity in tumor cells and immune cells as a function of treatment arm and time is shown (Arm A, day -21 n=11, day 0 n=14, day 28 n=5; Arm B, day 0 n=10, day 28 n=7). Matched samples are indicated by colored symbols; unmatched samples are in grey. Individual comparisons were performed using Individual comparisons were performed using Wilcoxon Signed Rank test. Source data are provided as a Source Data file. Each data point represents an individual patient with an individual measurement for the specified time point. There were no adjustments for multiple comparisons.

Supplementary Figure 4

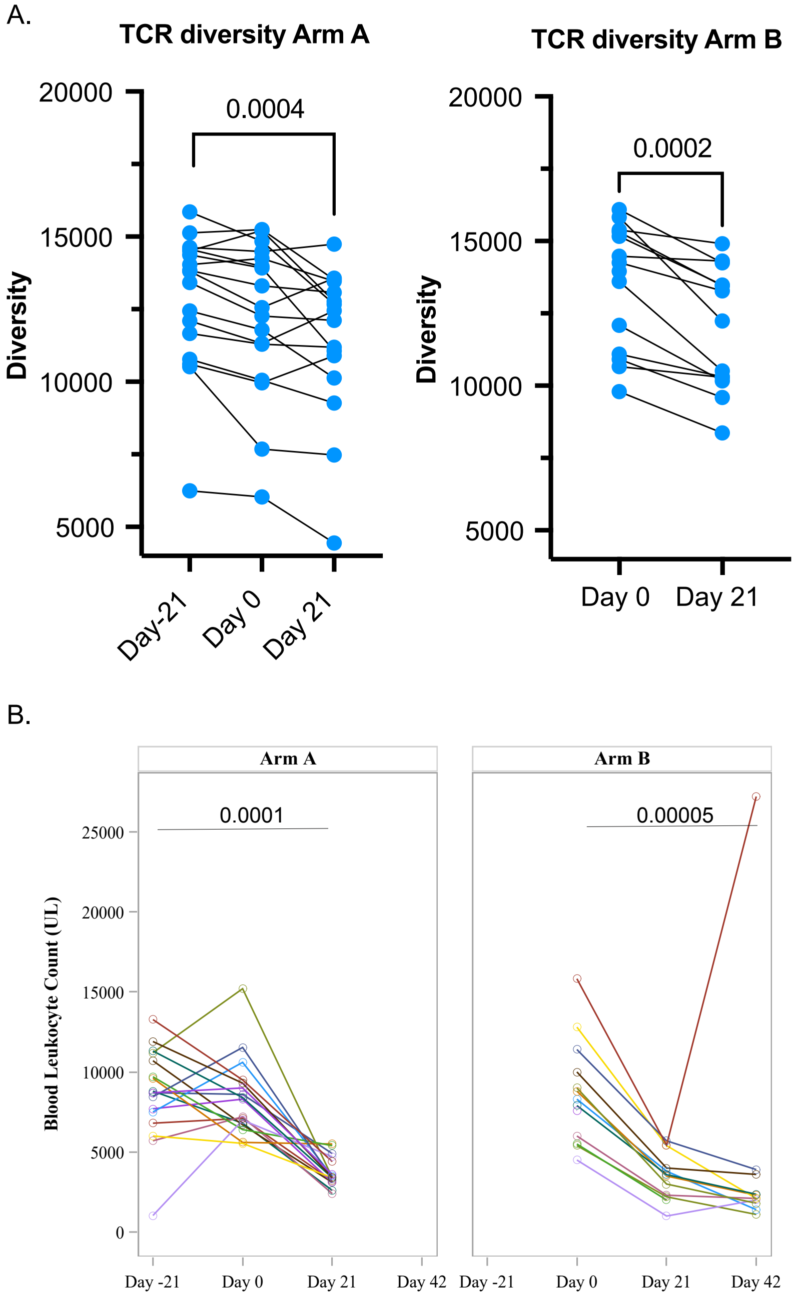

Supplementary Figure 4: Change in T cell diversity and leukocyte counts over the course of therapy. A. Change in TCR diversity by arm (Arm A, n=17; Arm B, n=13). B. Change in total leukocyte counts by arm (Arm A, n=17, Arm B, n=13). Comparisons were performed using Wilcoxon signed rank test at 10% significance level. Source data are provided as a Source Data file. Each data point represents an individual patient with an individual measurement for the specified time point. There were no adjustments for multiple comparisons.

Supplementary Figure 5

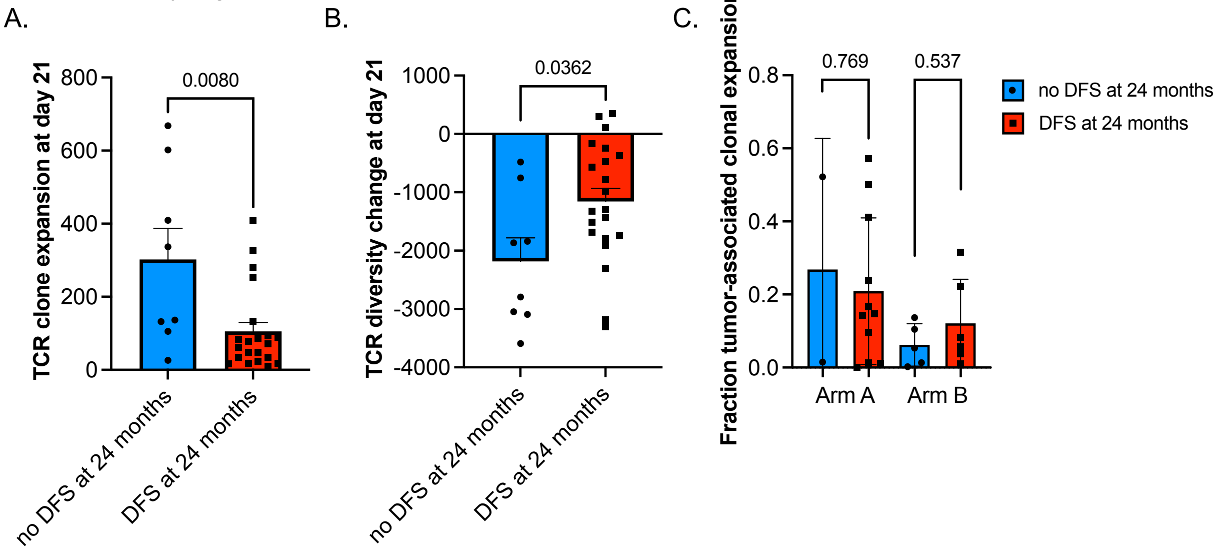

Supplementary Figure 5: Association of T cell receptor parameters with Disease Free Survival. A. Association of total clonal expansion at day 21 with 2-year DFS. B. Association of TCR diversity change at day 21 with 2-year DFS (no DFS at 24 months: n=8, DFS at 24 months: n=21). C. Association of tumor-associated clonal expansion at day 21 with 2-year DFS by treatment arm (no DFS at 24 months Arm A: n=3, Arm B: n=5; DFS at 24 months Arm A: n=13, Arm B: n=8). Spearman rank correlation coefficient tests were used for all analyses at 10% significance level. There were no adjustments for multiple comparisons.

Supplementary Table 1: Highest Grade Adverse Events Related to Protocol Therapy

	Arm A (n=19)					Arm B (n=17)				
	No. and (%) of Patients by Grade					No. and (%) of Patients by Grade				
System Organ Class	1	2	3	4	5	1	2	3	4	5
Overall Highest Grade	4	9	3	0	0	2	2	7	3	0
	(21.1)	(47.4)	(15.8)	(0.0)	(0.0)	(11.8)	(11.8)	(41.2)	(17.6)	(0.0)
Blood and lymphatic system disorders	0	3	0	0	0	0	3	3	1	0
	(0.0)	(15.8)	(0.0)	(0.0)	(0.0)	(0.0)	(17.6)	(17.6)	(5.9)	(0.0)
Ear and labyrinth disorders	3	1	0	0	0	2	1	0	0	0
	(15.8)	(5.3)	(0.0)	(0.0)	(0.0)	(11.8)	(5.9)	(0.0)	(0.0)	(0.0)
Endocrine disorders	1	0	0	0	0	0	0	0	0	0
	(5.3)	(0.0)	(0.0)	(0.0)	(0.0)	(0.0)	(0.0)	(0.0)	(0.0)	(0.0)
Eye disorders	1	0	0	0	0	0	0	0	0	0
	(5.3)	(0.0)	(0.0)	(0.0)	(0.0)	(0.0)	(0.0)	(0.0)	(0.0)	(0.0)
Gastrointestinal disorders	3	6	1	0	0	4	4	3	1	0
	(15.8)	(31.6)	(5.3)	(0.0)	(0.0)	(23.5)	(23.5)	(17.6)	(5.9)	(0.0)
General disorders and administration site conditions	7	1	0	0	0	6	3	0	0	0
	(36.8)	(5.3)	(0.0)	(0.0)	(0.0)	(35.3)	(17.6)	(0.0)	(0.0)	(0.0)
Infections and infestations	0	0	1	0	0	0	1	1	0	0
	(0.0)	(0.0)	(5.3)	(0.0)	(0.0)	(0.0)	(5.9)	(5.9)	(0.0)	(0.0)
Injury, poisoning and procedural complications	0	2	0	0	0	0	1	0	0	0
	(0.0)	(10.5)	(0.0)	(0.0)	(0.0)	(0.0)	(5.9)	(0.0)	(0.0)	(0.0)
Investigations	3	3	1	0	0	0	5	3	1	0
	(15.8)	(15.8)	(5.3)	(0.0)	(0.0)	(0.0)	(29.4)	(17.6)	(5.9)	(0.0)
Metabolism and nutrition disorders	6	1	0	0	0	3	1	1	1	0
	(31.6)	(5.3)	(0.0)	(0.0)	(0.0)	(17.6)	(5.9)	(5.9)	(5.9)	(0.0)
Musculoskeletal and connective tissue disorders	2	0	0	0	0	0	0	0	0	0
	(10.5)	(0.0)	(0.0)	(0.0)	(0.0)	(0.0)	(0.0)	(0.0)	(0.0)	(0.0)
Nervous system disorders	5	0	0	0	0	2	1	0	0	0
	(26.3)	(0.0)	(0.0)	(0.0)	(0.0)	(11.8)	(5.9)	(0.0)	(0.0)	(0.0)
Psychiatric disorders	2	0	0	0	0	1	1	0	0	0
	(10.5)	(0.0)	(0.0)	(0.0)	(0.0)	(5.9)	(5.9)	(0.0)	(0.0)	(0.0)
Renal and urinary disorders	3	1	1	0	0	1	0	0	0	0
	(15.8)	(5.3)	(5.3)	(0.0)	(0.0)	(5.9)	(0.0)	(0.0)	(0.0)	(0.0)

Reproductive system and breast disorders	1	0	0	0	0	0	0	0	0	0
	(5.3)	(0.0)	(0.0)	(0.0)	(0.0)	(0.0)	(0.0)	(0.0)	(0.0)	(0.0)
Respiratory, thoracic and mediastinal disorders	1	0	0	0	0	3	0	0	0	0
	(5.3)	(0.0)	(0.0)	(0.0)	(0.0)	(17.6)	(0.0)	(0.0)	(0.0)	(0.0)
Skin and subcutaneous tissue disorders	2	0	0	0	0	2	0	0	0	0
	(10.5)	(0.0)	(0.0)	(0.0)	(0.0)	(11.8)	(0.0)	(0.0)	(0.0)	(0.0)

Supplementary Table 1: Highest Grade Adverse Events Related to Protocol Therapy

Supplementary Table 2: Highest Grade Adverse Events Regardless of Attribution to Protocol Therapy

	Arm A (n=19)					Arm B (n=17)				
	No. and (%) of Patients by Grade					No. and (%) of Patients by Grade				
System Organ Class/Term	1	2	3	4	5	1	2	3	4	5
Overall Highest Grade	2	7	9	0	1	1	2	10	4	0
	(10.5)	(36.8)	(47.4)	(0.0)	(5.3)	(5.9)	(11.8)	(58.8)	(23.5)	(0.0)
Blood and Lymphatic System Disorders	1	5	4	0	0	0	4	5	1	0
	(5.3)	(26.3)	(21.1)	(0.0)	(0.0)	(0.0)	(23.5)	(29.4)	(5.9)	(0.0)
Anemia	1	5	4	0	0	0	5	4	0	0
	(5.3)	(26.3)	(21.1)	(0.0)	(0.0)	(0.0)	(29.4)	(23.5)	(0.0)	(0.0)
Febrile neutropenia	0	0	0	0	0	0	0	1	1	0
	(0.0)	(0.0)	(0.0)	(0.0)	(0.0)	(0.0)	(0.0)	(5.9)	(5.9)	(0.0)
Blood and lymphatic system disorders - other	0	0	0	0	0	0	0	1	0	0
	(0.0)	(0.0)	(0.0)	(0.0)	(0.0)	(0.0)	(0.0)	(5.9)	(0.0)	(0.0)
Cardiac Disorders	0	0	0	0	1	0	1	0	0	0
	(0.0)	(0.0)	(0.0)	(0.0)	(5.3)	(0.0)	(5.9)	(0.0)	(0.0)	(0.0)
Myocardial infarction	0	0	0	0	1	0	0	0	0	0
	(0.0)	(0.0)	(0.0)	(0.0)	(5.3)	(0.0)	(0.0)	(0.0)	(0.0)	(0.0)
Gastrointestinal Disorders	4	12	2	0	0	6	5	3	1	0
	(21.1)	(63.2)	(10.5)	(0.0)	(0.0)	(35.3)	(29.4)	(17.6)	(5.9)	(0.0)
Diarrhea	6	5	2	0	0	4	3	3	0	0
	(31.6)	(26.3)	(10.5)	(0.0)	(0.0)	(23.5)	(17.6)	(17.6)	(0.0)	(0.0)
Mucositis oral	0	1	0	0	0	0	0	0	1	0
	(0.0)	(5.3)	(0.0)	(0.0)	(0.0)	(0.0)	(0.0)	(0.0)	(5.9)	(0.0)
Nausea	8	8	0	0	0	6	2	2	0	0
	(42.1)	(42.1)	(0.0)	(0.0)	(0.0)	(35.3)	(11.8)	(11.8)	(0.0)	(0.0)
Small intestinal obstruction	0	0	0	0	0	0	0	1	0	0
	(0.0)	(0.0)	(0.0)	(0.0)	(0.0)	(0.0)	(0.0)	(5.9)	(0.0)	(0.0)
Vomiting	4	3	0	0	0	5	1	2	0	0
	(21.1)	(15.8)	(0.0)	(0.0)	(0.0)	(29.4)	(5.9)	(11.8)	(0.0)	(0.0)
General Disorders and Administration Site Conditions	9	4	1	0	0	4	10	0	0	0
	(47.4)	(21.1)	(5.3)	(0.0)	(0.0)	(23.5)	(58.8)	(0.0)	(0.0)	(0.0)
Flu like symptoms	0	0	1	0	0	0	0	0	0	0
	(0.0)	(0.0)	(5.3)	(0.0)	(0.0)	(0.0)	(0.0)	(0.0)	(0.0)	(0.0)
Infections and Infestations	0	3	3	0	0	1	1	2	0	0

	(0.0)	(15.8)	(15.8)	(0.0)	(0.0)	(5.9)	(5.9)	(11.8)	(0.0)	(0.0)
Urinary tract infection	0	2	3	0	0	0	0	2	0	0
	(0.0)	(10.5)	(15.8)	(0.0)	(0.0)	(0.0)	(0.0)	(11.8)	(0.0)	(0.0)
Investigations	4	3	2	0	0	0	4	5	2	0
	(21.1)	(15.8)	(10.5)	(0.0)	(0.0)	(0.0)	(23.5)	(29.4)	(11.8)	(0.0)
Lymphocyte count decreased	0	0	0	0	0	0	0	2	1	0
	(0.0)	(0.0)	(0.0)	(0.0)	(0.0)	(0.0)	(0.0)	(11.8)	(5.9)	(0.0)
Neutrophil count decreased	1	2	1	0	0	0	1	5	0	0
	(5.3)	(10.5)	(5.3)	(0.0)	(0.0)	(0.0)	(5.9)	(29.4)	(0.0)	(0.0)
Platelet count decreased	4	0	0	0	0	6	0	0	1	0
	(21.1)	(0.0)	(0.0)	(0.0)	(0.0)	(35.3)	(0.0)	(0.0)	(5.9)	(0.0)
White blood cell decreased	2	1	1	0	0	1	3	2	0	0
	(10.5)	(5.3)	(5.3)	(0.0)	(0.0)	(5.9)	(17.6)	(11.8)	(0.0)	(0.0)
Metabolism and Nutrition Disorders	9	3	0	0	0	4	4	3	1	0
	(47.4)	(15.8)	(0.0)	(0.0)	(0.0)	(23.5)	(23.5)	(17.6)	(5.9)	(0.0)
Hypocalcemia	2	0	0	0	0	3	2	1	0	0
	(10.5)	(0.0)	(0.0)	(0.0)	(0.0)	(17.6)	(11.8)	(5.9)	(0.0)	(0.0)
Hypokalemia	5	1	0	0	0	4	1	2	0	0
	(26.3)	(5.3)	(0.0)	(0.0)	(0.0)	(23.5)	(5.9)	(11.8)	(0.0)	(0.0)
Hypomagnesemia	2	2	0	0	0	2	2	1	1	0
	(10.5)	(10.5)	(0.0)	(0.0)	(0.0)	(11.8)	(11.8)	(5.9)	(5.9)	(0.0)
Hypophosphatemia	0	0	0	0	0	0	0	1	0	0
	(0.0)	(0.0)	(0.0)	(0.0)	(0.0)	(0.0)	(0.0)	(5.9)	(0.0)	(0.0)
Musculoskeletal and Connective Tissue Disorders	3	1	0	0	0	4	0	2	0	0
	(15.8)	(5.3)	(0.0)	(0.0)	(0.0)	(23.5)	(0.0)	(11.8)	(0.0)	(0.0)
Pain in extremity	0	1	0	0	0	1	0	1	0	0
	(0.0)	(5.3)	(0.0)	(0.0)	(0.0)	(5.9)	(0.0)	(5.9)	(0.0)	(0.0)
Musculoskeletal and connective tissue disorder - other	0	0	0	0	0	0	0	1	0	0
	(0.0)	(0.0)	(0.0)	(0.0)	(0.0)	(0.0)	(0.0)	(5.9)	(0.0)	(0.0)
Renal and Urinary Disorders	7	1	1	0	0	3	0	0	0	0
	(36.8)	(5.3)	(5.3)	(0.0)	(0.0)	(17.6)	(0.0)	(0.0)	(0.0)	(0.0)
Chronic kidney disease	0	0	1	0	0	0	0	0	0	0
	(0.0)	(0.0)	(5.3)	(0.0)	(0.0)	(0.0)	(0.0)	(0.0)	(0.0)	(0.0)
Reproductive System and Breast Disorders	4	1	1	0	0	4	1	2	0	0
	(21.1)	(5.3)	(5.3)	(0.0)	(0.0)	(23.5)	(5.9)	(11.8)	(0.0)	(0.0)
Pelvic pain	3	0	0	0	0	1	0	1	0	0

	(15.8)	(0.0)	(0.0)	(0.0)	(0.0)	(5.9)	(0.0)	(5.9)	(0.0)	(0.0)
Vaginal hemorrhage	2	1	1	0	0	3	1	1	0	0
	(10.5)	(5.3)	(5.3)	(0.0)	(0.0)	(17.6)	(5.9)	(5.9)	(0.0)	(0.0)
Vaginal perforation	0	0	0	0	0	0	0	1	0	0
	(0.0)	(0.0)	(0.0)	(0.0)	(0.0)	(0.0)	(0.0)	(5.9)	(0.0)	(0.0)

Supplementary Table 2: Highest Grade Adverse Events Regardless of Attribution to Protocol Therapy

NRG ONCOLOGY
NRG-GY017
(ClinicalTrials.gov NCT #__________)

TITLE
Anti PD-L1 (Atezolizumab) as an Immune Primer and Concurrently with Extended Field Chemoradiotherapy for Node Positive Locally Advanced Cervical Cancer.
NCI Version Date: 10/02/2018

This trial is part of the National Clinical Trials Network (NCTN) program, which is sponsored by the National Cancer Institute (NCI). The trial will be led by NRG Oncology.

This study is limited to select NRG Oncology Phase I approved participating sites listed on page 3

Coordinating Center:
NRG Oncology
Four Penn Center; 1600 JFK Blvd; Suite 1020
Philadelphia, PA 19103

**Study Team**	
**Study Chair**	**Study Co-Chair/Mentor**
**Jyoti Mayadev M.D.**	**Russell Schilder, M.D.**
University of California San Diego	Thomas Jefferson University
3855 Health Sciences Drive, #0865	1025 Walnut Street, Suite 700
La Jolla, CA 92093-0865	Philadelphia, PA 19107
858-822-6040/FAX 858-246-1505	(215) 503-3057/FAX: (215) 503-3408
jmayadev@ucsd.edu	russell.schilder@jefferson.edu
**Medical Oncology Co-Chair and Translational Research Co-Chair**	**Statistician**
Dmitriy Zamarin, M.D., Ph.D.	**Wei Deng, Ph.D.**
Memorial Sloan Kettering Cancer Center	NRG Oncology SDMC Buffalo<br>Roswell Park Cancer Ctr.
300 E. 66th Street	Elm and Carlton Streets
New York, NY 10065	Buffalo, NY 14263
646-888-4882/FAX: 646-888-4265	716-854-5702/FAX: 716-845-8393
zamarind@mskcc.org	dengw@nrgoncology.org

NRG Oncology Contact Information	
**Research Nurse**	**Translational Scientist**
**Amy Boutelle, RN.**	**Heather A Lankes, PhD, MPH**
University of California San Diego	NRG BB-Columbus
3855 Health Sciences Drive, #0865	Biopathology Center
La Jolla, CA 92093-0865	Nationwide Children's Hospital
858-822-6040/FAX 858-246-1505	700 Children's Drive
aboutelle@ucsd.edu	Columbus, OH 43205
	lankesh@nrgoncology.org
**Data Manager**	**Radiation Oncology Physicist**
**Dolores Kirschner**	HaYeon Kim, Ph.D.
201 N. Craig St., Suite 500	Department of Radiation Oncology
Pittsburgh, PA 15213	University of Pittsburgh
412-383-4916/FAX: 412-624-1082	300 Halket Street
kirschnerd@nrgoncology.org	Pittsburg, PA 15213
	412-641-4602
**Disease Site Chair**	kimh2@upmc.edu
**Carol Aghajanian, M.D.**	
Memorial Sloan Kettering Cancer Center	**Protocol Coordinator**
300 E 66th Street	**Nicole Copeland, M.A.**
New York, NY 10065	NRG Oncology
646-888-4217	Four Penn Ctr, 1600 JFK Blvd, Ste 1020
646-888-4267	Philadelphia, PA 19103
aghajanc@MSKCC.org	215-854-0770/FAX: 215-854-0716
	copelandn@nrgoncology.org
**RTQA:**	**Dosimetrist**
For questions concerning RT data submission	Susan McNulty, BS, RT (R)(T), CMD
	IROC Phila
	1818 Market Street, Ste. 1720
	Philadelphia, PA 19103
	215-940-8907
	smcnulty@acr.org
**RTQA:**	
For questions concerning RT credentialing	http://irochouston.mdanderson.org
	OR iroc-credentialing@mdanderson.org
**RTQA:**	
For questions concerning data submission to TRIAD	Triad-support@acr.org

NRG Oncology Participating Institutions	
PA075	Abramson Cancer Center at the University of Pennsylvania
AZ151	Arizona Cancer Center
OH027	Cleveland Clinic Foundation
PA086	Fox Chase Cancer Center
MD017	Johns Hopkins University/Sidney Kimmel Cancer Center
GA020	Georgia Regents University
CT009	Hartford Hospital
TX035	M.D. Anderson Cancer Center
NY016	Memorial Sloan Kettering Cancer Center
WI013	Froedtert and the Medical College of Wisconsin
OH007	Ohio State University
OK003	Oklahoma University
NY158	Roswell Park Cancer Institute
PA121	Thomas Jefferson University
CA189	UC Davis Medical Center
CA088<br>CA	University of California Medical Center at Irvine-Orange Campus<br>University of California San Diego Medical Center
OH274	UHHS – Chagrin Highlands Medical Center
IL057	University of Chicago
CO070	University of Colorado Cancer Center
OH029	Case Western Reserve University
IA018	University of Iowa Hospitals and Clinics
PA015	University of Pittsburgh Cancer Center
VA009	University of Virginia Health Systems
VA010	Virginia Commonwealth University
MO011	Washington University School of Medicine
RI012	Women's and Infants Hospital

Protocol Agents

**Agent**	**Supply**	**NSC #**	**IND #**	**IND Sponsor**
Atezolizumab (MPDL3280A)	CTEP/PMB	783608	134427	DCTD, NCI
Cisplatin	Commercial	119875		

Participating Sites

☒ U.S.

☐ Canada

☐ Approved International Member Sites

Document History

	**Version Date**
	**Month, Day, Year**
Activation	
Pre-Activation	

This protocol was designed and developed by NRG Oncology. It is intended to be used only in conjunction with institution-specific IRB approval for study entry. No other use or reproduction is authorized by NRG Oncology nor does NRG Oncology assume any responsibility for unauthorized use of this protocol.

NRG ONCOLOGY
NRG GY017
Anti PD-L1 (Atezolizumab) as an Immune Primer and Concurrently with Extended Field Chemoradiotherapy for Node Positive Locally Advanced Cervical Cancer

<table>
<tr><th colspan="3">CONTACT INFORMATION</th></tr>
<tr><td>For regulatory requirements:</td><td>For patient enrollments:</td><td>For study data submission:</td></tr>
<tr><td>Regulatory documentation must be submitted to the CTSU via the Regulatory Submission Portal:

Regulatory Submission Portal (Sign in at www.ctsu.org, and select the Regulatory Submission sub-tab under the Regulatory tab.)

Institutions with patients waiting that are unable to use the Portal should alert the CTSU Regulatory Office immediately at 1-866-651-2878 to receive further instruction and support.

Contact the CTSU Regulatory Help Desk at 1-866-651-2878 for regulatory assistance.</td><td>Please refer to the patient enrollment section of the protocol for instructions on using the Oncology Patient Enrollment Network (OPEN) which can be accessed at https://www.ctsu.org/OPEN_SYSTEM/ or https://OPEN.ctsu.org.

Contact the CTSU Help Desk with any OPEN-related questions at ctsucontact@westat.com.</td><td>Data collection for this study will be done exclusively through Medidata Rave. Please see the data submission section of the protocol for further instructions.</td></tr>
<tr><td colspan="3">The most current version of the study protocol and all supporting documents must be downloaded from the protocol-specific Web page of the CTSU Member Web site located at https://www.ctsu.org. Access to the CTSU members' website is managed through the Cancer Therapy and Evaluation Program - Identity and Access Management (CTEP-IAM) registration system and requires user log on with CTEP-IAM username and password. Permission to view and download this protocol and its supporting documents is restricted and is based on person and site roster assignment housed in the CTSU RSS.</td></tr>
<tr><td colspan="3"><u>For clinical questions (i.e. patient eligibility or treatment-related)</u> contact the Study PI of the Lead Protocol Organization.</td></tr>
<tr><td colspan="3"><u>For non-clinical questions (i.e. unrelated to patient eligibility, treatment, or clinical data submission)</u> contact the CTSU Help Desk by phone or e-mail:
CTSU General Information Line – 1-888-823-5923, or ctsucontact@westat.com. All calls and correspondence will be triaged to the appropriate CTSU representative.</td></tr>
<tr><td colspan="3">The CTSU Website is located at https://www.ctsu.org.</td></tr>
</table>

TABLE OF CONTENTS

1. OBJECTIVES 9
- 1.1 Primary Objective 9
- 1.2 Secondary Objectives 9
- 1.3 Exploratory Objectives 9

2. BACKGROUND 9
- 2.1 Cervical Cancer Background 9
- 2.2 Immunology of HPV Infection and Cervical Cancer 10
- 2.3 Clinical Safety Data 11
- 2.4 Rationale for Extended Field Intensity Modulated Radiation Therapy 11
- 2.5 Rationale for Image Guided Brachytherapy (IGBT) 12
- 2.6 Rationale for the PET imaging post treatment as exploratory endpoint 12

3. PATIENT SELECTION, ELIGIBILITY, AND INELIGIBILITY CRITERIA 12
- 3.1 Patient Selection Guidelines 12
- 3.2 Eligibility Criteria 13
- 3.3 Ineligibility Criteria 14

4. REQUIREMENTS FOR STUDY ENTRY, TREATMENT, AND FOLLOW-UP 19
- 4.1 PRE-TREATMENT ASSESSMENTS 19
- 4.2 ASSESSMENTS DURING TREATMENT 20
- 4.3 ASSESSMENTS IN FOLLOW UP 20
- 4.4 Study Calendar 21

5. TREATMENT PLAN/Regimen description 22
- 5.1 Chemotherapy 25
- 5.2 Radiation Therapy 27
- 5.3 Imaging 42
- 5.4 Integral Assay/Biomarker 43
- 5.5 General Concomitant Medication and Supportive Care Guidelines 43
- 5.6 Duration of Therapy 44
- 5.7 Dose Limiting Toxicity 45

6. TREATMENT MODIFICATIONS/managEment 47
- 6.1 Cisplatin Dose Modifications 47
- 6.2 Atezolizumab Dose Modification and Toxicity Management Guidelines 49
- 6.3 Radiation Therapy Dose Delivery Modifications 64

7. ADVERSE EVENTS REPORTING REQUIREMENTS 65
- 7.1 Protocol Agents 65
- 7.2 Adverse Events and Serious Adverse Events 65
- 7.3 Comprehensive Adverse Events and Potential Risks (CAEPR) List 66
- 7.4 Adverse Events for Commercial Study Agents 69
- 7.5 Adverse Event Characteristics 70
- 7.6 Expedited Reporting of Adverse Events 70

8. REGISTRATION AND STUDY ENTRY PROCEDURES 73
8.1 CTEP Registration Procedures 73
8.2 CTSU Registration Procedures 74
8.3 Patient Enrollment 76
8.4 RT-Specific Pre-Registration Requirements 77

9. DRUG INFORMATION 79
9.1 Atezolizumab (NSC 783608) 79
9.2 Cisplatin (NSC# 119875) 82

10. PATHOLOGY/BIOSPECIMEN 83
10.1 Central Pathology Review Guidelines 83
10.2 Biomarker Selection for Integral Biomarker Testing 83
10.3 Biospecimen Selection for Integrated Biomarker Testing 84
10.4 Biospecimen Submission Tables 85

11. Modality Reviews 87

12. DATA AND RECORDS 87
12.1 Data Management/Collection 87
12.2 Summary of Data Submission 88
12.3 Global Reporting/Monitoring 88

13. STATISTICAL CONSIDERATIONS 89
13.1 Study Design 89
13.2 Study Endpoints 89
13.3 Primary Objectives Study Design 89
13.4 Study Monitoring of Primary Objectives 92
13.5 Accrual/Study Duration Considerations 92
13.6 Dose Level Guidelines 92
13.7 Secondary or Exploratory Endpoints (including correlative science aims) 92
13.8 Exploratory Hypothesis and Endpoints 95
13.9 Gender/Ethnicity/Race Distribution 95

14. REFERENCES 96

APPENDIX I – TRANSLATIONAL SCIENCE BIOSPECIMEN PROCEDURES 104

APPENDIX II – PERFORMANCE STATUS CRITERIA 110

APPENDIX III – RADIATION THERAPY CONTOURS 111

APPENDIX IV – Letter to pathologists 115

APPENDIX V - BIOPSY PATHOLOGY VERIFICATION 116

APPENDIX VI – NCI/DCTD Collaborative Agreements Language 117

NRG GY017
SCHEMA

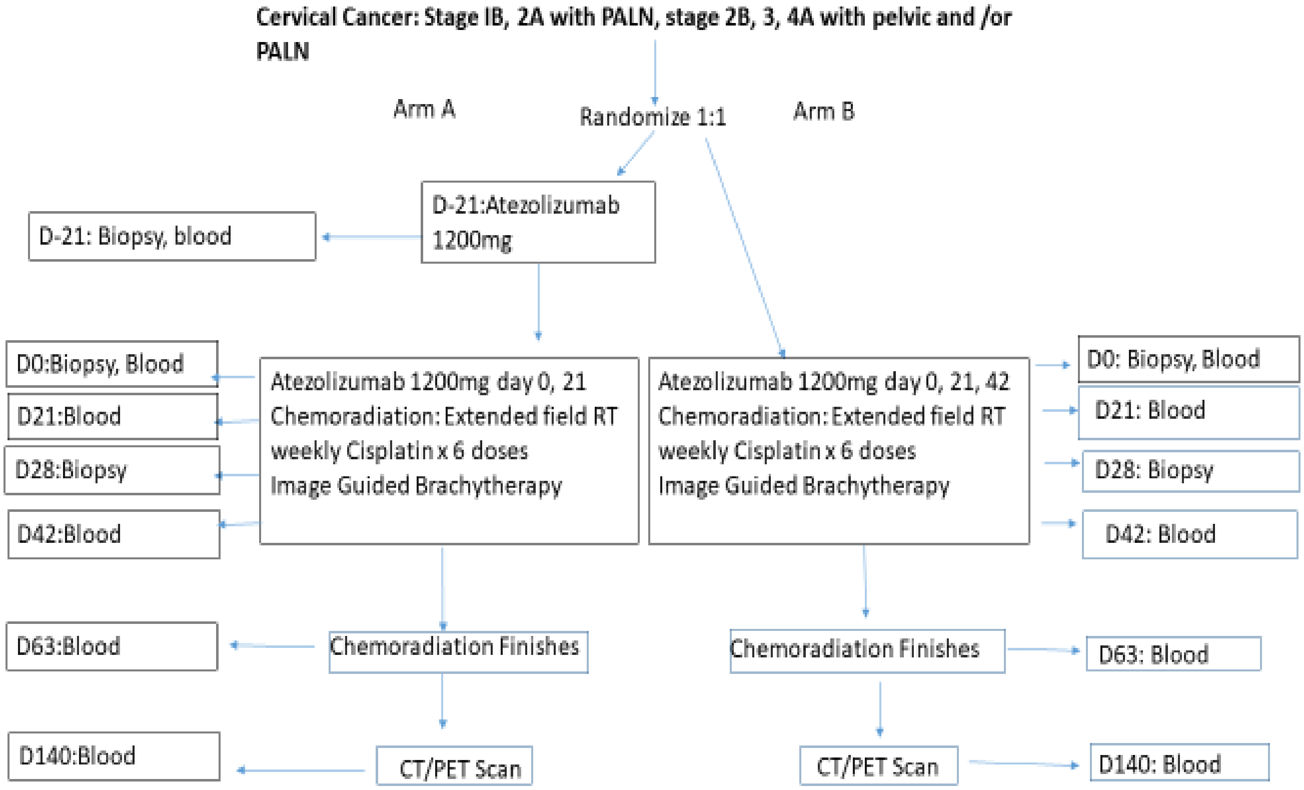

1. OBJECTIVES

1.1 Primary Objective

1.1.1 To determine whether differences in sequencing of atezolizumab and chemoradiation result in differential immune activation, as determined by clonal expansion of T cell receptor beta (TCRB) repertoires in peripheral blood on day 21.

1.2 Secondary Objectives

1.2.1 To investigate the feasibility of administration of the anti PD-L1 antibody (atezolizumab) as an immune primer and concurrent with chemoradiation (CRT) therapy in patients with locally advanced cervical cancer.

1.2.2 To determine the nature and degree of toxicity of the anti PD-L1 antibody (atezolizumab) administered as an immune primer and concurrent with chemoradiation (CRT) therapy in patients with locally advanced cervical cancer.

1.2.3 To examine the changes in TCR clonality, diversity, and frequency in peripheral blood and tissue and correlate this with clinical outcomes, such as the exploratory response assessment on the post-treatment PET-CT scan and 2-year disease-free survival (DFS).

1.2.4 To assess the predictive value of baseline and on-treatment PD-L1 expression in the tissue in each treatment arm for clinical outcomes using post-treatment PET-CT scan and 2-year DFS as the outcome measures.

1.3 Exploratory Objectives

1.3.1 To explore baseline and on-treatment blood and tissue biomarkers that could predict response to the combination therapy, as correlated to the exploratory clinical endpoint of the week 12 (day 140) PET –CT scan and 2-year DFS.

1.3.2 To explore the response assessment on the exploratory and optional post-treatment week 12 (day 140) PET-CT scan and the clinical 2-year disease free survival (DFS).

2. BACKGROUND

2.1 Cervical Cancer Background

Cervical cancer affects an estimated 12,900 women and accounts for an estimated 4,100 deaths in the United States, and 266,000 deaths globally each year. Locally advanced cervical cancer is defined as stage IB2 (tumor confined to the cervix measuring >4cm), stage II (tumor invading beyond the uterus or upper 2/3 of vagina), stage III (tumor extending to pelvic sidewall, lower 1/3 of vagina, or causing hydronephrosis), and stage IVA (tumor invading mucosa of bladder or rectum or extending beyond the true pelvis). Patients diagnosed with locally-advanced cervical cancer have a higher risk of recurrence and worse survival than the early stage patients. After upfront surgery, the relapse rate in all stages exceeds 30 percent, with 5-year survival of 30% in patients with stage III disease [1,2]. Patients presenting with para-aortic lymph node (PALN) metastases at diagnosis represent a particularly poor prognostic group with 5 year survival of approximately 40% across the stages[3]. The integration of chemotherapy, particularly cisplatin with radiotherapy, demonstrated improved survival rates, resulting in a paradigm shift in care for locally advanced cervical cancer [4,5]. Despite these advances, patients presenting in the upfront

curative setting with PALN metastasis have a dismal three year overall and progression free interval of 39% and 34%, respectively, even with the use of extended field radiation and concurrent chemotherapy [6]. The role of adjuvant chemotherapy in this setting remains unknown and is being investigated in an international randomized OUTBACK trial, the results of which are still pending. Therefore, there is an unmet need for therapeutic treatment options for patients with PALN, given the dismal survival with current therapeutic options available.

2.2 Immunology of HPV Infection and Cervical Cancer

Cervical cancer has been identified to be direct consequence of infections by specific viral subtypes, namely human papilloma virus (HPV) subtypes 16 and 18 [7-9]. Despite the host immune response to tumors, cancer often develops multiple resistance mechanisms such as local immune suppression, immune evasion, induce dysfunctional T cell mechanisms and immune tolerance, and evasion through endogenous immune checkpoint inhibitors [10].

Anti-PD-L1

PD-L1, also known as B7-H1, the ligand for PD-1 is expressed and has been specifically demonstrated in cervical cancer, as well as in other cancers [11]. Inhibitors to PD-L1 may further restore T cell function and immune T cell priming by blocking not only its interactions with PD-1 but also another family member B7.1 T cell interactions. The study drug, atezolizumab, received breakthrough designation status by the US Food and Drug Administration (FDA) in June 2014 for metastatic bladder cancer, and in February 2015 for the treatment of PD-L1 positive non-small cell lung cancer patients, whose disease has progressed during or after platinum-based chemotherapy (and an appropriate targeted therapy for those with an EGFR mutation-positive or ALK-positive tumor). Atezolizumab has potential promise in locally advanced cervical cancer.

Preliminary Data on Checkpoint Inhibition and Cervical Cancer

GOG-9929, a phase I trial of sequential ipilimumab after CRT for the treatment of patients with locally advanced cervical cancer stages IB2/IIA with positive para-aortic lymph nodes and stage IIB/IIIB/IVA with pelvic/para-aortic lymph nodes as a NCI supported national clinical trial to investigate the maximum tolerated dose (MTD) and dose-limiting toxicities (DLT) of adjuvant ipilimumab following concurrent weekly cisplatin and extended field radiation in women with newly diagnosed locally advanced cervical cancer. When reviewing our outcome data presented at ASCO 2017 at one year on GOG-9929, our one year PFS is 69%, which is impressive when compared to historical three year overall and progression free interval of 39% and 34%, respectively, with extended field radiation and concurrent chemotherapy from previous GOG studies [6]. GOG-9929 data shows a significant increase in PD-1 expressing T cells post chemoradiation. In addition, the KEYNOTE 028 trial, 22 patients with metastatic PDL-1 positive cervical cancer were treated with an anti-PD-1, pembrolizumab, with an overall response rate of 17%, demonstrating acceptable toxicity profile. These data encourage our selection of an anti-PDL-1 study drug in the proposed concept to use a priming dose of anti-PDL-1 therapy prior to and concurrently with chemoradiation for locally advanced cervical cancer.

Radiation and Immune Checkpoint Blockade, Dose and Timing

Optimal results with immunotherapy may occur with multimodality treatment options including dual check point blockade or radiation therapy. Resistance mechanisms of checkpoint blockade can be overcome by regulation of checkpoint inhibition. Early data suggests that the immune system, in particular CD8+ T cells, have a key role for tumor cell death within a radiation field [12,13]. Optimal results with immunotherapy may occur with multimodality treatment options including check point blockade with radiation therapy. Radiation therapy causes migration of dendritic cells, cross-penetration of tumor antigens which can result in T cell activation and proliferation [14]. Furthermore, radiation therapy increases the density of tumor infiltrating lymphocytes (TIL) within a tumor likely by extravasation of TIL within the vasculature of tumors and chemokines activation [14,15]. It is known that radiation therapy alters the T cell repertoire of peripheral T cell clones [16]. There are limited data surrounding the optimal dose and fractionation needed to provoke an ideal immune response when combining immunotherapy with radiation. Immunotherapy and sequencing data with radiation demonstrate that when aCTLA-4 is delivered prior to RT there is increased efficacy compared to delivery after RT, or if alone and not in combination with RT [17].

2.3 Clinical Safety Data

Our results from GOG-9929 show that sequential anti-CTLA4 after CRT is tolerable. Given that these cervical cancer patients are treated with an extended field radiation and the entire pelvis, patients were treated with sequential ipilimumab after lymphocyte count toxicities resolved to a grade 1 within 2-6 weeks post CRT. Our data show that two patients in the maximum tolerated dose level (10mg/kg) in the cohort expansion experienced a reversible grade 3 toxicity – grade 3 lipase, and skin rash. Given the toxicity data using an anti PD-1, and anti PDL-1 across various disease sites, we expect that the use of atezolizumab and CRT will be tolerable. To further reduce bowel toxicity, we plan to use an intensity modulated radiation therapy (IMRT) approach as already explored in NRG-GY006. The radiation and physics quality assurance pathway used in NRG-GY006 will be also used in the development of the proposed study for safety and quality radiation delivery

2.4 Rationale for Extended Field Intensity Modulated Radiation Therapy

Extended field IMRT is an effective radiation delivery approach to treat PALN positive and high risk pelvic LN positive cervical cancer [18,19]. Multiple published studies indicate that IMRT can reduce both hematologic and gastrointestinal (GI) toxicities, and can provide excellent long-term outcomes [20,21]. A prospective study of locally advanced cervical cancer patients treated with EFRT and cisplatin based chemotherapy showed an improvement in the 3-year actuarial overall survival, disease-free survival, and distant metastasis-free survival : 87% versus 62% (P = 0.02), 82% versus 54% (P = 0.02), and 79% versus 57% (P = 0.01), versus pelvic radiation only historical controls [18]. Furthermore, a study from the University of Pittsburg showed that in 61 patients with PALN positive or high risk for microscopic disease, EFRT with IMRT was tolerable with a late grade 3+ adverse event rate of 4% [19]. Furthermore, they found that EFRT with IMRT resulted in low regional recurrence in node-positive cervical cancer [19]. In a large retrospective review, cervical cancer patients with positive PALN treated with definitive extended-field radiation therapy showed that IMRT improved disease specific survival and a decrease in treatment related adverse effects compared to patients not treated with IMRT [22].

2.5 Rationale for Image Guided Brachytherapy (IGBT)

Brachytherapy is a key component in the treatment of cervical cancer as it allows for dose escalation to the tumor while minimizing the dose to surrounding critical organs at risk such as the sigmoid, bladder, and rectum. Patterns of care studies established the essential role of brachytherapy in the management of cervical cancer, and linked its use to improvements in pelvic control and disease free survival [23]. Furthermore, within the last decade, the advent of image-guided brachytherapy (IGBT) for both applicator insertion and treatment planning has increased the procedural implantation and treatment planning options for the treatment of cervical cancer [24]. With the widespread use of sophisticated imaging modalities in radiation oncology departments, the majority of brachytherapy centers use 3D imaging for contouring and treatment planning in brachytherapy [25] [25-32]. The American Brachytherapy Society recently investigated the use of image guided brachytherapy showing an improvement in pelvic control and DFS compared to studies with conventional 2D based imaging technology in patients treated with CRT for locally advanced cervical cancer [33].

2.6 Rationale for the PET imaging post treatment as exploratory endpoint

In the definitive treatment of cervical cancer, 12 week post therapy positron emission tomography (PET) with F-18 fluorodeoxyglucose (FDG) is predictive of survival outcome (Schwartz et al. 2007). We investigated the role of the post immunotherapy positron emission tomography (PET) as a surrogate biomarker for survival for cervical cancer patients (pts) treated with standard chemoradiation (CRT) followed by ipilimumab on GOG-9929. The PET was obtained from 4-12 weeks. Thirty-two of 34 enrolled pts initiated study treatment, of whom 15 pts have complete pre and post therapy PET data, with at least one dose of ipilimumab. Of the 15 pts with post-ipilimumab PET scan data, 93% were squamous histology; all had positive pelvic lymph nodes, with 47% having positive paraaortic lymph nodes in addition. All 15 pts completed CRT, with ipilimumab completion rates of 73% with 4 cycles, 13% with 3 cycles, 7% with 2 cycles and 7% with 1 cycle. Eleven had a metabolic CR, two had stable disease, and two had a PR. With a median follow up of 12 months for these 15 pts, one patient with CR on PET had a recurrence. We plan to investigate the role of post therapy PET for response assessment.

3. PATIENT SELECTION, ELIGIBILITY, AND INELIGIBILITY CRITERIA

Note: Per NCI guidelines, exceptions to inclusion and exclusion criteria are not permitted. For questions concerning eligibility, please contact the Biostatistical/Data Management Center (see protocol cover page). For radiation therapy-related eligibility questions, please contact RTQA (see protocol cover page).

3.1 Patient Selection Guidelines

Although the guidelines provided below are not inclusion/exclusion criteria, investigators should consider these factors when selecting patients for this trial. Investigators also should consider all other relevant factors (medical and non-medical), as well as the risks and benefits of the study therapy, when deciding if a patient is an appropriate candidate for this trial.

3.1.1 Submission of tumor biopsies is required for all patients. Investigators should check with their site pathology department regarding release of biospecimens before approaching patients about participation in the trial.

3.2 Eligibility Criteria

A patient cannot be considered eligible for this study unless ALL of the following conditions are met.

3.2.1 Patients with histologically confirmed newly diagnosed advanced cervical cancer (squamous cell carcinoma, adenocarcinoma, and adenosquamous cell carcinoma): FIGO clinical stages IB2/IIA with positive para-aortic nodes, or FIGO clinical stages IIB/IIIB/IVA with positive pelvic or para-aortic lymph nodes (PALN). Pelvic or PALN nodal status confirmed by PET/CT scan or fine needle biopsy or extra peritoneal biopsy or laparoscopic biopsy. The PALN must be inferior to the T12/L1 interspace.

3.2.2 ECOG performance status ≤2 (Karnofsky ≥60%, see Appendix II).

3.2.3 Patients must have normal organ and marrow function as defined below:

- leukocytes ≥2,500/mcL
- absolute neutrophil count ≥1,500/mcL
- platelets ≥100,000/mcL (≥50,000 for patients with hematologic malignancies)
- hemoglobin ≥8 g/dL (can be transfused with red blood cells pre-study)
- total bilirubin ≤1.5 × institutional upper limit of normal (ULN) (however, patients with known Gilbert disease who have serum bilirubin level ≤3 × ULN may be enrolled)
- AST(SGOT)/ALT(SGPT) ≤3 × ULN (AST and/or ALT ≤5 × ULN for patients with liver involvement)
- alkaline phosphatase ≤2.5 × ULN (≤5 × ULN for patients with documented liver involvement or bone metastases)
- creatinine clearance ≤1.5 mg/dL to receive weekly Cisplatin*

*Patients whose serum creatinine is between 1.5 and 1.9 mg/dL are eligible for cisplatin if there is no hydronephrosis and the estimated creatinine clearance (CCr) is ≥30 ml/min. For the purpose of estimating the CCr, the formula of Cockcroft and Gault for females should be used:

$$CCr = \frac{0.85 \text{ x } (140 - \text{age}) \text{ x IBW}}{(\text{Scr x } 72)}$$

where age is the patient's age in years (from 20 to 80 years), Scr is the serum creatinine in mg/dL, and IBW is the ideal body weight in kg (according to the calculation IBW = 45.5 kg + 2.3 kg for each inch over 5 feet).

- INR and aPTT — ≤1.5 × ULN (This applies only to patients who **do not** receive therapeutic anticoagulation; patients receiving therapeutic anticoagulation, such as low-molecular-weight heparin or warfarin, should be on a stable dose.)

3.2.4 Age is $\geq$ 18 years.

3.2.5 Patient does not have a known allergy to cisplatin or compounds of similar biologic composition.

3.2.6 Patient is not actively breastfeeding (or has agreed to discontinue breastfeeding before the initiation or protocol therapy).

3.2.7 TSH within normal limits or normal free T4 in those with abnormal TSH.

3.2.8 Ability to understand and the willingness to sign a written informed consent document.

3.2.9 Patients positive for human immunodeficiency virus (HIV) are allowed on study, but HIV-positive patients must have:

- A stable regimen of highly active anti-retroviral therapy (HAART)
- No requirement for concurrent antibiotics or antifungal agents for the prevention of opportunistic infections
- A CD4 count above 250 cells/mcL and an undetectable HIV viral load on standard PCR-based tests.

3.3 Ineligibility Criteria

Patients with any of the following conditions are NOT eligible for this study.

3.3.1 Patients who have received prior radiation therapy to the pelvis or abdominal cavity, PALN radiation, or previous therapy of any kind for this malignancy or pelvic, PALN, or abdominal radiation for any prior malignancy.

3.3.2 Patients with PALN nodal metastasis above the T12/L1 interspace.

3.3.3 Patients who had a radical hysterectomy with positive PALNs are not eligible.

3.3.4 Patients with prior allogeneic bone marrow transplantation or prior solid organ transplantation.

3.3.5 **Patients previously treated with systemic anticancer therapy** (*e.g.*, chemotherapy, targeted therapy, immunotherapy) within 3 years prior to entering the study.

3.3.6 Treatment with systemic immunosuppressive medications (including, but not limited to, prednisone, cyclophosphamide, azathioprine, methotrexate, thalidomide, and anti-tumor necrosis factor [anti-TNF] agents) within 2 weeks prior to Cycle 1, Day 1.

- Patients who have received acute, low dose, systemic immunosuppressant medications (*e.g.*, a one-time dose of dexamethasone for nausea or steroids as CT scan contrast premedication) may be enrolled.
- The use of inhaled corticosteroids and mineralocorticoids (*e.g.*, fludrocortisone) for patients with orthostatic hypotension or adrenocortical insufficiency is allowed.

3.3.7 Known hypersensitivity to Chinese hamster ovary cell products or other recombinant human antibodies.

3.3.8 History of severe allergic, anaphylactic, or other hypersensitivity reactions to chimeric or humanized antibodies or fusion proteins.

3.3.9 Patients requiring treatment with a RANKL inhibitor (e.g., denosumab) who cannot discontinue it before treatment with atezolizumab.

3.3.10 Known clinically significant liver disease, including active viral, alcoholic, or other hepatitis; cirrhosis; fatty liver; and inherited liver disease

- Patients with past or resolved hepatitis B infection (defined as having a negative hepatitis B surface antigen [HBsAg] test and a positive anti-HBc [antibody to hepatitis B core antigen] antibody test) are eligible.
- Patients positive for hepatitis C virus (HCV) antibody

3.3.11 History or risk of autoimmune disease, including but not limited to systemic lupus erythematosus, rheumatoid arthritis, inflammatory bowel disease, vascular thrombosis associated with antiphospholipid syndrome, Wegener's granulomatosis, Sjögren's syndrome, Bell's palsy, Guillain-Barré syndrome, multiple sclerosis, autoimmune thyroid disease, vasculitis, or glomerulonephritis.

- Patients with a history of autoimmune hypothyroidism on a stable dose of thyroid replacement hormone are eligible.
- Patients with controlled Type 1 diabetes mellitus on a stable insulin regimen or Type 2 diabetes mellitus are eligible.
- Patients with eczema, psoriasis, lichen simplex chronicus or vitiligo with dermatologic manifestations only (e.g., patients with psoriatic arthritis would be excluded) are permitted provided that they meet the following conditions:
  - Patients with psoriasis must have a baseline ophthalmologic exam to rule out ocular manifestations
  - Rash must cover less than 10% of body surface area (BSA)
  - Disease is well controlled at baseline and only requiring low potency topical steroids (e.g., hydrocortisone 2.5%, hydrocortisone butyrate 0.1%, flucinolone 0.01%, desonide 0.05%, alclometasone dipropionate 0.05%)
  - No acute exacerbations of underlying condition within the last 12 months (not requiring psoralen plus ultraviolet A radiation [PUVA],

methotrexate, retinoids, biologic agents, oral calcineurin inhibitors; high potency or steroids)

3.3.12 History of idiopathic pulmonary fibrosis, pneumonitis (including drug induced), organizing pneumonia (i.e., bronchiolitis obliterans, cryptogenic organizing pneumonia, etc.), or evidence of active pneumonitis on screening chest computed tomography (CT) scan. History of radiation pneumonitis in the radiation field (fibrosis) is permitted.

3.3.13 Patients with active tuberculosis (TB) are excluded.

3.3.14 Severe infections within 4 weeks prior to Cycle 1, Day 1, including but not limited to hospitalization for complications of infection, bacteremia, or severe pneumonia.

3.3.15 Signs or symptoms of infection within 2 weeks prior to Cycle 1, Day 1.

3.3.16 Received intravenous (IV) antibiotics within 2 weeks prior to Cycle 1, Day 1. Patients receiving prophylactic antibiotics (*e.g.*, for prevention of a urinary tract infection or chronic obstructive pulmonary disease) are eligible.

3.3.17 Major surgical procedure within 28 days prior to Cycle 1, Day 1 or anticipation of need for a major surgical procedure during the course of the study.

3.3.18 Administration of a live, attenuated vaccine within 4 weeks before Cycle 1, Day 1 or anticipation that such a live, attenuated vaccine will be required during the study and up to 5 months after the last dose of atezolizumab.

- Influenza vaccination should be given during influenza season only (approximately October to March). Patients must not receive live, attenuated influenza vaccine within 4 weeks prior to Cycle 1, Day 1 or at any time during the study.

3.3.19 Uncontrolled intercurrent illness including, but not limited to, ongoing or active infection, symptomatic congestive heart failure, unstable angina pectoris, cardiac arrhythmia, or psychiatric illness/social situations that would limit compliance with study requirements.

3.3.20 Severe, active co-morbidity defined as follows:

- Current (within 28 days of Cycle 1, Day 1) signs and/or symptoms of bowel obstruction
- Patients who require parental hydration and/or nutrition
- Patients who require drainage gastrostomy tube
- Evidence of bleeding diathesis or clinically significant coagulopathy
- Serious, non-healing or dehiscing wound, active ulcer or untreated bone fracture
- History of hemoptysis (≥ 1/2 teaspoon of bright red blood per episode) within 1 month of study enrollment

3.3.21 Significant cardiovascular or cerebrovascular disease including:

- Uncontrolled hypertension (SBP $\geq$150; DBP $\geq$90)
- History of myocardial infarction within 6 months
- Unstable angina
- New York Heart Association functional classification II, III or IV
- Baseline ejection fraction ≤ 50% as assessed by echocardiogram or MUGA
- Cerebral vascular accident (CVA) or transient ischemic attack (TIA) within 6 months
- Significant vascular disease (e.g., aortic aneurysm, requiring surgical repair or peripheral arterial thrombosis) within 6 months

3.3.22 History of abdominal/pelvic or tracheoesophageal fistula or gastrointestinal perforation and/or abscess within 6 months prior to initiation of treatment.

3.3.23 If patients are of child-bearing potential and do not agree to use two forms of birth control then they are ineligible.

3.3.24 Patients who have had a hysterectomy or are planning to have an adjuvant hysterectomy following radiation as part of their cervical cancer treatment are ineligible.

3.3.25 Patients scheduled to be treated with adjuvant consolidation chemotherapy at the conclusion of their standard chemoradiation.

3.3.26 Pregnant women are excluded from this study because radiation therapy has the potential for teratogenic or abortifacient effects. Because there is an unknown but potential risk for adverse events in nursing infants secondary to treatment of the mother with Atezolizumab, breastfeeding should be discontinued if the mother is treated with Atezolizumab.

3.3.27 Women of child-bearing potential and men must agree to use adequate contraception (hormonal or barrier method of birth control; abstinence) prior to study entry, for the duration of study participation, and for the 5 months (150 days) after the last dose of the study agent. Should a woman become pregnant or suspect she is pregnant while she or her partner is participating in this study, she should inform her treating physician immediately.

3.3.28 Patients taking bisphosphonate therapy for symptomatic hypercalcemia. Use of bisphosphonate therapy for other reasons (*e.g.*osteoporosis) is allowed.

3.3.29 Patients with known primary central nervous system (CNS) malignancy or CNS metastases are excluded.

3.3.30 Patients with a prior known history of vesicovaginal, enterovaginal or colonvaginal Fistula.

4. REQUIREMENTS FOR STUDY ENTRY, TREATMENT, AND FOLLOW-UP

4.1 PRE-TREATMENT ASSESSMENTS

Assessments	Prior to Registration (calendar days)	Prior to treatment start
History and Physical	≤ 14 days	≤ 14 days
Concomitant Medications	≤ 14 days	≤ 14 days
Vital Signs	≤ 14 days	≤ 14 days
Performance Status	≤ 14 days	≤ 14 days
Toxicity Assessment	≤ 14 days	≤ 14 days
CBC/Differential/Platelets	≤ 14 days	≤ 14 days
Chemistries (BUN, Creatinine, Sodium, Potassium, Chloride, CO2, Calcium, Glucose, Bilirubin, Protein, Albumin, Alkaline Phosphatase, AST, ALT)	≤ 14 days	≤ 14 days
TSH	≤ 14 days	≤ 28 days
Hepatitis B Surface Antigen	≤ 28 days	≤ 28 days
Hepatitis B Core Antibody	≤ 28 days	≤ 28 days
Hepatitis C Antibody	≤ 28 days	≤ 28 days
UPCR	≤ 14 days	≤ 14 days
INR and aPTT§	≤ 28 days	≤ 28 days
Pregnancy Test (if childbearing potential exists)	≤ 7days	≤ 7 days
EKG (Electrocardiogram)	≤ 28 days	≤ 28 days
PET/CT scan	≤ 28 days	≤ 28 days
Radiographic Tumor Measurement*	≤ 28 days	≤ 28 days

§ For patients not receiving therapeutic anticoagulation.
*Radiographic tumor measurements should be obtained via imaging of the chest, abdomen and pelvis to establish the location and extent of disease. See RECIST 1.1 for allowable imaging modalities used to assess disease at baseline (and subsequent assessments). Contrast CT is the preferred modality.

4.2 ASSESSMENTS DURING TREATMENT

**Assessments**	**Prior to each treatment of Atezolizumab**
History and Physical	X
Concomitant Medications*	X
Corticosteroid Use (only to manage adverse events)	X
Secondary Immunosuppressive Agent Use (e.g., infliximab)	X
Vital Signs	X
Performance Status	X
Toxicity Assessment	X
CBC/Differential/Platelets	≤ 3 days
Chemistries (BUN, Creatinine, Sodium, Potassium, Chloride, CO2, Calcium, Glucose, Bilirubin, Protein, Albumin, Alkaline Phosphatase, AST, ALT, Amylase, Lipase)	≤ 3 days
TSH	≤ 7 days

* **Due to their immunosuppressive effect, administration of systemic steroids (e.g., dexamethasone) as an antiemetic and/or preparative regimen for hypersensitivity reactions should be avoided if other means of treatment are available and medically appropriate.**

4.3 ASSESSMENTS IN FOLLOW UP

**Assessments**	**End of treatment, 1 month and 3 months, q3 months for 2 years**
Medical history, physical examination, ECOG performance status, vital signs (heart rate, blood pressure, respiratory rate, temperature), and vital status (including any recurrence)	1 month and 3 months, q 3 months for 2 years
Toxicity Assessment	1 month and 3 months, q 3 months for 2 years
Radiographic tumor measurement	3 months, 1 year, 2 year
CBC / Differential / Platelets<br>Chemistries (including sodium, potassium, chloride, bicarbonate, calcium, glucose, magnesium, BUN/creatinine, total bilirubin, total protein, ALT, AST, alkaline phosphatase, albumin), TSH<br><br>PET/CT, pre-scan glucose-OPTIONAL	1 month and 3 months<br><br>3 months (optional)

4.4 Study Calendar

ARM A

	Pre-Study	Day -21	Day 0	Day 7	Day 14	Day 21	Day 28	Day 35	Day 42	Day 49	CRT done 56-60	1 mo post 1	3 mo post 3	Off Study[c]
Atezolizumab		X	X			X								
Cisplatin			X	X	X	X	X	X						
Radiation			X	X	X	X	X	X	X	X	X			
Brachytherapy						X	X	X	X	X	X			
Informed consent	X													
Demographics	X													
Medical history	X	X	X	X	X	X	X	X	X	X				
Physical exam	X	X	X	X	X	X	X	X	X	X		X	X	X
Vital signs	X	X	X	X	X	X	X	X	X	X		X	X	X
Height	X	X	X	X	X	X	X	X	X	X		X	X	X
Weight	X	X	X	X	X	X	X	X	X	X		X	X	X
Performance status	X	X	X	X	X	X	X	X	X	X		X	X	X
CBC w/diff, plts	X	X	X	X	X	X	X	X	X	X		X	X	X
Serum chemistry	X	X	X	X	X	X	X	X	X	X		X	X	X
EKG	X		X			X						X	X	
Adverse event evaluation	X	X	X	X	X	X	X	X	X	X		X	X	X
CT	X												X**	
PET/CT	X												X**	
B-HCG	X[b]													
Research Biopsy	X	X	X				X*							
Resarch Blood Draw		X	X			X			X		X		X	

X*: Biopsy to be obtained at the time of the first brachytherapy procedure (prior to brachytherapy dose delivery)

X**: 3 month post CRT +/- 7 days (OPTIONAL)

b: Serum pregnancy test (women of childbearing potential).

c: Off-study evaluation.

ARM B

Arm B														
	Pre-Study		Day 0	Day 7	Day 14	Day 21	Day 28	Day 35	Day 42	Day 49	CRT done 56-60	1 mo post 1	3 mo post 3	Off Study[c]
Atezolizumab			X			X			X					
Cisplatin			X	X	X	X	X	X						
Radiation			X	X	X	X	X	X	X	X	X			
Brachytherapy						X	X	X	X	X	X			
Informed consent	X													
Demographics	X													
Medical history	X		X	X	X	X	X	X	X	X				
Physical exam	X		X	X	X	X	X	X	X	X		X	X	X
Vital signs	X		X	X	X	X	X	X	X	X		X	X	X
Height	X		X	X	X	X	X	X	X	X		X	X	X
Weight	X		X	X	X	X	X	X	X	X		X	X	X
Performance status	X		X	X	X	X	X	X	X	X		X	X	X
CBC w/diff, plts	X		X	X	X	X	X	X	X	X		X	X	X
Serum chemistry	X		X	X	X	X	X	X	X	X		X	X	X
EKG	X		X			X			X			X	X	
Adverse event evaluation	X		X	X	X	X	X	X	X	X		X	X	X
CT	X												X**	
PET/CT	X												X**	
B-HCG	X[b]													
Research Biopsy	X		X				X*							
Resarch Blood Draw			X			X			X		X		X	

X*: Biopsy to be obtained at the time of the first brachytherapy procedure (prior to brachytherapy dose delivery)

X**: 3 month post CRT +/- 7 days (OPTIONAL)

b: Serum pregnancy test (women of childbearing potential).

c: Off-study evaluation.

5. TREATMENT PLAN/REGIMEN DESCRIPTION

This study has two study arms:

Arm A: will get one dose of atezolizumab prior to cisplatin chemotherapy and radiation therapy, and then two subsequent doses of atezolizumab during the chemotherapy and radiation therapy

Regimen Description Arm A					
Agent	***Pre-medications; Precautions***	***Dose***	***Route***	***Schedule***	***Cycle Length***
Atezolizumab	Preparative antihistamine use is allowed (e.g., diphenhydramine 25 mg IV)	1200 mg	IV infusion delivered over 60 (±15) minutes. If the first infusion is tolerated without	Arm A: - 21 days, Days 0, 21	Every 3 weeks dosing

			infusion associated AEs, the second infusion may be delivered over 30 (±10) minutes		
Cisplatin	Increased oral intake of fluid should be encouraged 24 hours prior to infusion; 1000 ml of ½ normal saline infused IV one hour before cisplatin. Antiemetic prophylaxis with a serotonin antagonist and/or dexamethasone 10-20 mg IV is allowed as a pre-medication.	40 mg/m^2 (70 mg maximum) diluted in 250-1000 ml normal saline, reconstitution results in a colorless solution. Mixing and administration should follow local standards	IV infusion at a rate of 1mg/min, usually infusing over 1 ½ hours (90 minutes), using non-aluminum administration sets; after is an additional 1000 ml of ½ normal saline infused over one hour	Days 0, 7, 14, 21, 28, 35	One time weekly (**preferably before**, *but allowed to be after* pelvic radiation treatment)
Pelvic and PALN external beam radiation therapy	Skin, antiemetic, or anti-diarrheal medications may be administered as needed.	1.8 Gy/ day	See RT section	Daily (excludes weekends and holidays)	Daily (Start on a Monday is preferred)
Image Guided Brachy-therapy	Antiemetic and anti-diarrheal medication may be administered as needed.	Minimum 85 Gy to CTV (EQD2) for cervical	See RT section	See RT section	See RT Section

Arm B: will get 3 doses of atezolizumab during the cisplatin chemotherapy and radiation therapy

Regimen Description Arm B					
Agent	***Pre-medications; Precautions***	***Dose***	***Route***	***Schedule***	***Cycle Length***
Atezolizumab	Preparative antihistamine use is allowed (e.g.,	1200 mg	IV infusion delivered over 60 (±15)	Arm B: Days 0,	Every 3 weeks dosing

	diphenhydramine 25 mg IV)		minutes. If the first infusion is tolerated without infusion associated AEs, the second infusion may be delivered over 30 (±10) minutes	21, 42	
Cisplatin	Increased oral intake of fluid should be encouraged 24 hours prior to infusion; 1000 ml of ½ normal saline infused IV one hour before cisplatin. Antiemetic prophylaxis with a serotonin antagonist and/or dexamethasone 10-20 mg IV is allowed as a pre-medication.	40 mg/m^2 (70 mg maximum) diluted in 250-1000 ml normal saline, reconstitution results in a colorless solution. Mixing and administration should follow local standards	IV infusion at a rate of 1mg/min, usually infusing over 1 ½ hours (90 minutes), using non-aluminum administration sets; after is an additional 1000 ml of ½ normal saline infused over one hour	Days 0, 7, 14, 21, 28, 35	One time weekly (**preferably before**, *but allowed to be after* pelvic radiation treatment)
Pelvic and PALN external beam radiation therapy	Skin, antiemetic, or anti-diarrheal medications may be administered as needed.	1.8 Gy/ day	See RT section	Daily (excludes weekends and holidays)	Daily (Start on a Monday is preferred)
Image Guided Brachy-therapy	Antiemetic and anti-diarrheal medication may be administered as needed.	Minimum 85 Gy to CTV (EQD2) for cervical	See RT section	See RT section	See RT Section

Definition of Disease Assessments

Response and progression will be evaluated in this study using the new international criteria

proposed by the revised Response Evaluation Criteria in Solid Tumors (RECIST) guideline (version 1.1) [*Eur J Ca* 45:228-247, 2009]. Changes in the largest diameter (unidimensional measurement) of the tumor lesions and the shortest diameter in the case of malignant lymph nodes are used in the RECIST criteria.

5.1 Chemotherapy

5.1.1 Cisplatin Chemotherapy

All patients will receive six one-time weekly intravenous infusions of cisplatin 40 mg/m^2 (70mg maximum) diluted in 250-1000 mL normal saline. The cisplatin infusions occur on days 0, 7, 14, 21, 28, and 35. To accommodate scheduling issues, cisplatin administration is allowed to be after pelvic radiation treatment, but is preferably done prior to PRT. Increased oral intake of fluid should be encouraged 24 hours prior to cisplatin infusion. It is recommended that 1000 ml of ½ normal saline be infused intravenously one hour before cisplatin infusion, but other institutional protocols are accepted. Antiemetic prophylaxis with a serotonin antagonist is allowed as a pre-medication. It is recommended that the cisplatin intravenous infusion proceed at a rate of 1mg/minute, usually infusing over 90 minutes. Other infusion rates are acceptable per institutional routine. Non-aluminum intravenous administration sets are required. An additional 1000 ml of ½ normal saline should be infused intravenously over one hour after cisplatin infusion, but other institutional protocols are accepted.

5.1.2 Atezolizumab

Administration of atezolizumab will be performed in a setting with emergency medical facilities and staff who are trained to monitor for and respond to medical emergencies.

All patients will receive three intravenous infusions of atezolizumab 1200 mg every three weeks. Arm A will receive atezolizumab treatment on days -21, 0, and 21. Arm B will receive atezolizumab on days 0, 21, 42. The atezolizumab will be delivered during the post-cisplatin hydration on the days when both drugs are administered on Arm A: days 0, and 21; Arm B: days 0, 21, 42.

The initial dose of atezolizumab will be delivered over 60 (±15) minutes. If the first infusion is tolerated without infusion associated AEs, the second infusion may be delivered over 30 (±10) minutes.

For the first infusion, the patient's vital signs (heart rate, respiratory rate, blood pressure, and temperature) should be determined within 60 minutes before, during (every 15 [±5] minutes), and 30 (±10) minutes after the infusion. For subsequent infusions, vital signs will be collected within 60 minutes before and within 30 minutes after the infusion. Vital signs should be collected during the infusion only if clinically indicated. Patients will be informed about the possibility of delayed post-infusion symptoms and instructed to contact their study physician if they develop such symptoms.

Premedication is not permitted for the first dose of atezolizumab. Preparative antihistamine use is allowed at the discretion of the treating physician. The management

of Infusion Related Reactions will be according to severity as follows:

- In the event that a patient experiences a Grade 1 Infusion Related Reaction during the first treatment, the infusion rate should be reduced to half the rate being given at the time of event onset. Once the event has resolved, the investigator should wait for 30 minutes while delivering the infusion at the reduced rate. If tolerated, the infusion rate may then be increased to the original rate.

- In the event that a patient experiences a Grade 2 Infusion Related Reaction, or flushing, fever, or throat pain, the infusion should be immediately interrupted and the patient should receive aggressive symptomatic treatment. The infusion should be restarted only after the symptoms have adequately resolved to baseline grade. The infusion rate at restart should be half of the infusion rate that was in progress at the time of the onset of the Infusion Related Reaction. For subsequent infusions, administer oral premedication with antihistamine and anti-pyretic and monitor closely for Infusion Related Reactions.

- For Grade 3 or 4 Infusion Related Reactions, the infusion should be stopped immediately, and aggressive resuscitation and supportive measures should be initiated (*e.g.*, oral or IV antihistamine, anti-pyretic, glucocorticoids, epinephrine, bronchodilators, oxygen). Atezolizumab should be permanently discontinued. Resumption of atezolizumab may be considered in patients who are deriving benefit and have fully recovered from the immune-related event; retreatment requires consultation with, and consent of, the trial Principal Investigator (PI).

For anaphylaxis precautions, use the following procedure:

<u>Equipment Needed</u>

- Tourniquet
- Oxygen
- Epinephrine for subcutaneous, intravenous, and/or endotracheal use in accordance with standard practice
- Antihistamines
- Corticosteroids
- Intravenous infusion solutions, tubing, catheters, and tape

<u>Procedures</u>

In the event of a suspected anaphylactic reaction during atezolizumab infusion, the following procedures should be performed:

1. Stop the study drug infusion.
2. Apply a tourniquet proximal to the injection site to slow systemic absorption of study drug. Do not obstruct arterial flow in the limb.
3. Maintain an adequate airway.

4. Administer antihistamines, epinephrine, or other medications as required by patient status and directed by the physician in charge.
5. Continue to observe the patient and document observation.

5.2 Radiation Therapy

Please note that all cases require a pre-treatment review. Please see section 11

General Principals

This protocol requires photon external beam radiation therapy (EBRT), with extended field intensity modulated radiation therapy (IMRT) techniques followed by low dose rate (LDR), pulsed dose rate (PDR), or high dose rate (HDR) brachytherapy. All radiation therapy must be completed ideally within 56 days of initiation (less than or equal to 59 days for compliance).

The external beam radiation is given via IMRT with the exception of the parametrial boost to be 3D conformal RT or IMRT. If field size to cover the target measures larger than institution's linear accelerator largest field size for clinical use, a two-isocenter technique is recommended to use for planning. The prescription dose is 45 Gy in 25 fractions at 1.8 Gy/fraction, unless a simultaneous integrated boost (SIB) is required for gross nodal disease. For SIB cases, the gross nodal PTV boost will receive 50 Gy in 2 Gy/fraction, and then receive a sequential boost dose of 2 Gy per fraction for a total dose of 54-58Gy depending on the contribution of the brachytherapy and location of the lymph node or parametrial boosts.

IMRT should be given once daily Monday-Friday, 5 fractions per week.

For brachytherapy, the prescription doses are 27.5 - 30 Gy for HDR and 35 - 40 Gy for LDR or PDR, following institutional protocol with image guided brachytherapy.

If clips are present from the lymph node dissection to document the position of the lymph nodes, then these should be used as a guide when anterior blocks are designed to shield small bowel.

5.2.1 Treatment Technology

<u>The following treatment modalities include photons with IMRT, and image guided brachytherapy.</u>

External Beam Radiation Therapy (EBRT): Pelvic and PALN: Extended field RT (EFRT)

Pelvis and PALN radiotherapy will be delivered via IMRT. Plans may include static field arrangements (e.g. 5-9 fields), modulated arc therapy, or TomoTherapy. A pseudo-step-wedge intensity modulation (PSWIM) technique is permitted. IMRT should use 4-18 MV photons.

Parametrial and/or Nodal Boost
A parametrial boost can be delivered at the treating physician's discretion and it can be accomplished via 3DCRT or IMRT. The nodal boosts will be determined by the treating

physician in reference to the diagnostic CT or PET scan. The boost level of 54Gy or 58Gy is determined relative to the brachytherapy contribution in the pelvis.

Brachytherapy
High dose rate (HDR), pulsed dose rate (PDR), or low dose rate (LDR) brachytherapy is permitted. Either standard (point-directed using volumetric brachytherapy imaging or planning for organs at risk) or volume-directed brachytherapy techniques are permitted according to each institution's standard. Volume-directed brachytherapy is encouraged. Intracavitary (e.g., tandem and ovoids or tandem and ring) and/or interstitial (e.g., interstitial applicators) brachytherapy is permitted.

5.2.2 Immobilization and Simulation

3D planning is required for this protocol. A CT simulation scan is required with a slice thickness ≤ 3.0 mm for the regions extending at least 4cm above and below target volumes. Patients can be simulated supine or prone. It is recommended that CT scans be obtained from the T10 vertebral body to 5 cm below the ischial tuberosities. All subjects will have a customized immobilization device (e.g., Alpha Cradle or Vac-Loc) fabricated at the time of simulation. Intravenous contrast is recommended to visualize vessels better. Oral or rectal contrast is used at the discretion of the radiation oncologist for treatment planning purposes. Intravenous contrast is recommended unless medically contraindicated.

A full bladder is intended to be comfortably filled on the treatment planning CT scan and throughout the treatment. Patients are asked to drink 300-500 ml of liquid roughly 45 mins prior to the CT simulation and treatment. The rectum and sigmoid should be as empty as possible. The patient should be asked to empty the stools before scanning and treatment. If an internal target volume (ITV) will be used, it is recommended that the patient undergo both a full and empty bladder CT simulation.

The inferior extent or vaginal extension of disease should be marked so that the inferior border of disease can be documented. Uninvolved normal tissues may be blocked although the position of the uterus should be contoured to ensure adequate coverage.

5.2.3 Imaging for Structure Definition, Image Registration/Fusion

Pelvic MRI or PET fusion with the CT simulation scan is recommended to aid target delineation. Fusion should be optimized to match the MRI/PET scans to the treatment position.

5.2.4 Target Volume Definition and Nomenclature

All structures must be named for digital RT data submission as listed in the tables below. Capital letters, spacing and use of underscores must be applied exactly as indicated. Resubmission of data may be required if labeling of structures does not conform to the standard DICOM name listed. The structures marked as "Required" in the table must be contoured and submitted with the treatment plan. Structures marked as "Required when applicable" must be contoured and

submitted when applicable.

The Gross Tumor Volume (GTV), Clinical Target Volume (CTV) and Planning Target Volume (PTV) will be contoured on all CT slices in which the structures exist.

The definition of all volumes will be in accordance with the 1993 ICRU Report #50: Prescribing, Recording and Reporting Photon Beam Therapy and with the 1999 ICRU Report #62: Prescribing, Recording and Reporting Photon Beam Therapy (Supplement to ICRU Report #50).

Please see the attached Appendix III Radiation Contours for examples of:

**Standard Name**	**Description**	**Detailed Specification**
GTV_4500	GTV to receive 45 Gy **Required**	The GTV is defined as all known gross cervical disease determined from radiographic studies, clinical information, physical examination, endoscopic examination, and biopsy results.
CTV_4500	CTV to receive 45 Gy **Required**	It is recommended to divide the CTV into 3 sub regions: CTVn (nodal), and CTVp1 and CTVp2 (primary). CTVp1 will consist of the gross tumor, cervix, and uterus, ovaries and tubes; CTVp2 consists of the parametria and superior third of the vagina (or half of the vagina, if the vagina is clinically involved). CTVn will include the PALN region to the vertebral level of L1/L2 interspace or 3 cm cranial to gross PALN disease, common, external, and internal iliac and presacral lymph nodes. It is acceptable to include the mesorectal nodes in CTVn. The upper border of the CTVn should not extend above the confluence of the common iliac arteries with the aorta (i.e., aortic bifurcation), and should begin no lower than superior border of L5. The CTVn will be obtained by ensuring an approximately 7 mm margin around the vessels, plus extension to include any adjacent visible lymph nodes, lymphoceles, or pertinent surgical clips. The presacral nodes should be contoured until the superior border of the S3

		vertebral body is reached; below this point the nodal volume can be separated into two structures. The external iliac nodes should be contoured to the superior aspect of the femoral head. CTVn should be modified to exclude bone, muscle, and bowel. The CTV should not extend inferior to the ischial tuberosities.
CTV_5000<br>(for SIB schemes only)	CTV to receive 50 Gy<br>**Required**	Gross pelvic LN or PALN. This consists of the gross nodal area as defined by PET and CT with a 7mm expansion around the node or inclusive of clinical extension.<br><br>If a patient is to be treated with a parametrial boost, this area is included in the CTV_5000
CTV_5400<br>(for SIB schemes only)<br><br><br>CTV_5800 (for SIB schemes only)	CTV to receive 54Gy<br>**Required**<br><br><br>CTV to receive 58Gy<br>**Required**	Gross pelvic lymph nodes (obturator LN, internal or external iliac LN) that will receive BT contribution. Parametrial boosts will be included in this volume.<br><br><br>Gross PALN or pelvic lymph nodes (common iliac high or low internal or external iliac LN) that will NOT receive BT contribution<br><br>Parametrial boost volumes will NOT be included in this volume.
ITV_4500	ITV to receive 45 Gy<br>**Contouring is required only when ITV approach is used.**	Patients should be simulated with both a full and empty bladder (i.e., 2 simulation scans). CTVp and CTVn and PTVp and PTVn should be delineated as described above on the plan used for treatment (either the full or empty bladder scan). The CTVp from both scans should be fused together to generate the ITV_4500. A 7 mm margin should be applied to generate PTVi.
PTV_4500	PTV to receive 45 Gy<br>**Required**	Around CTVp1, a 15 mm uniform expansion should be used. Around CTVp2, a 10mm uniform expansion

		should be used. Around CTVn, a 7 mm uniform expansion should be used. These expansions will generate PTVp1, PTVp2, and PTVn. PTVn and PTVp1 and PTVp2 will be fused to generate the PTV_4500. If the ITV approach is used, PTVi should be fused with PTVn to generate PTV_4500.
PTV_4500-03	PTV - 3 mm from skin surface<br>**Required when applicable**	The PTV should be manually or automatically trimmed up to 3 mm from the skin surface, if necessary, to spare skin. However, the CTV still needs to be included entirely within the PTV.
PTV_5000<br>(for SIB schemes only)	PTV to receive 50 Gy<br>**Required**	Around CTV_5000, a 7mm expansion is used
PTV_5000-03 (for SIB schemes only)	PTV - 3 mm from skin surface<br>**Required when applicable**	The PTV should be manually or automatically trimmed up to 3 mm from the skin surface, if necessary, to spare skin.. However, the CTV still needs to be included entirely within the PTV.
PTV_5400<br>(for SIB schemes only)<br><br>PTV_5800<br>(for SIB schemes only)	PTV to receive 54.0Gy<br>**Required**<br><br>PTV to receive 58.0Gy<br>**Required**	7 mm uniform expansion around CTV_5400<br><br>7mm uniform expansion around CTV_5800
PTV_5400-03<br>(for SIB schemes only)<br><br>PTV_5800-03<br>(for SIB schemes only)	PTV - 3 mm from skin surface<br>**Required when applicable**<br><br>PTV - 3 mm from skin surface<br>**Required when applicable**	The PTV should be manually or automatically trimmed up to 3 mm from the skin surface, if necessary, to spare skin. However, the CTV still needs to be included entirely within the PTV.<br><br>The PTV should be manually or automatically trimmed up to 3 mm from the skin surface, if necessary, to spare skin. However, the CTV still needs to be included entirely within the PTV.

5.2.5 Critical Structure and Avoidance Structure Nomenclature

All structures must be named for digital RT data submission as listed in the tables below. Capital letters, spacing and use of underscores must be applied exactly as indicated. Resubmission of data may be required if labeling of structures does not conform to the standard DICOM name listed. The structures marked as "Required" in the table must be contoured and submitted with the treatment plan. Structures marked as "Required when applicable" must be contoured and submitted when applicable.

**Standard Name**	**Description**	**Detailed Specification**
External	External patient contour encompassing all patient anatomy with a single contour on each slice **Required**	Normal tissues will be contoured on the CT simulation scan. The tissue within the skin surface and outside all other critical normal structures and the PTV should be contoured on every slice and designated as "External".
E-PTV_4500	All tissue excluding the PTV_4500. Generated by subtracting the PTV receiving a dose 45 Gy from External Contour **Required**	All tissue excluding the PTV_4500, and is generated by subtracting the PTV receiving a dose of 45 Gy from "External".
Spc_Bowel	The space that the bowel may occupy **Required**	Bowel Space will be contoured beginning from the axial slice situated 1 cm superior to the superior-most slice containing PTV (if bowel is not present at this level, the bowel contour will start from its most superior extent), and will continue to its most inferior extent in the pelvis. The Bowel Space will include the outermost extent of the bowel loops plus any space within the abdominal cavity the bowel may occupy. Individual loops of bowel should not be contoured separately. Bowel Space will be outlined on each axial CT slice. Rectum should be contoured separately from bowel.
Rectum	Rectum **Required**	The outer rectal wall will be contoured and filled in, treating the organ as a solid continuous structure, and will be defined from the level of the sigmoid flexure to the anus.

Bladder	Bladder<br>**Required**	The outer bladder wall will be contoured and filled in, treating the organ as a solid continuous structure.
Kidney_R<br><br>Kidney_L	Kidneys<br>**Required**	The outer bone contour will be delineated and filled in, treating the right and left kidney as a solid continuous structure.
SpinalCord<br><br><br><br>Duodenum<br><br><br><br>Liver	Spinal cord<br>**Required**<br><br><br><br>Duodenum<br>**Required**<br><br><br>Liver<br>**Required**	The spinal cord will be contoured and filled in, from the level of T10/T11 to the L1/L2 interspace<br><br><br><br>The duodenum will be contoured and filled in from the distal stomach to the jejunum<br><br><br>The liver will be contoured and filled in
Femur_Base_L	Left femur<br>**Required**	The outer contours of the left femoral base will be delineated and filled in, treating each as a solid continuous structure. Do not include the femoral neck.
Femur_Base_R	Right femur<br>**Required**	The outer contours of the right femoral base will be delineated and filled in, treating each as a solid continuous structure. Do not include the femoral neck.

5.2.6 Dose Prescription

Target Standard Name	Dose (Gy)	Fraction Size (Gy)	Total # of Fractions	Dose Specification Technique
PTV_4500	45	1.8	25	>=95% of PTV receives >= 45 Gy
PTV_5000 (for SIB schemes only)	50	2	25	>=95% of PTV receives >= 50 Gy
PTV_5400<br><br><br>PTV_5800 (for SIB schemes only)	54<br><br><br><br><br>58	2<br><br><br><br><br>2	27<br><br><br><br><br>29	>=95% of PTV receives >= 54 Gy<br>>=95% of PTV receives >= 58 Gy

Pelvic or PALN nodal boost

PALN or pelvic nodal boost is at the discretion of the treating radiation oncologist. Nodal involvement is defined as any pelvic or common iliac nodes if they are either PET positive or their short axis diameter is > 15mm on CT and/or MRI or if nodes are found to be histologically positive on surgical sampling.

The PALN boost will receive a SIB to 50Gy in 2Gy/fraction during the EFRT. After, the LN boost will receive a boost dose of 8Gy in 2Gy/fraction for a total dose of 58Gy.

The pelvic LN boost will receive a SIB to 50Gy in 2Gy/fraction during the EFRT. After, the LN boost will receive a boost dose of EITHER 4Gy in 2Gy/fraction for a total dose of 54Gy, or 8Gy in 2Gy/fraction for a total dose of 58Gy. The location of the pelvic LN and brachytherapy dose contribution will dictate the decision on the nodal boost level of 54Gy or 58Gy. Pelvic LN including the common iliac, high external or internal LN will receive a dose of 58Gy given the minute brachytherapy contribution. Pelvic LN in the obturator space, mid internal iliac or external iliac LN will receive a dose of 54Gy given the brachytherapy contribution.

Parametrial Boost

A parametrial boost can be delivered at the treating physician's discretion. The parametrial boost may be inclusive or in close approximation with a pelvic LN boost. The parametrial boost will be included in the IMRT volumes, specified as CTV_5000 as a SIB. Afterwards, the parametrial boost will go to a final dose of 54Gy in 2Gy/fraction. The volume for the sequential boost will be CTV_5400. This boost can use an IMRT or 3D conformal RT arrangement. In general, the parametrial boost will have the superior border should be reduced to include only the true pelvis and the upper border of the true pelvis is defined as 1 cm above the inferior aspect of the sacroiliac joint. The inferior border remains the same as in the pelvis fields. A parametrial central field block is a minimum of 4 cm wide. If IMRT planning is used, a midline structure should be used to minimize dose where there will be brachytherapy contribution.

5.2.7 Radiation Compliance Criteria

Target Volume Constraints and Compliance Criteria

The dose is prescribed to cover 95% of the PTV. SIB doses of 45Gy and 50Gy with the boost dose to 54Gy and 58Gy. The Dmax per prescription structure is specified in the table below. The PTV_4500 and PTV_5000 will be evaluated prior to the contribution of the boost doses when evaluating the plan compliance as these PTV will contain the boost PTV_5400 and PTV_5800. The PTV_5400 and PTV_5800 will be evaluated from the composite plan.

Normalization of Dose: The plan is normalized such that 95% of the PTV_ volume receives prescription dose.

Note: Deviation Unacceptable occurs when dose limits for Variation Acceptable are not met

Normal Structure Constraints and Compliance Criteria

Name of Structure	Dosimetric parameter	Per Protocol	Variation Acceptable	Deviation Unacceptable
PTV_4500	$D_{95\%}$ (Gy)	>=45	>=43.65	< 43.65
	$D_{97\%}$ (Gy)	>=43.65	>=40.5	< 40.5
	$D_{0.03cc}$ (Gy)	<=51.75	<=54	> 54
PTV_5000	$D_{95\%}$ (Gy)	>=50	>=48.50	< 48.50
	$D_{97\%}$ (Gy)	>=48.50	>= 45	< 45
	$D_{0.03cc}$ (Gy)	<=57.5	<=60	> 60
PTV_5400	$D_{95\%}$ (Gy)	>=54	>=52.4	< 52.4
	$D_{97\%}$ (Gy)	>=52.4	>=48.6	< 48.6
	$D_{0.03cc}$ (Gy)	<=62.10	<=64.8	> 64.8
PTV_5800	$D_{95\%}$ (Gy)	>=58	>=55.1	< 55.1
	$D_{97\%}$ (Gy)	>=56.2	>=52.2	< 52.2
	$D_{0.03cc}$ (Gy)	<=66.7	<=69.6	> 69.6

Name of Structure	Dosimetric parameter	Per Protocol	Variation Acceptable	Deviation Unacceptable
Bladder	$D_{50\%}$ (Gy)	<=45	<=55	> 55
	$D_{0.03cc}$ (Gy)	<=52	<=57.5	> 57.5
Rectum	$D_{50\%}$ (Gy)	<=45	<= 54	> 54
	$D_{0.03cc}$ (Gy)	<=50	<=55	> 55
Bowel	$D_{35\%}$ (Gy)	<=35	<=50	> 50
	$D_{0.03cc}$ (Gy)	<= 60	<=66	> 66
Femurs	$D_{15\%}$ (Gy)	<=30	<=50	> 50
	$D_{0.03cc}$ (Gy)	<=50	<=55	> 55
Bilateral Kidney	$D_{50\%}$ (Gy)	<=18	<=20	> 20
Liver	$D_{50\%}$ (Gy)	<=25	<=30	> 30
Duodenum	$D_{50\%}$ (Gy)	<=40	<= 50	> 50
	$D_{0.03cc}$ (Gy)	<=56	<=60	> 60
Spinal Cord	$D_{0.03cc}$ (Gy)	<=45	<=47.5	> 47.5

5.2.8 Image Guided Brachytherapy

General Principals

All patients will be treated with volume-directed brachytherapy. Either point-directed volumetric planning or volume-directed brachytherapy techniques are permitted according to each institution's standard. CT planning is required with or without MRI guidance.

Brachytherapy dose prescriptions may be to a volumetric structure or point A. If a volume directed target volume is used, the high risk CTV (CTV_HR) will be obtained from the CT planning scan, or with the additional information of a GTV from an MRI. A pelvic MRI is encouraged (≤ 3 mm slice thickness) with either the first or second insertion. A CT and MRI-compatible applicator will be required to perform volume-directed brachytherapy. Subsequent insertions may use CT or MRI (T2 sequence weighting) for planning.

Use of IMRT or other external beam techniques in place of brachytherapy to boost gross cervical disease is expressly discouraged. If deemed necessary or essential for the patient's care (e.g., if a patient refuses brachytherapy), reasons for not performing brachytherapy should be documented and the study PI (Jyoti Mayadev) should be notified.

Either HDR, PDR, or LDR brachytherapy is permitted according to each institution's standard. Institutions must adhere to their declared brachytherapy regimen for all patients treated on this protocol. It is allowed to administer more than one BT fraction by use of the same implant.

Timing and fractionation of Brachytherapy
EBRT or chemotherapy should not be given on the day of HDR brachytherapy. The brachytherapy may start in week 4, 5 or at the end of external beam radiotherapy. Two insertions per week may be done. For LDR brachytherapy, the patient will receive an intracavitary implant in one or two applications after the EBRT. In LDR point-directed brachytherapy, if 2 insertions are used they should be separated by a minimum of 7 days and maximum of 21 days. If 2 insertions are used, the second implant should be completed within three weeks of the completion of external beam irradiation. PDR brachytherapy can be delivered using an Iridium-192 source with the same schedule as LDR. EBRT and brachytherapy may not be administered on the same day.

Brachytherapy Source
Iridium-192 is the preferred source for HDR and PDR brachytherapy. Cesium-137 is the preferred source for LDR brachytherapy. All brachytherapy sources must be listed on the joint AAPM/IROC Houston Registry of Brachytherapy Sources in order to be utilized on NCTN clinical trials (http://irochouston.mdanderson.org/RPC/BrachySeeds/Source_Registry.htm).

Applicators
Brachytherapy applicator selection is based on the discretion of the radiation oncologist to adequately cover the clinical target volume and tumor.
Brachytherapy applications that are acceptable include:
Intracavitary applicators: Tandem and ring or tandem and ovoids
Intracavitary-Interstitial applicators "Hybrid": Tandem and ring with needles; tandem and ovoid with needles
Interstitial applicators: Tandem and cylinders with needles

Simulation
For 3D brachytherapy planning, all subjects will undergo a CT (or PET/CT) simulation scan using a slice thickness ≤ 3.0 mm with brachytherapy applicators and dummy sources in. It is recommended that CT scans contain the entire brachytherapy applicators and the critical

structures such as bladder, rectum, and sigmoid. Maintaining applicator position is required from the time of applicator insertion to simulation and treatment. Bladder contrast and rectal contrast are permitted. In HDR brachytherapy, a fixation device can minimize applicator rotations. Periodic checks during PDR/LDR brachytherapy, and initial and final check in HDR brachytherapy are recommended to monitor changes in the applicator position.

Applicator Reconstruction
Applicator reconstruction will follow institutional protocols. Uncertainties of at least half the slice thickness can be present in applicator reconstruction. Deviations of dose >10% are common for applicator displacement of 3 mm (along intrauterine axis) for CTV_HR(D100, D90) and rectum, bladder and sigmoid (D2cc). Dummy wires or CT reconstructions library plans are optimal for CT based planned. For MRI reconstructions, the CT planning scan may be registered to the MRI dataset by using defined marker points or other registration techniques. Direct reconstruction can be applied in MRI when markers are inserted into the applicator channels.

Brachytherapy Contours
Contouring of GTV (based on the MRI only), CTV_HR, bladder, rectum and sigmoid. No margins should be added to the contours.

Detailed Specifications
Brachytherapy (Volume-directed)
CT or MR images should be used to delineate the target volume. Ideally MRI based target delineation should be used to identify the GTV. The MRI can be reused by superimposition in the process of contouring on CT, if for subsequent fractions of brachytherapy only CT can be used with the applicator in place. No planning margins will be added to CTV_HR.

Target volumes: The definitions of target volumes will be in accordance with the 2013 ICRU Report#89 and GEC-ESTRO/ABS recommendations.

GTV: Macroscopic tumor (if present) at time of brachytherapy
High Risk-CTV (CTV_HR): GTV + whole cervix + extra cervical tumor extension at time of brachytherapy

Structure Name (for volume-directed brachytherapy)	Detailed Specification
GTV **Required**	Macroscopic tumor at time of brachytherapy seen on MRI
CTV_HR **Required**	GTV+ cervix+macroscopic extension or parametrial involvement
Bladder **Required**	The outer bladder wall is contoured as seen on the 3D planning scan. The outer wall is contoured.
Rectum **Required**	The outer rectal wall is contoured from above the anal sphincter to the level of transition into the sigmoid.

Sigmoid **Required**	The outer sigmoid wall is to be contoured from the recto-sigmoid flexure to 2 cm superior to the parametria and the uterus.

ICRU Point Descriptions and Reporting (for point-directed brachytherapy)
The ICRU bladder, rectum and vaginal surface reference point doses must be calculated and reported. Please follow the definitions in ICRU 38.
For either volume-directed or point-directed brachytherapy, the dose to points A for standard application (non-interstitial and non-hybrid cases) must to be documented.

Brachytherapy Dose Optimization

Brachytherapy optimization includes the use of standard loading patterns for dose point optimization, manual loading of dwell times or weight, graphical optimization, or inverse optimization. The physician and physicist should review the optimization based on dose volume histograms, plan review, and dose constraints for further optimization of the brachytherapy plan. For the HDR brachytherapy, the EQD2 dose (Total Biological Equivalent Dose in 2 Gy Fractions) using model assumptions based on the linear quadratic model will be used for the individual treatment planning. An example of the EQD2 worksheet is provided by the American Brachytherapy Society:
https://www.americanbrachytherapy.org/guidelines/gyn_HDR_BT_docu_sheets.xls

Brachytherapy Dose Prescription

In point-directed approach, the doses of 27.5 - 30 Gy and 35 - 40 Gy will be delivered to the point A for HDR and LDR or PDR brachytherapy, respectively as in the tables below. The HDR and PDR brachytherapy dose can be prescribed to a high-risk clinical target volume (CTV_HR) as volume-directed approach, but point A dose must be documented.

HDR-Point A determined implant or volume directed approach

**Total EBRT (Gy)**	**# HDR fractions**	**HDR Point A dose/fraction or CTV_HR (Gy)**	**Total HDR point A dose or CTV_HR (Gy)**	**Total Point A or CTV_HR EQD2-Gy_{10}**
45	4	7.0	28.0	83.9
45	5	5.5	27.5	79.8
45	5	6.0	30.0	84.3

PDR and LDR-Point A determined implant

**Total EBRT (Gy)**	**# LDR fractions**	**LDR Point A dose/fraction (Gy)**	**Total LDR point A dose (Gy)**	**Total Point A dose (Gy)**
45	1	35-40	35-40	80-85
45	2	17.5-20	35-40	80-85

Target and Normal Tissue Compliance Criteria: EBRT + HDR/PDR volume-directed or HDR/PDR/LDR point-directed brachytherapy

Dose contribution from EBRT is assumed to be prescription dose (45Gy) and doses are cumulative EQD2 (α/β=10 for target, α/β=3 for normal tissues) for HDR and physical doses for PDR/LDR.

Name of Structure	Dosimetric parameter	Per Protocol	Variation Acceptable	Deviation Unacceptable
Point A (point-directed)	Point A (Gy)	80-85	> 90	< 75
CTV_HR (volume-directed)	D90% (Gy)	80-90	> 90	
Bladder<br>Bladder *	D_{2cc} (Gy)<br>ICRU 38 pt	<=80<br><=85	<=90<br><=90	> 90<br>>95
Rectum<br>Rectum *	D_{2cc} (Gy)<br>ICRU 38 pt	<=65<br><= 65	<=75<br><=80	> 75
Sigmoid	D_{2cc} (Gy)	<=75	<=75	> 75
Vaginal_Surf *	ICRU 38 pt	<= 150	<=175	

*For Point-directed brachytherapy and ICRU 38 reference point description. (Vaginal_Surf = vaginal surface point)

It is recommended to keep the maximum bowel dose < 25% of the brachytherapy prescription dose. The Point A dose will be recorded but there is no specification on the dose to Point A if volumetric planning is used.

Brachytherapy Volumetric Dose Reporting

For volume-directed brachytherapy, uniform dose volume reporting according to the ICRU Report #89 and GEC-ESTRO recommendations. For each fraction the following parameters should be collected automatically in RAVE when submitting the CT structure set or MR structures set and dose file to TRIAD:

• TRAK
• D100 and D98 for GTV, CTV_HR
• D90 for GTV and CTV_HR
• D50 for CTV_HR
• V100 for CTV_HR
• D2cc of the bladder, rectum, sigmoid and small bowel (for HDR, EQD2 per formula $EQD2= D*[(d + \alpha/\beta)/(2 + \alpha/\beta)]$ using α/β=3)

Delivery Compliance Criteria

	Per Protocol	Variation Acceptable	Deviation Unacceptable
Overall Treatment time	≤ 56 days	≤ 59 days	> 60 days

5.2.9 Radiation Treatment Planning Priorities and Instructions
- Critical Structure and Target priorities are listed in order of decreasing importance

EBRT
1. PTV
2. Bowel
3. Rectum
4. Bladder
5. Femurs

Brachytherapy
1. CTV_HR (if volume-directed plan used)
2. Sigmoid
3. Rectum
4. Bladder

If dose constraints of critical structures are exceeded, the following solution is suggested:
For volume-directed brachytherapy, if the treatment planning system provides a manual optimization option, the plan can be manually optimized to reduce dose to the critical structures as long as D90 of CTV meets target volume constraints. The use of interstitial brachytherapy is sometimes useful to improve the coverage and decrease the OAR dose but this technique should be used when indicated and by trained physicians.

-Required algorithms
<u>(Convolution/Superposition, Monte Carlo, etc.…)</u>
For Convolution/Superposition-type algorithms, dose should be reported as computed inherently by the given algorithm. For Monte Carlo or Grid Based Boltzmann Solver algorithms, conversion of Dm (dose-to-medium) to Dw (dose-to-water) should be avoided. Dm, computed inherently by these algorithms, should be reported.

-Primary dataset for dose calculation
EBRT
The primary data set for dose calculations is CT. In the case in which contrast is present during the treatment planning CT, the density of the contrast should be overrode to a representative background electron density. Heterogeneity corrections must be applied.

Brachytherapy
Planning CT images are the primary dataset for dose calculation. If MR images are used for dose calculation, a method of correcting image distortion and tissue density has to be applied. Heterogeneity corrections are not mandatory.

-Dose matrix resolution
Dose grid size should be ≤ 3 mm (preferably ≤ 2 mm to minimize effects of partial volume averaging) in all directions.

5.2.10 Patient specific QA

For IMRT/VMAT plans, patient-specific QA is highly recommended. Any patient-specific QA performed should follow respective institutional guidelines. The recommended patient specific QA criterion is that 90% of the comparison points pass a ±3%/3 mm Gamma Index analysis as recommended.

5.2.11 Daily Treatment Localization/IGRT

Image-guided radiation therapy (IGRT) is radiation therapy using imaging to facilitate accuracy and precision throughout its entire process from target and normal tissue delineation, to radiation delivery, to adaptation of therapy to anatomic and biological changes over time in individual patients. In this section we use the terminology IGRT to focus on image-guidance at the time of radiation delivery to ensure its adherence to the planned treatment.

EBRT

Daily IGRT is required for this protocol when the IMRT treatment technique is used. Any form of online imaging is acceptable, such as MV or kV planar imaging, MVCT or MV CBCT, kV CBCT, CT on rails, etc. The AAPM recommendations for verifying the coincidence of the imaging and treatment reference points must be adhered to the daily use of IGRT. At the time of simulation, it is recommended to place the isocenter along the patient's midline 1.5 cm caudal to the inferior border of the sacroiliac joint. In general, the CT or CBCT will be used for setup verification using bone landmarks only and not for soft tissue alignment. Small soft tissue shifts (≤ 3 mm) are acceptable. Otherwise, the treating physician may elect to postpone treatment or re-simulate.

Management of Radiation Dose to the Patient from IGRT

NRG Oncology is concerned about the estimated doses given from IGRT, and is committed to limiting the imaging dose when IGRT is used in any of its protocols. This can be accomplished by avoiding the use of this technology to make small changes in patient positioning that are within the stated PTV margins. The imaging dose to the patient may become significant if repeated studies are performed for patients with severe set up problems (e.g. requiring frequent corrections that are larger than the PTV margins). It is recommended that patients demonstrating severe set-up problems during the first week of treatment be moved to a treatment with larger margins.

5.2.12 General Concomitant Medication and Supportive Care Guidelines

Radiation Therapy Supportive Care Guidelines

Patients should receive full supportive care, including transfusions of blood and blood products, supplemental iron, antibiotics, anti-emetics, etc., when appropriate at the discretion of the treating physician. Because it has been observed that hemoglobin levels below 10-12 g/dL during radiotherapy are associated with decreased local control, blood transfusions should be offered to and used to treat patients at the discretion of treating physicians prior to or during radiotherapy. There should be no radiotherapy treatment delays due to a low hemoglobin levels. Trials evaluating

epoeitin alpha (Procrit, Epogen) and radio-chemotherapy in cervical cancer have indicated that epoeitin alpha may be associated with an increased risk for thromboembolism, and thus, may not be used in this study.

In particular, patients should be told to be aware of their typical defecation pattern. At the first sign that their stools become softer than usual or they have any increase in stool frequency over what is normal for them, they should begin taking over-the-counter loperamide as directed by their physician(s). Patient should understand that if they do not start taking loperamide at the start of diarrhea, the diarrhea may become severe and last several days. Persistent diarrhea or any evidence of hematochezia should prompt a stool evaluation for fecal leukocytes and/or *Clostridium difficile* toxin & ova/parasite evaluation. Diphenoxylate/ atropine prescriptions are permitted. Loperamide will be recommended during the study in order to prevent diarrhea that may ultimately lead to intravenous infusion of fluids or hospitalizations.

For the administration of loperamide, it is recommended that patients take two (2) caplets (4 mg) at the first sign of diarrhea. Patients should continue taking one (1) caplet (2 mg) every two hours until they return to their normal pattern of bowel movements. They may repeat same doses and frequency if diarrhea returns. During the night, patients may take two (2) caplets every four hours rather than one (1) every two hours. Patients should call their doctor if they have any questions about taking loperamide, if they believe they are not achieving adequate control of diarrhea, or if they are feeling extremely weak, lightheaded, or dizzy (symptoms of dehydration). Side effects of loperamide include tiredness, drowsiness, or dizziness. If they experience these side effects, they should avoid driving a motorized vehicle or operating machinery. Before using any laxative, patients should consult their physician. Patients should make an extra effort to drink lots of fluids (several glasses of water, fruit juices, soda, soup, etc.) every day while they participate in this study.

5.3 Imaging

5.3.1

- PET/CT scan will be performed as a screening study entry diagnostic criteria. The PET/CT may be performed with a high-quality CT for CT diagnostic purposes. For cases when the PET is not accompanied by a high-quality CT (for example has a lack of or insufficient IV contrast, lack of or insufficient oral / rectal contrast, or lack of a fully diagnostic multiphasic CT imaging), then a separate diagnostic CT of the chest, abdomen, and pelvis will be required for the baseline scan.

•

- The 12-week post therapy PET/CT is optional (Day 140). The PET/CT may be performed with a high-quality CT for diagnostic purposes. For cases when the PET is not accompanied by a high-quality CT (for example has a lack of or insufficient IV contrast, lack of or insufficient oral / rectal contrast, or lack of a fully diagnostic multiphasic CT imaging), then a separate diagnostic CT of the chest, abdomen, and pelvis is optional for this exploratory post therapy 12 week (Day 140) scan.

•

5.4 Integral Assay/Biomarker

5.4.1 See Section 10 for details.

5.5 General Concomitant Medication and Supportive Care Guidelines

5.5.1 Permitted Supportive/Ancillary Care and Concomitant Medications

All supportive therapy for optimal medical care will be given during the study period at the discretion of the attending physician(s) within the parameters of the protocol and documented on each site's source documents as concomitant medication.

- Anticonvulsants
- Antiemetics
- Anticoagulants
- Antidiarrheals
- Analgesics
- Hematopoietic Growth Factors
- Herbal products
- Nutritional supplementation
- Highly active antiretroviral therapy (HAART)

Concomitant therapy includes any prescription medications or over the counter preparations used by a patient between the 7 days preceding the screening evaluation and the treatment discontinuation visit.

Patients who experience infusion-associated symptoms may be treated symptomatically with acetaminophen, ibuprofen, diphenhydramine, and/or cimetidine or another H2 receptor antagonist, as per standard practice (for sites outside the United States, equivalent medications may be substituted per local practice). Serious infusion associated events manifested by dyspnea, hypotension, wheezing, bronchospasm, tachycardia, reduced oxygen saturation, or respiratory distress should be managed with supportive therapies as clinically indicated (*e.g.*, supplemental oxygen and β_2-adrenergic agonists; see **Section 5.1.2**).

Systemic corticosteroids and TNFα inhibitors may attenuate potential beneficial immunologic effects of treatment with atezolizumab but may be administered at the discretion of the treating physician. If feasible, alternatives to corticosteroids should be considered. Premedication may be administered for Cycles $\geq$2 at the discretion of the treating physician. The use of inhaled corticosteroids and mineralocorticoids (*e.g.*, fludrocortisone) for patients with orthostatic hypotension or adrenocortical insufficiency is allowed. Megestrol administered as appetite stimulant is acceptable while the patient is enrolled in the study.

Patients who use oral contraceptives, hormone-replacement therapy, prophylactic or therapeutic anticoagulation therapy (such as low-molecular-weight heparin or warfarin at a stable dose level), or other allowed maintenance therapy should continue their use. Males and females of reproductive potential should use highly effective means of

contraception, defined as a previous history of female sterilization (tubal ligation) or male sterilization (vasectomy), or the use of birth control medications (pill, patch, injectable), diaphragm or ring. The use of male condoms is not a highly effective means of contraception alone.

5.5.2 Prohibited Therapies

Any concomitant therapy intended for the treatment of cancer, whether health authority-approved or experimental, is prohibited unless it is specifically included in the treatment regimen described in this protocol. This includes but is not limited to the following:

- Chemotherapy, hormonal therapy, immunotherapy, radiotherapy, investigational agents, or herbal therapy (except for maintenance therapies outlined in **Section** Error! Reference source not found.).
    - After Cycle 1, certain forms of radiotherapy may be considered for pain palliation if patients are deriving benefit (*e.g.*, treatment of known bony metastases); atezolizumab administration may be suspended during radiotherapy.

It is strongly recommended that:

- Traditional herbal medicines not be administered because the ingredients of many herbal medicines are not fully studied and their use may result in unanticipated drug-drug interactions that may cause, or confound assessment of, toxicity.
- The use of a RANKL inhibitor (denosumab) be discontinued during the study; this agent could potentially alter the activity and the safety of atezolizumab.

Initiation or increased dose of granulocyte colony-stimulating factors (*e.g.*, granulocyte colony-stimulating factor, granulocyte/macrophage colony-stimulating factor, and/or pegfilgrastim) is prohibited for patients with solid malignancies.

Patients are not allowed to receive immunostimulatory agents, including, but not limited to, IFN-α, IFN-γ, or IL-2, during the entire study. These agents, in combination with atezolizumab, could potentially increase the risk for autoimmune conditions.

Patients should also not be receiving immunosuppressive medications, including, but not limited to, cyclophosphamide, azathioprine, methotrexate, and thalidomide. These agents could potentially alter the activity and the safety of atezolizumab. Systemic corticosteroids and anti-TNFα agents may attenuate potential beneficial immunologic effects of treatment with atezolizumab but may be administered at the discretion of the treating physician. If feasible, alternatives to these agents should be considered.

5.6 Duration of Therapy

In the absence of treatment delays due to adverse event(s), treatment may continue as specified in the above treatment modality sections or until one of the following criteria applies:

- Disease progression,
- Intercurrent illness that prevents further administration of treatment,
- Unacceptable adverse event(s), as described in Section 6

- Patient decides to withdraw consent for participation in the study, or
- General or specific changes in the patient's condition render the patient unacceptable for further treatment in the judgment of the investigator.

5.7 Dose Limiting Toxicity

Dose-limiting toxicities (DLTs) will be defined by drug related adverse effects that meet the criteria below as evaluated by NCI CTCAE v.5 unless clearly unrelated to study therapy (e.g., disease progression). As this regimen is being developed as a front-line therapy, the goal is for patients to complete all treatments. Thus, all cycles will be considered in determining DLT. Specifically, the DLT period for Arm A: start of the priming dose of atezolizumab until 30 days after the completion of CRT; for Arm B: start of CRT until 30 days after the completion of CRT.

A DLT includes:

- Any Grade 4 immune related adverse event
- Any >= grade 3 immune related colitis
- Any Grade 3 or 4 noninfectious pneumonitis (irrespective of duration)
- Any Grade 2 noninfectious pneumonitis that does not resolve to <= grade 1 within 7 days of the initiation of maximal supportive care
- Liver transaminase elevation > 8 x ULN or total bilirubin > 5 x ULN
- Any >= grade 3 non immune related adverse event except for the exclusion list below
- Delay of weekly cisplatin of greater than 2 weeks due to failure to recover AE/failure to meet protocol directed treatment parameters for restart after management of AE
- Any treatment related death.

A DLT does not include:

- Grade 3 infusion reaction to atezolizumab or cisplatin therapy that is medically managed
- Grade 3 fatigue, anorexia or constipation lasting less than one week
- Grade 3 endocrine disorders (thyroid, pituitary, and/or adrenal insufficiency) that is managed with or without systemic corticosteroid therapy and/or hormone replace therapy and the subject is asymptomatic
- Grade 3 infusion related reaction
- Grade 3 or 4 neutropenia (that is not associated with fever or infection) lasting <=7 days
- Grade 3 or 4 lymphopenia
- Grade 3 thrombocytopenia that is not associated with clinically significant bleeding
- Isolated grade 3 electrolyte abnormalities that are not associated with clinical signs or symptoms and are reversed with appropriate maximal medical intervention
- Grade 3 hypertension
- Grade 3 nausea and/or vomiting (maximal medical management should be employed) lasting < 48 hours
- Grade 3 dehydration as a result of nausea or vomiting lasting < 48 hours
- Grade 3 and 4 asymptomatic amylase/lipase elevation.

Arm A: If > 6 patients (or >30% of DLT-evaluable patients of the 20 enrolled) develop a

DLT during the start of therapy (i.e., start of the priming dose of atezolizumab until 30 days after the completion of CRT), the regimen will be placed on hold and a discussion with the principal investigators and Genentech will be contacted to determine future plans for the study.

Arm B: If > 6 patients (or >30% of DLT-evaluable patients of the 20 enrolled) develop a DLT during the start of therapy (i.e., start of CRT until 30 days after the completion of CRT), the regimen will be placed on hold and a discussion with the principal investigators and Genentech will be contacted to determine future plans for the study.

DLT and other toxicities are discussed among the Study Chair, Co-Chairs, the Phase I Committee Chair, CTEP monitors and the Study Biostatistician. The discussions are the basis for the decisions regarding protocol or treatment modifications according to the rules of the protocol.

For overall evaluation of toxicity, all patients who receive any study drug will be evaluable. For the primary endpoint, all eligible patient who received on Arm A (dose at day -21 and dose at day 0) and Arm B (dose at day 0) and have TCRB measurements at day 21 will be evaluable. For the evaluation of DLT, all eligible patients who have a DLT and have at least one dose of Atezolizumab, or complete protocol therapy until 30 days after the completion of CRT. The DLT toxicity 30% threshold will only include those patients who complete protocol therapy and are evaluable at the DLT specified time period (the DLT period for Arm A: start of the priming dose of atezolizumab until 30 days after the completion of CRT; for Arm B: start of CRT until 30 days after the completion of CRT) if a DLT was not determined prior to this period.

Using a critical value determined by more than 30% of the DLT-evaluable patients having at least one DLT, a power curve is displayed in Figure 5.7 where N is the total number of DLT-evaluable patients, X is the number of patients experiencing at least one DLT, and π is the probability of a patient experiencing at least one DLT. In this study, the maximum number of patients for DLT-evaluable is 20 for each arm. Given a sample size of 20, if π is truly 20%, then probability to observe more than 6 patients with DLT is 8.7%; if π is truly 30%, then probability to observe more than 6 patients with DLT is 39%; if π is truly 40%, then probability to observe more than 6 patients with DLT is 75%.

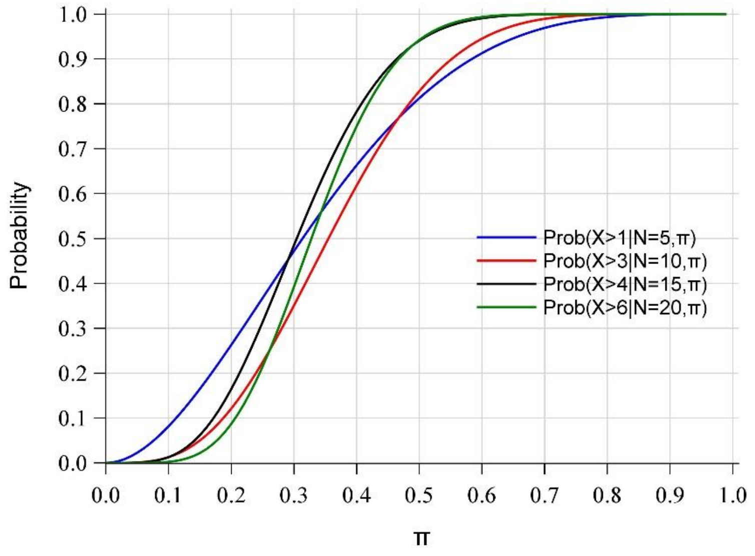

Figure 5.7

6. TREATMENT MODIFICATIONS/MANAGEMENT

6.1 Cisplatin Dose Modifications

Cisplatin dose modifications will be made according to observed symptoms. Chemotherapy will not be administered during a radiation therapy delay.

6.1.1 Gastrointestinal Adverse Effects: (e.g., nausea and vomiting) Prophylactic antiemetics should be used as described above in section 5.1.1. Please follow local institutional standards for patients that develop nausea and vomiting in spite of the use of prophylactic measures. For persistent grade 4 nausea and vomiting despite optimal medical management, hold cisplatin until reduced to grade 1 and reduce cisplatin by 25%. For patients with recurrence of this following dose reduction, cisplatin must be discontinued.

6.1.2 Renal Adverse Effects: If creatinine rises to greater than 2.0 mg/dL, discontinue cisplatin therapy. Selective renal tubular defects are sometimes observed. Documented hypomagnesemia will be treated with increased magnesium supplementation per institutional protocol. Hypocalcemia and hypokalemia are common and potentially severe. Replacement of potassium and calcium are usually effective per institutional protocol. Severe tubular effects may require chronic replacement therapy. Diagnostic tests for other etiology of hypocalcemia (GI or metabolic) should be considered per institutional protocol.

6.1.3 Neurotoxicity Adverse Effects: For grade 2, reduce cisplatin dose by 25%. For grade 3-4, discontinue cisplatin therapy.

6.1.4 Tinnitus or hearing changes: For grade 2 hold cisplatin until neuropathy resolves to grade 1 and dose reduce by 25%. Discontinue cisplatin if tinnitus or hearing changes do not resolve within 21 days. Discontinue cisplatin for grade 3 or 4 toxicity.

6.1.5 Hematologic Adverse Effects: Cisplatin should be withheld from patients with an absolute neutrophil count less than 1500 / mm^3 or platelet count less than 75,000 / mm^3. Cisplatin infusions should be delayed week-by-week until these levels are exceeded. Please refer to the table below for specific hematologic toxicities and recommended modifications.

**HEMATOLOGIC TOXICITY**	**PARAMETER**	**DOSE MODIFICATION**
Anemia	Not applicable for this protocol	No dose modifications for anemia. Please refer to section 5 for additional details.
Febrile Neutropenia or ANC	First occurrence of febrile neutropenia (grade 3 or 4) - OR – ANC < 500 / µL lasting greater than 7 days	Hold cisplatin x 1 week and repeat CBC. If ANC has resolved to grade1 (>1500 / µL), then dose reduce by 25% and treat. If ANC has not resolved to grade 1 then discontinue cisplatin.
Febrile Neutropenia or ANC	Second occurrence	Discontinue cisplatin.
ANC alone	Uncomplicated ANC < 500 / µL lasting greater than 7 days without infection or fever	Dose reduction not indicated, but hold therapy until ANC resolves to grade 1
Platelets without bleeding	Grade 3 uncomplicated	Hold until platelets > 75,000 /µL and resume at current dose level without modification
Platelets with bleeding	First occurrence grade 4 thrombocytopenia –OR – grade 3 thrombocytopenia with bleeding	Hold cisplatin x 1 week and repeat CBC. If platelet count has resolved to > 75,000 / µL, then dose reduce by 25% and treat. If thrombocytopenia has not resolved to grade 1, then discontinue cisplatin
Platelets with bleeding		Discontinue cisplatin

6.1.6 Other non-hematologic toxicity: For any other non-specific grade 3 or 4 toxicity, other than fatigue, hold cisplatin until toxicity resolves to grade 1 and then resume at same dose. If toxicity does not resolve to grade 1 within 21 days, discontinue cisplatin.

6.1.7 External-beam pelvic radiation and atezolizumab infusions should continue while any or all courses of cisplatin are withheld. For delays in cisplatin infusion greater than two weeks, cisplatin may not be continued at the joint discretion of the patient's treating physicians and the principal investigator (face sheet).

6.2 Atezolizumab Dose Modification and Toxicity Management Guidelines

6.2.1 General AE Management and Dose Modification Guidelines

There will be no dose reduction for atezolizumab in this study.

Patients may temporarily suspend study treatment if study drug-related toxicity requiring dose suspension is experienced. If atezolizumab is held because of AEs beyond the scheduled date of infusion, the patient will be discontinued from atezolizumab and will be followed for safety and efficacy as specified in this protocol. If the AE resolves and the patient is receiving corticosteroid therapy for the event, atezolizumab may be held for the remaining doses on study in order to allow tapering of the steroid dose to ≤10 mg oral prednisone or equivalent.

Dose interruptions for reasons other than toxicity, such as surgical procedures, may be allowed. The acceptable length of interruption will be at the discretion of the study PI in consultation with CTEP.

Atezolizumab must be **permanently discontinued** if the patient experiences any of the following events, regardless of benefit:

- Grade 4 pneumonitis
- AST or ALT >5×ULN or total bilirubin >3×ULN
- Grade 4 diarrhea or colitis
- Grade 4 hypophysitis
- Any grade myasthenic syndrome/myasthenia gravis, Guillain-Barré or meningoencephalitis
- Grade 4 ocular inflammatory toxicity
- Grade 4 pancreatitis or any grade of recurrent pancreatitis
- Grade 4 rash
- Any grade myocarditis

Treatment may, under limited and compelling circumstances, be resumed in patients who have recovered from the following events, <u>but only after consultation with the trial Principal Investigator</u>:

- Grade 3 pneumonitis
- Grade 3 ocular inflammatory toxicity
- Grade 3 or 4 infusion-related reactions

Any toxicities associated or possibly associated with atezolizumab treatment should be managed according to standard medical practice. Additional tests, such as autoimmune serology or biopsies, may be used to determine a possible immunogenic etiology. Although most immune-related adverse events (irAEs) observed with immunomodulatory agents have been mild and self-limiting, such events should be recognized early and treated promptly to avoid potential major complications (Di Giacomo *et al*., 2010). Discontinuation of atezolizumab may not have an immediate therapeutic effect, and there is no available antidote for atezolizumab. In severe cases, immune-related toxicities may be acutely managed with topical corticosteroids, systemic corticosteroids, or other immunosuppressive agents. The investigator should consider the benefit-risk balance prior to further administration of atezolizumab.

For detailed information regarding management of adverse events associated with atezolizumab, please refer to the most current version of the Atezolizumab Investigator's Brochure and the FDA product label.

The primary approach to grade 1 to 2 irAEs is supportive and symptomatic care with continued treatment with atezolizumab; for higher-grade irAEs, atezolizumab should be withheld and oral and/or parenteral steroids administered. Recurrent grade 2 irAEs may also mandate withholding atezolizumab or the use of steroids. Assessment of the benefit-risk balance should be made by the investigator, with consideration of the totality of information as it pertains to the nature of the toxicity and the degree of clinical benefit a given patient may be experiencing prior to further administration of atezolizumab. Atezolizumab should be permanently discontinued in patients with life-threatening irAEs.

Patients should be assessed clinically (including review of laboratory values) for toxicity prior to, during, and after each infusion. If unmanageable toxicity due to atezolizumab occurs at any time during the study, treatment with atezolizumab should be discontinued.

Systemic Immune Activation

Systemic immune activation is a rare condition characterized by an excessive immune response. Given the mechanism of action of atezolizumab, systemic immune activation is considered a potential risk when given in combination with other immuneomodulating agents. Systemic immune activation should be included in the differential diagnosis for patients who, in the absence of an alternative etiology, develop a sepsis-like syndrome after administration of atezolizumab, and the initial evaluation should include the following:

- CBC with peripheral smear
- PT, PTT, fibrinogen, and D-dimer
- Ferritin
- Triglycerides
- AST, ALT, and total bilirubin
- LDH
- Complete neurologic and abdominal examination (assess for hepatosplenomegaly)

If systemic immune activation is still suspected after the initial evaluation, contact the Principal Investigator for additional recommendations.

6.2.2 Management of Specific AEs

Management of certain AEs of concern, including immune-related pneumonitis, hepatitis, colitis, endocrinopathies, pancreatitis, neuropathies, meningoencephalitis, and potential ocular toxicities are presented in the Atezolizumab Investigator's Brochure. See Section 5.1.2 and the **AE Management and Dose Interruption Guidelines for Specific Toxicities** table below for guidelines for the management of Infusion Related Reactions and Anaphylaxis.

For recommendations to hold atezolizumab and begin corticosteroid treatment, use the following guidance for tapering the corticosteroid and resuming atezolizumab therapy after resolution of the event:

- Corticosteroid taper: Corticosteroids must be tapered over ≥1 month to ≤10 mg/day oral prednisone or equivalent before atezolizumab can be resumed. A shorter steroid taper may be allowed if appropriate, after discussion with the medical oncology co-chair.
- Atezolizumab may be held for a period of time beyond 12 weeks to allow for corticosteroids to be reduced to ≤10 mg/day oral prednisone or equivalent.

Pulmonary Events
Dyspnea, cough, fatigue, hypoxia, pneumonitis, and pulmonary infiltrates have been associated with the administration of atezolizumab. Patients will be assessed for pulmonary signs and symptoms throughout the study.

All pulmonary events should be thoroughly evaluated for other commonly reported etiologies such as pneumonia or other infection, lymphangitic carcinomatosis, pulmonary embolism, heart failure, chronic obstructive pulmonary disease, or pulmonary hypertension. Management guidelines for pulmonary events are provided in the **AE Management and Dose Interruption Guidelines for Specific Toxicities** table below.

Endocrine Disorders
Patients experiencing one or more unexplained AEs possibly indicative of endocrine dysfunction (including fatigue, myalgias, impotence, mental status changes, or constipation) should be investigated for the presence of thyroid, pituitary, or adrenal endocrinopathies. The patient should be referred to an endocrinologist if an endocrinopathy is suspected. Thyroid-stimulating hormone (TSH) and free T3 and T4 levels should be measured to determine whether thyroid abnormalities are present. TSH, prolactin, and a morning cortisol level will help to differentiate primary adrenal insufficiency from primary pituitary insufficiency.

Dose management guidelines for hyperthyroidism, hypothyroidism, symptomatic adrenal insufficiency, and hyperglycemia are described in the **AE Management and Dose Interruption Guidelines for Specific Toxicities** table below.

Meningoencephalitis

Immune-related meningoencephalitis is an identified risk associated with the administration of atezolizumab. Immune-related meningoencephalitis should be suspected in any patient presenting with signs or symptoms suggestive of meningitis or encephalitis, including, but not limited to, headache, neck pain, confusion, seizure, motor or sensory dysfunction, and altered or depressed level of consciousness. Encephalopathy from metabolic or electrolyte imbalances needs to be distinguished from potential meningoencephalitis resulting from infection (bacterial, viral, or fungal) or progression of malignancy, or secondary to a paraneoplastic process.

All patients being considered for meningoencephalitis should be urgently evaluated with a CT scan and/or MRI scan of the brain to evaluate for metastasis, inflammation, or edema. If deemed safe by the treating physician, a lumbar puncture should be performed and a neurologist should be consulted.

Patients with signs and symptoms of meningoencephalitis, in the absence of an identified alternate etiology, should be treated according to the guidelines in the **AE Management and Dose Interruption Guidelines for Specific Toxicities** table below.

Neurologic disorders

Myasthenia gravis and Guillain-Barré syndrome have been observed with single-agent atezolizumab. Patients may present with signs and symptoms of sensory and/or motor neuropathy. Diagnostic work-up is essential for an accurate characterization to differentiate between alternative etiologies. Management guidelines for neurologic disorders are provided in the table below.

For recommendations to hold atezolizumab and begin corticosteroid treatment, use the following guidance for tapering the corticosteroid and resuming atezolizumab therapy after resolution of the event:

- Corticosteroids must be tapered over ≥1 month to ≤10 mg/day oral prednisone or equivalent before atezolizumab can be resumed.
- Atezolizumab may be held for a period of time beyond 12 weeks to allow for corticosteroids to be reduced to ≤10 mg/day oral prednisone or equivalent.

Renal Disorders

It is recommended that TECENTRIQ®(atezolizumab) should be withheld for moderate (Grade 2) immune-related nephritis and permanently discontinued for severe nephritis (Grade 3 and 4). Please refer patient to renal specialist and consider renal biopsy and supportive measures as indicated. Corticosteroids and/or additional immunosuppressive agents should be administered as clinically indicated.

AE Management and Dose Interruption Guidelines for Specific Toxicities		
**Toxicity**	**Severity/ Duration**	**Management**
Abdominal pain	Acute abdominal pain	Symptoms of abdominal pain associated with elevations of amylase and lipase, suggestive of pancreatitis, have been associated with administration of other immunomodulatory agents. The differential diagnosis of acute abdominal pain should include pancreatitis. Appropriate workup should include an evaluation for obstruction, as well as serum amylase and lipase tests. See the guidelines for "Amylase and/or lipase increase" and "Immune-related pancreatitis" elsewhere in this table, as needed.<br><br>Right upper-quadrant abdominal pain and/or unexplained nausea or vomiting should be evaluated for potential hepatotoxicity (see the "Hepatotoxicity" guideline elsewhere in this table).
Adrenal insufficiency	Grade 2+ (symptomatic)	Hold atezolizumab.<br><br>Refer patient to endocrinologist.<br><br>Perform appropriate imaging.<br><br>Initiate treatment with 1–2 mg/kg/day intravenous methylprednisolone or equivalent and convert to 1–2 mg/kg/day oral prednisone or equivalent upon improvement.<br><br>If event resolves to grade 1 or better and patient is stable on replacement therapy (if required) within 12 weeks, taper corticosteroids and resume atezolizumab according to the guidelines above.<br><br>Permanently discontinue atezolizumab if event does not resolve to grade 1 or better or patient is not stable on replacement therapy within 12 weeks.
Amylase and/or lipase increased	Grade 1	Continue atezolizumab.<br><br>Monitor amylase and lipase prior to dosing.

AE Management and Dose Interruption Guidelines for Specific Toxicities		
**Toxicity**	**Severity/ Duration**	**Management**
	Grade 2	Continue atezolizumab.<br><br>Monitor amylase and lipase weekly.<br><br>For prolonged elevation (*e.g.*, >3 weeks), consider treatment with 10 mg/day oral prednisone or equivalent.
	Grade 3 or 4	Hold atezolizumab.<br><br>Consider referral to gastrointestinal (GI) specialist.<br><br>Monitor amylase and lipase every other day.<br><br>If no improvement, consider treatment with 1–2 mg/kg/day oral prednisone or equivalent.<br><br>If event resolves to grade 1 or better within 12 weeks, taper corticosteroids and resume atezolizumab according to the guidelines above.<br><br>Permanently discontinue atezolizumab if event does not resolve to grade 1 or better within 12 weeks.<br><br>For recurrent events, permanently discontinue atezolizumab.
Dermatologic toxicity/rash (*e.g.*, maculopapular or purpura)	Grade 1	Continue atezolizumab.<br><br>Consider topical steroids and/or other symptomatic therapy (*e.g.*, antihistamines).
	Grade 2	Continue atezolizumab. Consider dermatologist referral.<br><br>Administer topical corticosteroids.<br><br>Consider higher potency topical corticosteroids if event does not improve.

AE Management and Dose Interruption Guidelines for Specific Toxicities		
**Toxicity**	**Severity/ Duration**	**Management**
	Grade 3	Hold atezolizumab.<br><br>Refer patient to dermatologist. Administer oral prednisone 10 mg or equivalent. If the event does not improve within 48–72 hours, increase dose to1–2 mg/kg/day or equivalent.<br>Restart atezolizumab if event resolves to grade 1 or better within 12 weeks.<br><br>Permanently discontinue atezolizumab if event does not resolve to grade 1 or better within 12 weeks.
	Grade 4	Permanently discontinue atezolizumab. Patient may not resume treatment, regardless of benefit. Otherwise, manage as above.
	Persistent and/or severe rash or pruritus, any grade	A dermatologist should evaluate the event. A biopsy should be performed unless contraindicated.
Diarrhea or colitis	Any grade	Patients should be advised to inform the investigator if any diarrhea occurs, even if it is mild.<br><br>All events of diarrhea or colitis should be thoroughly evaluated for other more common etiologies. For events of significant duration or magnitude or associated with signs of systemic inflammation or acute-phase reactants (*e.g.*, increased CRP, platelet count, or bandemia): Perform sigmoidoscopy (or colonoscopy, if appropriate) with colonic biopsy, with three to five specimens for standard paraffin block to check for inflammation and lymphocytic infiltrates to confirm colitis diagnosis.
	Grade 1	Continue atezolizumab.<br><br>Initiate symptomatic treatment.<br><br>Endoscopy is recommended if symptoms persist for >7 days.<br><br>Monitor closely.

AE Management and Dose Interruption Guidelines for Specific Toxicities		
**Toxicity**	**Severity/ Duration**	**Management**
	Grade 2	Hold atezolizumab.<br><br>Initiate symptomatic treatment.<br><br>Patient referral to GI specialist is recommended.<br><br>For recurrent events or events that persist >5 days, initiate treatment with 1–2 mg/kg/day oral prednisone or equivalent.<br><br>If event resolves to grade 1 or better within 12 weeks, taper corticosteroids and resume atezolizumab according to the guidelines above.<br><br>Permanently discontinue atezolizumab if event does not resolve to grade 1 or better within 12 weeks. Resumption of atezolizumab may be considered, after consultation with the trial PI, in patients who are deriving benefit and have fully recovered from the immune-related event.
	Grade 3	Hold atezolizumab.<br><br>Refer patient to GI specialist for evaluation and confirmatory biopsy.<br><br>Initiate treatment with 1–2 mg/kg/day intravenous methylprednisolone or equivalent and convert to 1–2 mg/kg/day oral prednisone or equivalent upon improvement.<br><br>If event resolves to grade 1 or better within 12 weeks, taper corticosteroids and resume atezolizumab according to the guidelines above.<br><br>Permanently discontinue atezolizumab if event does not resolve to Grade 1 or better within 12 weeks. Resumption of atezolizumab may be considered, after consultation with the trial PI, in patients who are deriving benefit and have fully recovered from the immune-related event.

AE Management and Dose Interruption Guidelines for Specific Toxicities		
**Toxicity**	**Severity/ Duration**	**Management**
	Grade 4	Permanently discontinue atezolizumab. Patient may not resume treatment, regardless of benefit.<br><br>Refer patient to GI specialist for evaluation and confirmation biopsy.<br><br>Initiate treatment with 1–2 mg/kg/day intravenous methylprednisolone or equivalent and convert to 1–2 mg/kg/day oral prednisone or equivalent upon improvement.<br><br>If event does not improve within 48 hours after initiating corticosteroids, consider adding an immunosuppressive agent.<br><br>If event resolves to grade 1 or better, taper corticosteroids over ≥1 month. Resumption of atezolizumab may be considered in patients who are deriving benefit and have fully recovered from the immune-related event.
Hepatotoxicity	Right upper-quadrant abdominal pain and/or unexplained nausea or vomiting	Risk of immune-mediated hepatitis. LFTs should be performed immediately, and LFTs should be reviewed before administration of the next dose of study drug. For patients with elevated LFTs, concurrent medication, viral hepatitis, and toxic or neoplastic etiologies should be considered and addressed, as appropriate.<br><br>Symptoms of abdominal pain associated with elevations of amylase and lipase, suggestive of pancreatitis, have been associated with the administration of atezolizumab. The differential diagnosis of acute abdominal pain should also include pancreatitis, as described below.
	Grade 1 hepatic event	Continue atezolizumab.<br><br>Monitor LFTs until values resolve to within normal limits.
	Grade 2 hepatic event, ≤5 days	Continue atezolizumab.<br><br>Monitor LFTs more frequently until values resolve to baseline values.

AE Management and Dose Interruption Guidelines for Specific Toxicities		
**Toxicity**	**Severity/ Duration**	**Management**
	Grade 2 hepatic event, >5 days	Hold atezolizumab.<br><br>Initiate treatment with 1–2 mg/kg/day oral prednisone or equivalent.<br><br>If event resolves to grade 1 or better within 12 weeks, taper corticosteroids and resume atezolizumab according to the guidelines above.<br><br>Permanently discontinue atezolizumab if event does not resolve to Grade 1 or better within 12 weeks.
	Grade 3 or 4 hepatic event	Permanently discontinue atezolizumab.<br><br>Consider patient referral to GI specialist for evaluation and liver biopsy to establish etiology of hepatic injury.<br><br>Initiate treatment with 1–2 mg/kg/day oral prednisone or equivalent.<br><br>If event does not improve within 48 hours after initiating corticosteroids, consider adding an immunosuppressive agent.<br><br>If event resolves to grade 1 or better, taper corticosteroids over ≥1 month.
Hyperglycemia	Grade 1 or 2	Continue atezolizumab.<br><br>Initiate treatment with insulin if needed.<br><br>Monitor for glucose control.
	Grade 3 or 4	Hold atezolizumab.<br><br>Initiate treatment with insulin.<br><br>Monitor for glucose control.<br><br>Resume atezolizumab when symptoms resolve and glucose levels are stable.

AE Management and Dose Interruption Guidelines for Specific Toxicities		
**Toxicity**	**Severity/ Duration**	**Management**
Hyperthyroidism	Grade 1 (asymptomatic)	**TSH ≥ 0.1 mU/L and < 0.5 mU/L:** Continue atezolizumab. Monitor TSH every 4 weeks.<br><br>**TSH < 0.1 mU/L:** Follow guidelines for symptomatic hyperthyroidism.
	Grade 2+ (symptomatic)	Hold atezolizumab.<br><br>Initiate treatment with anti-thyroid drug such as methimazole or carbimazole as needed.<br><br>Consider patient referral to endocrinologist.<br><br>Resume atezolizumab when symptoms are controlled and thyroid function is improving.<br><br>Permanently discontinue atezolizumab for life-threatening immune-related hyperthyroidism.
Hypothyroidism	Grade 1 (asymptomatic)	Continue atezolizumab.<br><br>Start thyroid-replacement hormone.<br><br>Monitor TSH weekly.
	Grades 2+ (symptomatic)	Hold atezolizumab.<br><br>Start thyroid-replacement hormone. Consider referral to an endocrinologist.<br><br>Monitor TSH weekly.<br><br>Restart atezolizumab when symptoms are controlled and thyroid function is improving.

AE Management and Dose Interruption Guidelines for Specific Toxicities		
**Toxicity**	**Severity/ Duration**	**Management**
Meningo-encephalitis, immune-related<br><br>(signs and symptoms in absence of an identified alternate etiology)	All grades	Permanently discontinue atezolizumab. <u>Patient may not resume treatment, regardless of benefit.</u><br><br>Refer patient to neurologist.<br><br>Initiate treatment with 1–2 mg/kg/day IV methylprednisolone or equivalent and convert to 1–2 mg/kg/day oral prednisone or equivalent upon improvement.<br><br>If event resolves to grade 1 or better, taper corticosteroids over ≥1 month.<br><br>If event does not improve within 48 hours after initiating corticosteroids, consider adding an immunosuppressive agent.
Myasthenia gravis and Guillain-Barré syndrome	All grades	Permanently discontinue atezolizumab. <u>Patient may not resume treatment, regardless of benefit.</u><br><br>Refer patient to neurologist.<br><br>Initiate treatment as per institutional guidelines.<br><br>Consider initiation of 1–2 mg/kg/day oral or IV prednisone or equivalent.
Myocarditis	All grades	Please see table 48 in section 6.7. in the investigator's brochure.
Neuropathy, immune-related<br><br>(sensory and/or motor)	Grade 1	Continue atezolizumab.<br><br>Evaluate for alternative etiologies.
	Grade 2	Hold atezolizumab.<br><br>Evaluate for alternative etiologies.<br><br>Initiate treatment as per institutional guidelines.<br><br>Resume atezolizumab if event resolves to grade 1 or better within 12 weeks.<br><br>Permanently discontinue atezolizumab if event does not resolve to grade 1 or better within 12 weeks.

AE Management and Dose Interruption Guidelines for Specific Toxicities		
**Toxicity**	**Severity/ Duration**	**Management**
	Grade 3 or 4	Permanently discontinue atezolizumab.<br><br>Initiate treatment as per institutional guidelines.
Ocular event (*e.g.*, uveitis, retinal events)	Grade 1	Continue atezolizumab.<br><br>Patient referral to ophthalmologist is strongly recommended.<br><br>Initiate treatment with topical corticosteroid eye drops and topical immunosuppressive therapy.<br><br>If symptoms persist, treat as a grade 2 event.
	Grade 2	Withhold atezolizumab.<br><br>Patient referral to ophthalmologist is strongly recommended.<br><br>Initiate treatment with topical corticosteroid eye drops and topical immunosuppressive therapy.<br><br>If event resolves to grade 1 or better within 12 weeks, taper corticosteroids and resume atezolizumab according to the guidelines above.<br><br>Permanently discontinue atezolizumab if event does not resolve to grade 1 or better within 12 weeks.
	Grade 3 or 4	Permanently discontinue atezolizumab.<br><br>Refer patient to ophthalmologist.<br><br>Initiate treatment with 1–2 mg/kg/day oral prednisone or equivalent.<br><br>If event resolves to grade 1 or better, taper corticosteroids over ≥1 month. For grade 3 AEs, patient may only resume treatment after consultation with the trial PI; for grade 4, patient cannot resume treatment, regardless of benefit.

AE Management and Dose Interruption Guidelines for Specific Toxicities		
**Toxicity**	**Severity/ Duration**	**Management**
Pancreatitis, immune related<br><br>See Amylase and/or lipase increased section above	Grade 2 (with radiographic findings) or 3	Hold atezolizumab.<br><br>Refer patient to GI specialist.<br><br>Initiate treatment with 1–2 mg/kg/day intravenous methylprednisolone or equivalent and convert to 1–2 mg/kg/day oral prednisone or equivalent upon improvement.<br><br>If event resolves to grade 1 or better within 12 weeks, taper corticosteroids and resume atezolizumab according to the guidelines above.<br><br>Permanently discontinue atezolizumab if event does not resolve to grade 1 or better within 12 weeks. Patient may only resume treatment after consultation with the trial PI.<br><br>For recurrent events, permanently discontinue atezolizumab. <u>Patient may not resume treatment, regardless of benefit.</u>
	Grade 4	Permanently discontinue atezolizumab. <u>Patient may not resume treatment, regardless of benefit.</u><br><br>Refer patient to GI specialist.<br><br>Initiate treatment with 1–2 mg/kg/day intravenous methylprednisolone or equivalent and convert to 1–2 mg/kg/day oral prednisone or equivalent upon improvement.<br><br>If event does not improve within 48 hours after initiating corticosteroids, consider adding an immunosuppressive agent.<br><br>If event resolves to grade 1 or better, taper corticosteroids over ≥1 month.
Pulmonary toxicity	All pulmonary events	Evaluate thoroughly for other commonly reported etiologies such as pneumonia/infection, lymphangitic carcinomatosis, pulmonary embolism, heart failure, chronic obstructive pulmonary disease (COPD), or pulmonary hypertension.

AE Management and Dose Interruption Guidelines for Specific Toxicities		
**Toxicity**	**Severity/ Duration**	**Management**
	Grade 1	Continue atezolizumab and monitor closely.<br><br>Re-evaluate on serial imaging.<br><br>Consider patient referral to a pulmonary specialist.<br><br>For recurrent pneumonitis, treat as a grade 3 or 4 event.
	Grade 2	Hold atezolizumab.<br><br>Refer patient to pulmonary and infectious disease specialists and consider bronchoscopy or bronchoscopic alveolar lavage (BAL).<br><br>Initiate treatment with 1–2 mg/kg/day oral prednisone or equivalent.<br><br>If event resolves to grade 1 or better within 12 weeks, taper corticosteroids and resume atezolizumab according to the guidelines above.<br><br>Permanently discontinue atezolizumab if event does not resolve to grade 1 or better within 12 weeks.<br><br>For recurrent events, treat as a Grade 3 or 4 event.
	Grade 3 or 4	Permanently discontinue atezolizumab.<br><br>Bronchoscopy or BAL is recommended.<br><br>Initiate treatment with 1–2 mg/kg/day oral prednisone or equivalent.<br><br>If event does not improve within 48 hours after initiating corticosteroids, consider adding an immunosuppressive agent.<br><br>If event resolves to grade 1 or better, taper corticosteroids over ≥1 month. For grade 3 AEs, patient may only resume treatment after consultation with the trial PI; for grade 4, patient cannot resume treatment, regardless of benefit.

6.3 Radiation Therapy Dose Delivery Modifications

6.3.1 Radiation therapy may only be suspended secondary to a significant adverse event at the joint discretion of the patient's treating physicians and the study chair (face sheet). The study chair must be notified in writing (e.g., email) of dose modifications or treatment modality modifications for external beam radiation therapy or for brachytherapy.

6.3.2 Because it has been observed that hemoglobin levels below 10-12 g/dL during radiation therapy are associated with decreased local control, blood transfusions should be offered to patients and used to treat patients at the discretion of treating physicians prior to or during radiotherapy per institutional protocol. There should be no radiation therapy treatment delays due to a low hemoglobin levels. Published results evaluating epoeitin alpha (Procrit, Epogen) and radiochemotherapy in cervical cancer have indicated that epoeitin alpha may be associated with an increased risk for thromboembolism, and therefore, may NOT be used in this study.

6.3.3 Hematologic Adverse Events: Hematologic toxicities are seen infrequently unless pelvic radiation occurs with chemotherapy. A complete blood count should be obtained weekly during radiochemotherapy. If the absolute neutrophil count falls to less than 500 / mm^3 or platelet count falls to less than 25,000 / mm^3, radiation therapy may be temporarily withheld to allow recovery above these levels at the discretion of the treating physician.

6.3.4 Gastrointestinal Adverse Events: Nausea and vomiting are rather unusual after pelvic radiation. Antiemetics may be given when symptoms occur or may be given prophylactically prior to treatment per institutional protocol. Intractable nausea or vomiting is rarely seen with pelvic radiation alone and is usually the result of another process, i.e. bowel obstruction. Increased bowel activity with diarrhea usually can be controlled with low fiber, low fat, bland diets and antidiarrheal medications. Should gastrointestinal toxicity become severe, hospitalization may be required at which time the treatment may be interrupted temporarily until the patient's condition improves.

6.3.5 Genitourinary Adverse Events: Acute toxicity of the urinary tract is manifested by cystitis. Maintaining high fluid intake is important. Bladder antispasmodics, analgesics and antibiotics are recommended per institutional protocol. Hematuria is not usually seen with acute cystitis and suggests bladder invasion by tumor. Acute vulvovaginitis is seen when the pelvic fields extend inferiorly to include the vulvoperineal area. Treatment of acute vulvoperineal reaction may require warm saline soaks, wearing loose clothing and keeping the area dry using a hair dryer. Topical steroids, antibiotics, creams, and treatment interruption may be necessary per institutional protocol.

6.3.6 Cutaneous Skin Toxicity Adverse Events: With the use of megavoltage external beam radiation therapy, skin reactions in the treatment field are infrequent but are more likely to develop if sites such as the inguinal area, vulva, and the perineum are extensively within the radiation fields. During radiation therapy, mild irritation and redness of the skin may occur within the radiation fields. Some patients may experience more intense skin reaction such as dry or moist desquamation depending upon the energy of the megavoltage beam, number of fields used per day, need to cover distal vagina and therefore flashing perineum and use of chemotherapy. Hair loss in the pubic area may occur which can be permanent. Late subcutaneous fibrosis, telangiectasia, and skin atrophy are uncommon sequelae. Almost all patients with vulvar/perineal skin in the treatment fields may expect to develop acute moist desquamation during the course of external beam radiation therapy.

Acute skin reactions may be treated according to institutional preferences. Aquaphor or steroid creams may be used for CTCAE v4 grade 1-2 reactions and it is not necessary to interrupt the radiation therapy. For CTCAE v4 grade 3-4 skin reactions when generalized macular, papular or vesicular eruptions have developed, or there is generalized exfoliative or ulcerative dermatitis in the treatment fields, radiation therapy may be interrupted, but this should be avoided. Treatment may require symptomatic management of pain, use of warm saline soaks, vitamin A&D ointment, wearing loose clothing, and keeping the area dry according to institutional protocol. Radiation therapy should be resumed as soon as the skin reactions have improved. The principal investigator should be notified within 96 hours of any grade 4 toxicity.

7. ADVERSE EVENTS REPORTING REQUIREMENTS

7.1 Protocol Agents

Investigational Agents

The investigational agent administered in NRG-*GY017, is Atezolizumab*, which is being made available under an IND sponsored by CTEP, DCTD, NCI. For patients receiving *atezolizumab,* determination of whether an adverse event meets expedited reporting criteria, see the reporting table in section 7.6 of the protocol.

Commercial Agents

The commercial agent in NRG-GY017 is *Cisplatin.* In this study the commercial agent is used in combination with investigational agent; therefore, after receiving cisplatin, the expedited adverse event reporting criteria for an investigational agent/therapy apply; see reporting table in section 7.6 of the protocol.

7.2 Adverse Events and Serious Adverse Events

7.2.1

This study will utilize the NCI Common Terminology Criteria for Adverse Events (CTCAE) version 5.0 for reporting of adverse events (AEs), located on the CTEP web site, http://ctep.cancer.gov/protocolDevelopment/electronic_applications/ctc.htm. All appropriate treatment areas should have access to a copy of the CTCAE version 5.0

7.2.2 Definition of an Adverse Event (AE)

Any untoward medical occurrence associated with the use of a drug in humans, whether or not considered drug related. Therefore, an AE can be any unfavorable and unintended sign (including an abnormal laboratory finding), symptom, or disease temporally associated with the use of a medicinal (investigational) product, whether or not considered related to the medicinal (investigational) product (attribution of unrelated, unlikely, possible, probable, or definite). (International Conference on Harmonization [ICH], E2A, E6).

For multi-modality trials, adverse event reporting encompasses all aspects of protocol treatment including radiation therapy, surgery, device, and drug.

Due to the risk of intrauterine exposure of a fetus to potentially teratogenic agents, the pregnancy of a study participant must be reported via CTEP-AERS in an expedited manner.

7.3 Comprehensive Adverse Events and Potential Risks (CAEPR) List

7.3.1 Frequency is provided based on 3097 patients.

CAEPR for Atezolizumab (MPDL3280A, NSC 783608)

The Comprehensive Adverse Events and Potential Risks list (CAEPR) provides a single list of reported and/or potential adverse events (AE) associated with an agent using a uniform presentation of events by body system. In addition to the comprehensive list, a subset, the Specific Protocol Exceptions to Expedited Reporting (SPEER), appears in a separate column and is identified with bold and italicized text. This subset of AEs (SPEER) is a list of events that are protocol specific exceptions to expedited reporting to NCI (except as noted below). Refer to the 'CTEP, NCI Guidelines: Adverse Event Reporting Requirements' http://ctep.cancer.gov/protocolDevelopment/electronic_applications/docs/aeguidelines.pdf for further clarification. *Frequency is provided based on 3097 patients.* Below is the CAEPR for Atezolizumab (MPDL3280A).

NOTE: Report AEs on the SPEER **ONLY IF** they exceed the grade noted in parentheses next to the AE in the SPEER. If this CAEPR is part of a combination protocol using multiple investigational agents and has an AE listed on different SPEERs, use the lower of the grades to determine if expedited reporting is required.

Version 2.2, July 23, 2018[1]

Adverse Events with Possible Relationship to Atezolizumab (MPDL3280A) (CTCAE 5.0 Term) [n= 3097]			Specific Protocol Exceptions to Expedited Reporting (SPEER)
**Likely (>20%)**	**Less Likely (<=20%)**	**Rare but Serious (<3%)**	
BLOOD AND LYMPHATIC SYSTEM DISORDERS			
	Anemia		
CARDIAC DISORDERS			

Adverse Events with Possible Relationship to Atezolizumab (MPDL3280A) (CTCAE 5.0 Term) [n= 3097]			Specific Protocol Exceptions to Expedited Reporting (SPEER)
**Likely (>20%)**	**Less Likely (<=20%)**	**Rare but Serious (<3%)**	
		Heart failure[2]	
		Myocarditis[2]	
		Pericardial effusion[2]	
		Pericardial tamponade[2]	
		Pericarditis[2]	
ENDOCRINE DISORDERS			
		Adrenal insufficiency[2]	
		Endocrine disorders - Other (diabetes)[2]	
	Hyperthyroidism[2]		
		Hypophysitis[2]	
	Hypothyroidism[2]		
EYE DISORDERS			
		Eye disorders - Other (ocular inflammatory toxicity)[2]	
		Uveitis[2]	
GASTROINTESTINAL DISORDERS			
	Abdominal pain		***Abdominal pain (Gr 2)***
		Colitis[2]	
	Diarrhea		***Diarrhea (Gr 2)***
	Dysphagia		
	Nausea		***Nausea (Gr 2)***
		Pancreatitis[2]	
	Vomiting		***Vomiting (Gr 2)***
GENERAL DISORDERS AND ADMINISTRATION SITE CONDITIONS			
Fatigue			***Fatigue (Gr 2)***
	Fever[3]		
	Flu like symptoms[3]		
HEPATOBILIARY DISORDERS			
		Hepatic failure[2]	
		Hepatobiliary disorders - Other (hepatitis)[2]	
IMMUNE SYSTEM DISORDERS			
	Allergic reaction[3]		
		Anaphylaxis[3]	
		Cytokine release syndrome[3]	
		Immune system disorders - Other (systemic immune activation)[2]	
INFECTIONS AND INFESTATIONS			
Infection[4]			
INJURY, POISONING AND PROCEDURAL COMPLICATIONS			
	Infusion related reaction[3]		
INVESTIGATIONS			
	Alanine aminotransferase increased[2]		
	Alkaline phosphatase increased[2]		
	Aspartate aminotransferase increased[2]		
	Blood bilirubin increased[2]		

Adverse Events with Possible Relationship to Atezolizumab (MPDL3280A) (CTCAE 5.0 Term) [n= 3097]			Specific Protocol Exceptions to Expedited Reporting (SPEER)
**Likely (>20%)**	**Less Likely (<=20%)**	**Rare but Serious (<3%)**	
	GGT increased[2]		
	Lipase increased*		
		Platelet count decreased	
	Serum amylase increased*		
METABOLISM AND NUTRITION DISORDERS			
	Anorexia		*Anorexia (Gr 2)*
		Hyperglycemia[2]	
	Hypokalemia		
	Hyponatremia		
MUSCULOSKELETAL AND CONNECTIVE TISSUE DISORDERS			
	Arthralgia[2]		
	Back pain		
		Generalized muscle weakness	
	Myalgia		
		Myositis[2]	
NERVOUS SYSTEM DISORDERS			
		Ataxia[2]	
		Encephalopathy[2]	
		Nervous system disorders - Other (encephalitis non-infective)[2]	
		Guillain-Barre syndrome[2]	
		Nervous system disorders - Other (meningitis non-infective)[2]	
		Myasthenia gravis[2]	
		Paresthesia[2]	
		Peripheral motor neuropathy[2]	
		Peripheral sensory neuropathy[2]	
RENAL AND URINARY DISORDERS			
		Renal and urinary disorders - Other (nephritis)[2]	
RESPIRATORY, THORACIC AND MEDIASTINAL DISORDERS			
	Cough		*Cough (Gr 2)*
	Dyspnea		
	Hypoxia		
	Nasal congestion		*Nasal congestion (Gr 2)*
		Pleural effusion[2]	
		Pneumonitis[2]	
SKIN AND SUBCUTANEOUS TISSUE DISORDERS			
		Bullous dermatitis[2]	
	Pruritus		
	Rash acneiform		
	Rash maculo-papular		
	Skin and subcutaneous tissue disorders - Other (lichen planus)[2]		

*Denotes adverse events that are <3%.

[1]This table will be updated as the toxicity profile of the agent is revised. Updates will be distributed to all Principal Investigators at the time of revision. The current version can be obtained by contacting PIO@CTEP.NCI.NIH.GOV. Your name, the name of the investigator, the protocol and the agent should be included in the e-mail.

[2]Atezolizumab, being a member of a class of agents involved in the inhibition of "immune checkpoints," may result in severe and possibly fatal immune-mediated adverse events probably due to T-cell activation and proliferation. Immune-mediated adverse reactions have been reported in patients receiving atezolizumab. Adverse events potentially related to atezolizumab may be manifestations of immune-mediated adverse events. In clinical trials, most immune-mediated adverse reactions were reversible and managed with interruptions of atezolizumab, administration of corticosteroids and supportive care.

[3]Infusion reactions, including high-grade hypersensitivity reactions, anaphylaxis, and cytokine release syndrome, which have been observed following administration of atezolizumab, may manifest as fever, chills, shakes, itching, rash, hypertension or hypotension, or difficulty breathing during and immediately after administration of atezolizumab.

[4]Infection includes all 75 sites of infection under the INFECTIONS AND INFESTATIONS SOC.

Adverse events reported on atezolizumab (MPDL3280A) trials, but for which there is insufficient evidence to suggest that there was a reasonable possibility that atezolizumab (MPDL3280A) caused the adverse event:

BLOOD AND LYMPHATIC SYSTEM DISORDERS - Blood and lymphatic system disorders - Other (pancytopenia); Febrile neutropenia
CARDIAC DISORDERS - Cardiac arrest; Ventricular tachycardia
GASTROINTESTINAL DISORDERS - Constipation; Dry mouth; Ileus
GENERAL DISORDERS AND ADMINISTRATION SITE CONDITIONS - Chills; Edema limbs; Malaise; Multi-organ failure
HEPATOBILIARY DISORDERS - Portal vein thrombosis
INVESTIGATIONS - Lymphocyte count decreased; Neutrophil count decreased; Weight loss; White blood cell decreased
METABOLISM AND NUTRITION DISORDERS - Hypophosphatemia; Tumor lysis syndrome
MUSCULOSKELETAL AND CONNECTIVE TISSUE DISORDERS - Bone pain; Muscle cramp: Pain in extremity
NERVOUS SYSTEM DISORDERS - Headache
PSYCHIATRIC DISORDERS - Confusion; Insomnia; Suicide attempt
RENAL AND URINARY DISORDERS - Acute kidney injury
REPRODUCTIVE SYSTEM AND BREAST DISORDERS - Breast pain
RESPIRATORY, THORACIC AND MEDIASTINAL DISORDERS - Bronchopulmonary hemorrhage; Pulmonary hypertension; Respiratory failure
SKIN AND SUBCUTANEOUS TISSUE DISORDERS - Alopecia; Dry skin[2]; Hyperhidrosis
VASCULAR DISORDERS - Hypertension; Hypotension; Thromboembolic event

Note: Atezolizumab (MPDL3280A) in combination with other agents could cause an exacerbation of any adverse event currently known to be caused by the other agent, or the combination may result in events never previously associated with either agent.

7.4 Adverse Events for Commercial Study Agents

Refer to the package insert for detailed pharmacologic and safety information

7.5 Adverse Event Characteristics

- **CTCAE term (AE description) and grade:** The descriptions and grading scales found in the revised NCI Common Terminology Criteria for Adverse Events (CTCAE) version 5.0 will be utilized for AE reporting. All appropriate treatment areas should have access to a copy of the CTCAE version 5.0. A copy of the CTCAE version 5.0 can be downloaded from the CTEP web site http://ctep.cancer.gov/protocolDevelopment/electronic_applications/ctc.htm.

- **For expedited reporting purposes only:**
  - AEs for the agent that are ***bold and italicized*** in the CAEPR (*i.e.*, those listed in the SPEER column, Section 7.3) should be reported through CTEP-AERS only if the grade is above the grade provided in the SPEER.

- **Attribution** of the AE:
  - Definite – The AE *is clearly related* to the study treatment.
  - Probable – The AE *is likely related* to the study treatment.
  - Possible – The AE *may be related* to the study treatment.
  - Unlikely – The AE *is doubtfully related* to the study treatment.
  - Unrelated – The AE *is clearly NOT related* to the study treatment

7.6 Expedited Reporting of Adverse Events

All serious adverse events that meet expedited reporting criteria defined in the reporting table below will be reported via the CTEP Adverse Event Reporting System, CTEP-AERS, accessed via the CTEP web site, https://eapps-ctep.nci.nih.gov/ctepaers/pages/task?rand=1390853489613

Submitting a report via CTEP-AERS serves as notification to NRG and satisfies NRG requirements for expedited adverse event reporting.

In the rare event when Internet connectivity is disrupted, a 24-hour notification must be made to CTEP for this study by telephone at 301-897-7497 and to the NRG Regulatory Affairs by phone at 215-854-0770. An electronic report must be submitted immediately upon re-establishment of the Internet connection.

7.6.1 Expedited Reporting Methods

- Per CTEP NCI Guidelines for Adverse Events Reporting Requirements, a CTEP-AERS 24-hour notification must be submitted within 24 hours of learning of the adverse event. Each CTEP-AERS 24-hour notification must be followed by a complete report within 5 days.
- Supporting source documentation is requested by the IND Sponsor for this study (CTEP/DCTD) and NRG as needed to complete adverse event review. Supporting source documentation should include the protocol number, patient ID number, and CTEP-AERS ticket number on each page, and fax supporting documentation to CTEP at 301-230-0159 and to NRG Regulatory Affairs at 215-854-0716

- A serious adverse event that meets expedited reporting criteria outlined in the AE Reporting Tables but is assessed by the CTEP-AERS as "an action *not* recommended" must still be reported to fulfill NRG safety reporting obligations. Sites must bypass the "NOT recommended" assessment; the CTEP-AERS allows submission of all reports regardless of the results of the assessment.

7.6.2 Expedited Reporting Requirements for Adverse Events

Phase 1 and Early Phase 2 Studies: Expedited Reporting Requirements for Adverse Events that Occur on Studies under a CTEP IND/IDE within 30 Days of the Last Administration of the Investigational Agent/Intervention [1, 2]

FDA REPORTING REQUIREMENTS FOR SERIOUS ADVERSE EVENTS (21 CFR Part 312)

NOTE: Investigators **<u>MUST</u>** immediately report to the sponsor (NCI) **<u>ANY</u>** Serious Adverse Events, whether or not they are considered related to the investigational agent(s)/intervention (21 CFR 312.64)

An adverse event is considered serious if it results in **<u>ANY</u>** of the following outcomes:

1) Death
2) A life-threatening adverse event
3) An adverse event that results in inpatient hospitalization or prolongation of existing hospitalization for ≥ 24 hours
4) A persistent or significant incapacity or substantial disruption of the ability to conduct normal life functions
5) A congenital anomaly/birth defect.
6) Important Medical Events (IME) that may not result in death, be life threatening, or require hospitalization may be considered serious when, based upon medical judgment, they may jeopardize the patient or subject and may require medical or surgical intervention to prevent one of the outcomes listed in this definition. (FDA, 21 CFR 312.32; ICH E2A and ICH E6).

<u>ALL</u> <u>SERIOUS</u> adverse events that meet the above criteria MUST be immediately reported to the NCI via CTEP-AERS within the timeframes detailed in the table below.

Hospitalization	Grade 1 and Grade 2 Timeframes	Grade 3-5 Timeframes
Resulting in Hospitalization ≥ 24 hrs	10 Calendar Days	24-Hour 5 Calendar Days
Not resulting in Hospitalization ≥ 24 hrs	Not required	

NOTE: Protocol specific exceptions to expedited reporting of serious adverse events are found in the Specific Protocol Exceptions to Expedited Reporting (SPEER) portion of the CAEPR

<u>Expedited AE reporting timelines are defined as:</u>

- "24-Hour; 5 Calendar Days" - The AE must initially be reported via CTEP-AERS within 24 hours of learning of the AE, followed by a complete expedited report within 5 calendar days of the initial 24-hour report.
- "10 Calendar Days" - A complete expedited report on the AE must be submitted within 10 calendar days of learning of the AE.

[1]Serious adverse events that occur **<u>more than</u>** 30 days after the last administration of investigational agent/intervention and have an attribution of possible, probable, or definite require reporting as follows:

Expedited 24-hour notification followed by complete report within 5 calendar days for:
- All Grade 3, 4, and Grade 5 AEs

Expedited 10 calendar day reports for:
- Grade 2 AEs resulting in hospitalization or prolongation of hospitalization

7.6.3 Adverse events of Special interest for Atezolizumab:

- Cases of potential drug-induced liver injury that include an elevated ALT or AST in combination with either an elevated bilirubin or clinical jaundice, as defined by Hy's Law and based on the following observations:
  - Treatment-emergent ALT or AST > 3 x ULN (or > 3 x baseline value in disease states where LFTs may be elevated at baseline) in combination with total bilirubin > 2 x ULN (of which ≥ 35% is direct bilirubin)
  - Treatment-emergent ALT or AST > 3 x ULN (or > 3 x baseline value in disease states where LFTs may be elevated at baseline) in combination with clinical jaundice
- Suspected transmission of an infectious agent by the study treatment, as defined below:
  - Any organism, virus, or infectious particle (e.g., prion protein transmitting transmissible spongiform encephalopathy), pathogenic or non-pathogenic, is considered an infectious agent. A transmission of an infectious agent may be suspected from clinical symptoms or laboratory findings that indicate an infection in a patient exposed to a medicinal product. This term applies only when a contamination of study treatment is suspected.
- Pneumonitis
- Colitis
- Endocrinopathies: diabetes mellitus, pancreatitis, adrenal insufficiency, hyperthyroidism, and hypophysitis
- Hepatitis, including AST or ALT > 10xULN
- Systemic lupus erythematosus
- Neurological disorders: Guillain-Barré syndrome, myasthenic syndrome or myasthenia gravis, and meningoencephalitis
- Events suggestive of hypersensitivity, infusion-related reactions, cytokine release syndrome, influenza-like illness, systemic inflammatory response syndrome, and systemic immune activation
- Nephritis
- Ocular toxicities (e.g., uveitis, retinitis, optic neuritis)
- Myositis
- Myopathies, including rhabdomyolysis
- Grade > 2 cardiac disorders (e.g., atrial fibrillation, myocarditis, pericarditis)
- Vasculitis
- Autoimmune hemolytic anemia
- Severe cutaneous reactions (e.g., Stevens-Johnson syndrome, dermatitis bullous, toxic epidermal necrolysis)

7.6.4 Reporting to the Site IRB/REB

Investigators will report serious adverse events to the local Institutional Review Board (IRB) or Research Ethics Board (REB) responsible for oversight of the patient according to institutional policy.

7.6.5 Secondary Malignancies

A secondary malignancy is a cancer caused by treatment for a previous malignancy (e.g., treatment with investigational agent/intervention, radiation or chemotherapy). A secondary malignancy is not considered a metastasis of the initial neoplasm.

CTEP requires all secondary malignancies that occur during or subsequent to treatment with an agent under an NCI IND/IDE be reported via CTEP-AERS. In addition, secondary malignancies following radiation therapy must be reported via CTEP-AERS. Three options are available to describe the event:

- Leukemia secondary to oncology chemotherapy (e.g., acute myelocytic leukemia [AML])
- Myelodysplastic syndrome (MDS)
- Treatment-related secondary malignancy

Any malignancy possibly related to cancer treatment (including AML/MDS) should also be reported via the routine reporting mechanisms outlined in each protocol.

Second Malignancy:

A second malignancy is one unrelated to the treatment of a prior malignancy (and is NOT a metastasis from the initial malignancy). Second malignancies require ONLY routine reporting via CDUS unless otherwise specified.

8. REGISTRATION AND STUDY ENTRY PROCEDURES

8.1 CTEP Registration Procedures

Food and Drug Administration (FDA) regulations and National Cancer Institute (NCI) policy require all individuals contributing to NCI-sponsored trials to register and to renew their registration annually. To register, all individuals must obtain a Cancer Therapy Evaluation Program (CTEP) Identity and Access Management (IAM) account (https://ctepcore.nci.nih.gov/iam). In addition, persons with a registration type of Investigator (IVR), Non-Physician Investigator (NPIVR), or Associate Plus (AP) (i.e., clinical site staff requiring write access to OPEN, RAVE, or TRIAD or acting as a primary site contact) must complete their annual registration using CTEP's web-based Registration and Credential Repository (RCR) (https://ctepcore.nci.nih.gov/rcr). Documentation requirements per registration type are outlined in the table below.

Documentation Required	IVR	NPIVR	AP	A
FDA Form 1572	✔	✔		

Financial Disclosure Form	✔	✔	✔	
NCI Biosketch (education, training, employment, license, and certification)	✔	✔	✔	
HSP/GCP training	✔	✔	✔	
Agent Shipment Form (if applicable)	✔			
CV (optional)	✔	✔	✔	

An active CTEP-IAM user account and appropriate RCR registration is required to access all CTEP and CTSU (Cancer Trials Support Unit) websites and applications. In addition, IVRs and NPIVRs must list all clinical practice sites and IRBs covering their practice sites on the FDA Form 1572 in RCR to allow the following:

- Added to a site roster
- Assigned the treating, credit, consenting, or drug shipment (IVR only) tasks in OPEN
- Act as the site-protocol PI on the IRB approval
- Assigned the Clinical Investigator (CI) role on the Delegation of Tasks Log (DTL).

Additional information can be found on the CTEP website at <https://ctep.cancer.gov/investigatorResources/default.htm >. For questions, please contact the RCR ***Help Desk*** by email at < RCRHelpDesk@nih.gov>.

8.2 CTSU Registration Procedures

This study is supported by the NCI Cancer Trials Support Unit (CTSU).

IRB Approval:

Each investigator or group of investigators at a clinical site must obtain IRB approval for this protocol and submit IRB approval and supporting documentation to the CTSU Regulatory Office before they can be approved to enroll patients. Assignment of site registration status in the CTSU Regulatory Support System (RSS) uses extensive data to make a determination of whether a site has fulfilled all regulatory criteria including but not limited to the following:

- An active Federal Wide Assurance (FWA) number
- An active roster affiliation with the Lead Network or a participating organization
- A valid IRB approval
- Compliance with all protocol specific requirements.

In addition, the site-protocol Principal Investigator (PI) must meet the following criteria:

- Active registration status
- The IRB number of the site IRB of record listed on their Form FDA 1572
- An active status on a participating roster at the registering site.

Sites participating on the NCI CIRB initiative that are approved by the CIRB for this study are not required to submit IRB approval documentation to the CTSU Regulatory Office. For sites using the CIRB, IRB approval information is received from the CIRB and applied to the RSS in an automated process. Signatory Institutions must submit a Study Specific Worksheet for Local Context (SSW) to the CIRB via IRB Manager to indicate their intent to open the study locally. The CIRB's approval of the SSW is then communicated to the CTSU Regulatory Office. In order for the SSW approval to be processed, the Signatory Institution must inform the CTSU which CIRB-approved institutions aligned with the Signatory Institution are participating in the study.

Downloading Site Registration Documents:

Site registration forms may be downloaded from the NRG-GY017 protocol page located on the CTSU members' website. Permission to view and download this protocol and its supporting documents is restricted and is based on person and site roster assignment housed in the CTSU RSS.

- Go to https://www.ctsu.org and log in to the members' area using your CTEP-IAM username and password
- Click on the Protocols tab in the upper left of your screen
- Either enter the protocol # NRG-GY017 in the search field at the top of the protocol tree, or
- Click on the By Lead Organization folder to expand
- Click on the NRG Oncology link to expand, then select trial protocol # NRG-GY017.
- Click on LPO Documents, select the Site Registration documents link, and download and complete the forms provided.

Requirements For NRG-GY017 Site Registration:

- IRB approval (For sites not participating via the NCI CIRB; local IRB documentation, an IRB-signed CTSU IRB Certification Form, Protocol of Human Subjects Assurance Identification/IRB Certification/Declaration of Exemption Form, or combination is accepted)

Submitting Regulatory Documents:

Submit required forms and documents to the CTSU Regulatory Office via the Regulatory Submission Portal, where they will be entered and tracked in the CTSU RSS.

Regulatory Submission Portal: www.ctsu.org (members' area) → Regulatory Tab →Regulatory Submission
When applicable, original documents should be mailed to:
CTSU Regulatory Office
1818 Market Street, Suite 3000
Philadelphia, PA 19103
Institutions with patients waiting that are unable to use the Portal should alert the CTSU Regulatory Office immediately at 1-866-651-2878 in order to receive further instruction and support.

Checking Your Site's Registration Status:

You can verify your site registration status on the members' section of the CTSU website.

- Go to https://www.ctsu.org and log in to the members' area using your CTEP-IAM username and password
- Click on the Regulatory tab
- Click on the Site Registration tab
- Enter your 5-character CTEP Institution Code and click on Go

Note: The status given only reflects compliance with IRB documentation and institutional compliance with protocol-specific requirements outlined by the Lead Network. It does not reflect compliance with protocol requirements for individuals participating on the protocol or the enrolling investigator's status with the NCI or their affiliated networks.

8.3 Patient Enrollment

Patient enrollment will be facilitated using the Oncology Patient Enrollment Network (OPEN). OPEN is a web-based registration system available on a 24/7 basis. To access OPEN, the site user must have an active CTEP-IAM account (check at < https://ctepcore.nci.nih.gov/iam >) and a 'Registrar' role on either the LPO or participating organization roster. Registrars must hold a minimum of an AP registration type. If a DTL is required for the study, the registrar(s) must also be assigned the OPEN Registrar task on the DTL.

All site staff will use OPEN to enroll patients to this study. It is integrated with the CTSU Enterprise System for regulatory and roster data and, upon enrollment, initializes the patient in the Rave database. OPEN can be accessed at https://open.ctsu.org or from the OPEN tab on the CTSU members' side of the website at https://www.ctsu.org. To assign an IVR or NPIVR as the treating, crediting, consenting, drug shipment (IVR only), or investigator receiving a transfer in OPEN, the IVR or NPIVR must list on their Form

FDA 1572 in RCR the IRB number used on the site's IRB approval. If a DTL is required for the study, the IVR or NPIVR must also be assigned the appropriate OPEN-related tasks on the DTL.

Prior to accessing OPEN, site staff should verify the following:

- All eligibility criteria have been met within the protocol stated timeframes.
- All patients have signed an appropriate consent form and HIPAA authorization form (if applicable).

Note: The OPEN system will provide the site with a printable confirmation of registration and treatment information. Please print this confirmation for your records.

Further instructional information is provided on the OPEN tab of the CTSU members' side of the CTSU website at https://www.ctsu.org or at https://open.ctsu.org. For any additional questions contact the CTSU Help Desk at 1-888-823-5923 or ctsucontact@westat.com.

8.4 RT-Specific Pre-Registration Requirements

For detailed information on the specific technology requirement required for this study, please refer to the table below and utilize the web link provided for detailed instructions. The check marks under the treatment modality columns indicate whether that specific credentialing requirement is required for this study. IROC-Houston will be the entity to notify your institution when all credentialing requirements have been met and the institution is RT credentialed to enter patients onto this study.
See the table above for the credentialing requirements of this study.

RT Credentialing Requirements	Web Link for Procedures and Instructions: http://irochouston.mdanderson.org	
	Treatment Modality	
	IMRT	**Key Information**
Facility Questionnaire	X	The IROC Houston electronic facility questionnaire (FQ) should be completed or updated with the most recent information about your institution. To access this FQ, email irochouston@mdanderson.org to receive your FQ link.
Credentialing Status Inquiry Form	X	To determine whether your institution needs to complete any further credentialing requirements, please complete the "Credentialing Status Inquiry Form" found under credentialing on the IROC Houston QA Center website (http://irochouston.mdanderson.org)
Phantom Irradiation	X	An IMRT phantom study provided by the IROC QA Center Houston must be successfully completed. Instructions for requesting and irradiating the phantom are found on the IROC Houston web site (http://irochouston.mdanderson.org). Tomotherapy treatment delivery modality must be credentialed individually.
Credentialing Notification Issued to:		
Institution		IROC Houston QA Center will notify the institution and NRG Headquarters that all desired credentialing requirements have been met. The site will need to upload a PDF of the approval email from IROC Houston to the CTSU Regulatory Portal for RSS to be updated.

8.4.1 Digital RT Data Submission to NRG Using TRIAD

TRIAD is the image exchange application used by the NRG. TRIAD provides sites participating in NRG clinical trials a secure method to transmit DICOM RT and other objects. TRIAD anonymizes and validates the images as they are transferred.

TRIAD Access Requirements:

- Site physics staff who will submit images through TRIAD will need to be registered with The Cancer Therapy Evaluation Program (CTEP) and have a valid and active CTEP Identity and Access Management (IAM) account. Please refer to the beginning of Section 4 for instructions on how to request a CTEP-IAM account.

- To submit images, the site physics user must have been assigned the 'TRIAD site user' role on the relevant Group or CTSU roster. NRG users should contact your site Lead RA to be added to your site roster. Users from other cooperative groups should follow their procedures for assignment of roster roles.
- RAs are able to submit standard of care imaging through the same method.

<u>TRIAD Installations:</u>
When a user applies for a CTEP-IAM account with proper user role, he/she will need to have the TRIAD application installed on his/her workstation to be able to submit images. TRIAD installation documentation can be found by following this link: http://triadinstall.acr.org/triadclient/

This process can be done in parallel to obtaining your CTEP-IAM account username and password.

If you have any questions regarding this information, please send an e-mail to the TRIAD Support mailbox at TRIAD-Support@acr.org.

9. DRUG INFORMATION

9.1 Atezolizumab (NSC 783608)

Other Names: Tecentriq^{TM}, MPDL3280A

Classification: monoclonal antibody

M.W.: 150 KD

Mode of Action: anti-PD-L1

Description:
Atezolizumab is a humanized IgG1 monoclonal antibody consisting of two heavy chains (448 amino acids) and two light chains (214 amino acids). Atezolizumab targets human PD-L1 and inhibits its interaction with its receptor PD-1. Atezolizumab also blocks the binding of PD-L1 to B7.1, an interaction that is reported to provide additional inhibitory signals to T cells (Butte et al. 2007).

How Supplied:
Atezolizumab is provided by Genentech/F.Hoffmann-La Roche LTD and distributed by the Pharmaceutical Management Branch, CTEP, NCI. The agent is supplied in a single-use, 20-mL glass vial as a colorless-to-slightly-yellow, sterile, preservative-free clear liquid solution intended for IV administration. Atezolizumab is formulated as 60 mg/mL atezolizumab in 20 mM histidine acetate, 120 mM sucrose, 0.04% polysorbate 20, at a pH of 5.8. The vial is designed to deliver 20 mL (1200 mg) of atezolizumab solution but may contain more than the stated volume to enable delivery of the entire 20 mL volume.

Preparation:
The prescribed dose of atezolizumab should be diluted in 250 mL 0.9% NaCl and infused through a 0.2 micrometer in-line filter. The IV bag may be constructed of PVC or PO; the IV infusion line may be constructed of PVC or PE; and the 0.2 micrometer in-line filter may be constructed of PES. The prepared solution may be stored at 2°C-8°C or room temperature for up to 8 hours.

Storage: 2°C–8°C (36°F–46°F). Vial contents should not be frozen or shaken and should be protected from direct sunlight.

If a storage temperature excursion is identified, promptly return atezolizumab to 2°C-8°C (36°F-46°F) and quarantine the supplies. Provide a detailed report of the excursion (including documentation of temperature monitoring and duration of the excursion) to PMBAfterHours@mail.nih.gov for determination of suitability.

Stability: Stability studies are ongoing.

CAUTION: No preservative is used in atezolizumab; therefore, the vial is intended for single use only. Discard any unused portion of drug remaining in a vial.

Route of Administration: IV infusion

Method of Administration:
Atezolizumab is administered as an intravenous infusion over 60 minutes. If the first infusion is tolerated, all subsequent infusions may be delivered over 30 minutes. Do not administer atezolizumab as an intravenous push or bolus. No premedication is indicated for administration of Cycle 1 of atezolizumab. Patients who experience an infusion related reaction with Cycle 1 of atezolizumab may receive premedication with antihistamines or antipyretics/analgesics (e.g. acetaminophen) for subsequent infusions.

Potential Drug Interactions:
Cytochrome P450 enzymes as well as conjugation/glucuronidation reactions are not involved in the metabolism of atezolizumab. No drug interaction studies for atezolizumab have been conducted or are planned. There are no known interactions with other medicinal products or other form of interactions.

Patient Care Implications:
Male patients and female patients of childbearing potential should utilize contraception and take active measures to avoid pregnancy while undergoing atezolizumab treatment and for at least 5 months (150 days) after the last dose of atezolizumab.

Availability

Atezolizumab is an investigational agent supplied to investigators by the Division of Cancer Treatment and Diagnosis (DCTD), NCI.

Atezolizumab is provided to the NCI under a Collaborative Agreement between the Pharmaceutical Collaborator and the DCTD, NCI (see Appendix VI).

9.1.1 Agent Ordering and Agent Accountability

9.1.1.1 NCI-supplied agents may be requested by the Principal Investigator (or their authorized designee) at each participating institution. Pharmaceutical Management Branch (PMB) policy requires that agent be shipped directly to the institution where the patient is to be treated. PMB does not permit the transfer of agents between institutions (unless prior approval from PMB is obtained). The CTEP-assigned protocol number must be used for ordering all CTEP-supplied investigational agents. The responsible investigator at each participating institution must be registered with CTEP, DCTD through an annual submission of FDA Form 1572 (Statement of Investigator), Biosketch/Curriculum Vitae, Supplemental Investigator Data Form (IDF), and Financial Disclosure Form (FDF). If there are several participating investigators at one institution, CTEP-supplied investigational agents for the study should be ordered under the name of one lead investigator at that institution.

Sites may order atezolizumab after a patient has been enrolled into the study.

Active CTEP-registered investigators and investigator-designated shipping designees and ordering designees can submit agent requests through the PMB Online Agent Order Processing (OAOP) application. Access to OAOP requires the establishment of a CTEP Identity and Access Management (IAM) account and the maintenance of an “active” account status and a “current” password. For questions about drug orders, transfers, returns, or accountability, call or email PMB any time. Refer to the PMB’s website for specific policies and guidelines related to agent management.

9.1.1.2 Agent Inventory Records – The investigator, or a responsible party designated by the investigator, must maintain a careful record of the receipt, dispensing and final disposition of all agents received from the PMB using the appropriate NCI Investigational Agent (Drug) Accountability Record (DARF) available on the CTEP forms page. Store and maintain separate NCI Investigational Agent Accountability Records for each agent, strength, formulation and ordering investigator on this protocol.

9.1.2 Investigator Brochure Availability

The current versions of the IBs for the agents will be accessible to site investigators and research staff through the PMB OAOP application. Access to OAOP requires the establishment of a CTEP IAM account and the maintenance of an “active” account status and a “current” password. Questions about IB access may be directed to the PMB IB Coordinator via email.

9.1.3 Useful Links and Contacts

- CTEP Forms, Templates, Documents: http://ctep.cancer.gov/forms/
- NCI CTEP Investigator Registration: RCRHelpDesk@nih.gov
- PMB policies and guidelines:

http://ctep.cancer.gov/branches/pmb/agent_management.htm

- PMB Online Agent Order Processing (OAOP) application: https://ctepcore.nci.nih.gov/OAOP
- CTEP Identity and Access Management (IAM) account: https://ctepcore.nci.nih.gov/iam/
- CTEP IAM account help: ctepreghelp@ctep.nci.nih.gov
- IB Coordinator: IBCoordinator@mail.nih.gov
- PMB email: PMBAfterHours@mail.nih.gov
- PMB phone and hours of service: (240) 276-6575 Monday through Friday between 8:30 am and 4:30 pm (ET)

9.2 Cisplatin (NSC# 119875)

Formulation: PLATINOL®-AQ (cisplatin injection) infusion concentrate is a clear, colorless, sterile aqueous solution available in amber vials. Each 50 mL or 100 mL amber vial of infusion concentrate contains: 1 mg/mL cisplatin, 9 mg/mL sodium chloride, hydrochloric acid and sodium hydroxide to approximate pH of 4.0, and water for injection to a final volume of 50 mL or 100 mL, respectively.

Supplier: Commercially available. Refer to individual FDA-approved package insert.

Preparation: PLATINOL®-AQ (cisplatin injection) infusion concentrate (1 mg/mL) must be further diluted prior to administration. Cisplatin (40 mg/m^2, 70 mg maximum) will be diluted in 250-1000 mL of normal saline.

NOTE: Aluminum reacts with cisplatin causing precipitation formation and loss of potency, therefore, needles or intravenous sets containing aluminum parts that may come in contact with the drug must not be used for the preparation or administration of cisplatin.

Storage: Store at 15° to 25°C (59° to 77°F). Do not refrigerate. Protect unopened container from light.

Stability: The cisplatin remaining in the amber vial following initial entry is stable for 28 days protected from light or for 7 days under fluorescent room light.

Administration: Once weekly cisplatin (40 mg/m^2, 70 mg maximum) is administered intravenously on Days 0, 7, 14, 21, 28, and 35. An infusion of 1000 ml of ½ normal saline should be given intravenously one hour before cisplatin. Increased oral intake

should be encouraged starting the day before. Additional fluid may be given as needed for symptomatic support. Cisplatin should be infused at a rate of 1 mg/min, usually over a total of 90 minutes or as per institutional protocol. Immediately after completion of the cisplatin infusion, an additional 1000 ml of ½ normal saline should be given or as per institutional protocol.

Adverse effects:

Infrequent: Cardiac abnormalities, anorexia, elevated SGOT, rash, alopecia, and acute myeloid leukemia.

NOTE: Aminoglycoside antibiotics given before, with, or after cisplatin may potentiate renal toxicity and should be avoided whenever possible.

9.2.1 Adverse Events

Please refer to the package insert for complete discussion of adverse events. Leukopenia, thrombocytopenia, anemia, nausea, vomiting, nephrotoxicity, ototoxicity, peripheral neuropathy, electrolyte imbalance, hypocalcemia, hypomagnesemia, aminoglycoside ototoxicity, ocular toxicity, and allergic reactions.

Refer to the package insert for detailed pharmacologic and safety information

9.2.2 Availability/Supply

Please see Section 9.2 for administration instructions. Please refer to the current FDA-approved package insert provided with each drug and the site-specific pharmacy for toxicity information and instructions for drug preparation, handling, and storage.

[If off-label, the following statement must be included for each IND exempt drug]: The use of drug(s) or combination of drugs in this protocol meet the criteria described under Title 21 CFR 312.2(b) for IND exemption.)

10. PATHOLOGY/BIOSPECIMEN

10.1 Central Pathology Review Guidelines

Not applicable

10.2 Biomarker Selection for Integral Biomarker Testing

10.2.1 Biomarker to be Tested

To satisfy the primary objective of the study, the following will be assessed in response to sequential versus concurrent atezolizumab and radiation: (1) peripheral blood T Cell Receptor (TCR) clonality, and (2) peripheral blood expansion of TCR clones, including TCR clones identified in tumor samples.

10.2.2 Testing Requirements and Reporting

For assessment of the primary endpoint, pre-treatment FFPE tumor biopsies (to determine

which TCR clones are present within the tumor), as well as pre-treatment and on-treatment bloods will be required.

10.2.3 Method of Testing

The NRG Oncology Biospecimen Bank (NRG BB)-Columbus will batch ship, every six months, unstained sections of pre-treatment FFPE tumor biopsies to the laboratory of Dr. Dmitriy Zamarin. Quality of FFPE tissue (degree of tumor content and necrosis) will be determined by a board-certified pathologist working with Dr. Dmitriy Zamarin based on H&E testing of a representative section from each tumor. Tumor areas appropriate for analysis will be macrodissected and DNA will be extracted using Qiagen kit in Dr. Zamarin's laboratory. DNA quality and quantity will be assured by OD 260/280 and OD 260/230 ratios. DNA extracted from baseline tumor tissue will be shipped to Adaptive Biotechnologies for TCR sequencing.

The NRG BB-Columbus will batch ship, every six months, pre-treatment and day 21 frozen whole blood to Adaptive Biotechnologies for DNA extraction and quality confirmation using OD 260/280 and OD 260/230 ratios. This will be followed by deep sequencing of CDR3 regions. Rearranged TCRbeta CDR3 sequences will be amplified and sequenced using the Adaptive Biotechnologies immunoSEQ platform. Absolute counts and frequencies of individual clone templates will be determined on a per sample basis using a spike-in control repertoire. Additional internal controls also report the quantification of sampled T cells and total nucleated cells. Processing will be per the Adaptive Biotechnologies standard Operating Procedure (SOP) for the One-Reaction protocol.

10.2.4 Location of Testing

DNA extraction from pre-treatment tumor tissue will be performed in the laboratory of Dr. Dmitriy Zamarin (Memorial Sloan Kettering Cancer Center; MSKCC). DNA extraction from pre-treatment and day 21 whole blood will be done at Adaptive Biotechnologies. TCR sequencing of DNA from both tumor tissue and whole blood will be done at Adaptive Biotechnologies

10.2.5 Biospecimen Submission for Testing

See Mandatory Biospecimen Submission Table (section 10.4.1) for details.

10.3 Biospecimen Selection for Integrated Biomarker Testing

Note: Exploratory biomarker testing of banked specimens will not occur until an amendment to this treatment protocol (or separate correlative science protocol at a later date) is reviewed and approved in accordance with National Clinical Trials Network (NCTN) policies.

10.3.1 Biomarker to be Tested

PD-L1

10.3.2 Testing Requirements and Reporting

FFPE tumor biopsies will be used for retrospective PD-L1 testing. See Mandatory Biospecimen Submission Table (section 10.4.1) for details.

10.3.3 **Method of Testing**

VENTANA PD-L1 (SP142), which is FDA approved as a complimentary diagnostic immunohistochemistry (IHC) assay to ascertain tumor PD-L1 status for patients with metastatic urothelial cancer considering treatment with atezolizumab. In ovarian cancer patients, SP142 detects PD-L1 on immune cells (Webb, et al, 2016).

Briefly, PD-L1 expression will be assessed using the rabbit anti-human PD-L1 antibody (clone SP142). Using IHC, PD-L1 expression will be scored in tumor cells (as percentage of PD-L1-expressing tumor cells: TC3≥50%, TC2≥5% and <50%, TC1≥1% and <5%, and TC0<1%) and tumor infiltrating immune cells (as percentage of tumor area: IC3≥10%, IC2≥5% and <10%, IC1≥1% and <5%, and IC0<1%).

10.3.4 **Location of Testing**

The NRG BB-Columbus will batch ship, every six months fresh cut (not older than 60 days) sections of FFPE tumor biopsies to the laboratory of Dr. Dmitriy Zamarin. Testing will be performed using the commercial kit and PD-L1 positivity will be scored by a board-certified pathologist.

10.3.5 **Biospecimen Submission for Testing**

FFPE tumor **blocks** must be shipped to the NRG BB-Columbus. See Mandatory Biospecimen Submission Table (section 10.4.1) for details.

10.4 Biospecimen Submission Tables

Biospecimens listed below cannot be submitted until after patient registration and Bank ID assignment.

A detailed description of biospecimen procedures can be found in Appendix I and below

10.4.1 Mandatory Biospecimen Submissions

The patient must give permission to participate in this **mandatory** study component. Participating sites are required to submit the patient's biospecimens as outlined below.

Required Biospecimen (Specimen Code)	Collection Time Point	Sites Ship Biospecimens To
**DAY -21 (Regimen A Only)**		
Tissue		
D-21 FFPE Primary Tumor Biopsy (FP01)[1]<br>Block must be submitted[2]	Biopsy collected after randomization, prior to patient receiving any study treatment (day -21 ± 3 days).<br><br>Submit *one*, FP01 preferred.	NRG BB-Columbus within 8 weeks of registration[3]
D-21 FFPE Metastatic Tumor Biopsy (FM01)[1]<br>Block must be submitted[2]		
Blood		
D-21 Plasma (PB01)<br>prepared from 7-10mL of blood drawn into purple top (K2EDTA) tube(s)[4]	Day -21, prior to atezolizumab.	NRG BB-Columbus within 4 weeks of registration[3]
D-21 Buffy Coat (LB01)<br>prepared from 7-10mL of blood drawn into purple top (K2EDTA) tube(s)[4]		

D-21 Whole Blood (WB01)<br>7-10mL drawn into purple top (K2EDTA) tube(s) and frozen		
**DAY 0 (Regimen A and B)**		
Tissue		
D0 FFPE Primary Tumor Chemoradiation Biopsy (FP02)[1]<br>Block must be submitted[2]	Biopsy collected at start of chemoradiation (day 0 ± 3 days).<br><br>Submit *one*, FP02 preferred.	NRG BB-Columbus within 8 weeks of registration[3]
D0 FFPE Metastatic Tumor Chemoradiation Biopsy (FM02)[1]<br>Block must be submitted[2]		
Blood		
D0 Plasma (PB02)<br>prepared from 7-10mL of blood drawn into purple top (K2EDTA) tube(s)[4]	Day 0 ± 3 days.	NRG BB-Columbus within 4 weeks of registration[3]
D0 Buffy Coat (LB02)<br>prepared from 7-10mL of blood drawn into purple top (K2EDTA) tube(s)[4]		
D0 Whole Blood (WB02)<br>7-10mL drawn into purple top (K2EDTA) tube(s) and frozen		
**DAY 21 (Regimen A and B)**		
Blood		
D21 Plasma (PB03)<br>prepared from 7-10mL of blood drawn into purple top (K2EDTA) tube(s)[4]	Day 21 ± 1 day.	NRG BB-Columbus within 4 weeks of registration[3]
D21 Buffy Coat (LB03)<br>prepared from 7-10mL of blood drawn into purple top (K2EDTA) tube(s)[4]		
D21 Whole Blood (WB03)<br>7-10mL drawn into purple top (K2EDTA) tube(s) and frozen		
**DAY 28 (Regimen A and B)**		
Tissue		
D28 FFPE Primary Tumor Brachytherapy Biopsy (FP03)[1]<br>Block must be submitted[2]	Biopsy collected at first brachytherapy (day 28 ± 7 days).<br><br>Submit *one*, FP03 preferred.	NRG BB-Columbus within 8 weeks of registration[3]
D28 FFPE Metastatic Tumor Brachytherapy Biopsy (FM03)[1]<br>Block must be submitted[2]		
**DAY 42 (Regimen A and B)**		
Blood		
D42 Plasma (PB04)<br>prepared from 7-10mL of blood drawn into purple top (K2EDTA) tube(s)[4]	Day 42 ± 7 days; at start of brachytherapy.	NRG BB-Columbus within 10 weeks of registration[3]
D42 Buffy Coat (LB04)<br>prepared from 7-10mL of blood drawn into purple top (K2EDTA) tube(s)[4]		
D42 Whole Blood (WB04)<br>7-10mL drawn into purple top (K2EDTA) tube(s) and frozen		
**DAY 63 (Regimen A and B)**		
Blood		
D63 Plasma (PB05)<br>prepared from 7-10mL of blood drawn into	Day 63 ± 3 days.	NRG BB-Columbus within 10 weeks of registration[3]

purple top (K2EDTA) tube(s) [4]		
D63 Buffy Coat (LB05)<br>prepared from 7-10mL of blood drawn into purple top (K2EDTA) tube(s) [4]		
D63 Whole Blood (WB05)<br>7-10mL drawn into purple top (K2EDTA) tube(s) and frozen		
**DAY 140 (Regimen A and B)**		
Blood		
D140 Plasma (PB06)<br>prepared from 7-10mL of blood drawn into purple top (K2EDTA) tube(s) [4]	Day 140 ± 7 days; at time of post therapy PET/CT.	NRG BB-Columbus within 24 weeks of registration[3]
D140 Buffy Coat (LB06)<br>prepared from 7-10mL of blood drawn into purple top (K2EDTA) tube(s) [4]		
D140 Whole Blood (WB06)<br>7-10mL drawn into purple top (K2EDTA) tube(s) and frozen		

1 A copy of the corresponding pathology report must be shipped with all tissue biospecimens sent to the NRG BB-Columbus. **If a pathology report if not available for the biopsy, a copy of the radiology report or operative report from the biopsy procedure must be sent to the NRG BB-Columbus, along with a completed copy of the Biopsy Pathology Verification (Appendix V).**

2 **Only blocks will be accepted. Please provide Appendix V to your pathologist.**

3 NRG BB-Columbus / Protocol NRG-GY017, Nationwide Children's Hospital, 700 Children's Drive, WA1340, Columbus, OH 43205, Phone: (614) 722-2865, FAX: (614) 722-2897, Email: BPCBank@nationwidechildrens.org

4 Plasma and buffy coat biospecimens are processed from the same blood collection tube(s).

11. MODALITY REVIEWS

Radiation Therapy Quality Assurance Reviews

The study PI (Jyoti Mayadev) with NRG radiation oncologists will perform pre-treatment RT Quality Assurance Review. Brachytherapy cases will be reviewed Post Hoc.

The scoring mechanism is: Per Protocol, Variation Acceptable, Deviation Unacceptable.

> NOTE: Pre-Treatment reviews are required for every IMRT case. The patient cannot begin treatment until complete data from the site is received, reviewed and approved.

Please allow three (3) business days for this to be completed. If a resubmission is required, the three (3)-business day timeline will restart. If the patient is scheduled to start treatment on a Monday the plan must be submitted by Tuesday, COB of the prior week. Treatment cannot begin until approval from IROC Phila has been received at the site.

12. DATA AND RECORDS

12.1 Data Management/Collection

Data collection for this study will be done exclusively through Medidata Rave®. Access to the trial in Rave is granted through the iMedidata application to all persons with the

appropriate roles in RSS (Regulatory Support System). To access iMedidata/Rave, the site user must have an active CTEP-IAM account and the appropriate Rave role (Rave CRA, Read-Only, Site Investigator) on either the LPO or participating organization rosters at the enrolling site.

Upon initial site registration approval for the study in RSS, all persons with Rave roles assigned on the appropriate roster will be sent a study invitation e-mail from iMedidata (iMedidata-Notification@mdsol.com) to activate their account. To accept the invitation, site users must log into the Select Login (https://login.imedidata.com/selectlogin) using their CTEP-IAM user name and password, and click on the "accept" link in the upper right-corner of the iMedidata page. Please note, site users will not be able to access the study in Rave until all required Medidata and study specific trainings are completed. Trainings will be in the form of electronic learnings (eLearnings) and will be listed in the upper right pane of the iMedidata screen.

Users that have not previously activated their iMedidata/Rave accounts also will receive a separate invitation from iMedidata to activate their account. Account activation instructions are located on the CTSU website, Rave tab under the Rave resource materials (Medidata Account Activation and Study Invitation Acceptance). Additional information on iMedidata/Rave is available on the CTSU website under the Rave tab at www.ctsu.org/RAVE/ or by contacting the CTSU Help Desk at 1-888-823-5923 or by e-mail at ctsucontact@westat.com.

12.2 Summary of Data Submission

Adverse event data collection and reporting, which are required as part of every clinical trial, are done to ensure the safety of patients enrolled in the studies as well as those who will enroll in future studies using similar agents. Adverse events are reported in a routine manner at scheduled times during the trial using Medidata Rave®. Additionally, certain adverse events must be reported in an expedited manner for timelier monitoring of patient safety and care. See Sections 7 and 7.6 for information about expedited and routine reporting.

Summary of Data Submission: Refer to the NRG/CTSU website.

See Section 8.4 for TRIAD account access and installation instructions.

12.3 Global Reporting/Monitoring

This study will be monitored by the Clinical Data Update System (CDUS) version 3.0. Cumulative CDUS data will be submitted quarterly to CTEP by electronic means. Reports are due January 31, April 30, July 31, and October 31.

Note: If your study has been assigned to CDUS-Complete reporting, **all** adverse events (both routine and expedited) that have occurred on the study and meet the mandatory CDUS reporting guidelines must be reported via the monitoring method identified above. If your study has been assigned to CDUS-Abbreviated reporting, no adverse event reporting (routine or expedited) is required to be reported via CDUS, but expedited

adverse events are still required to be submitted via CTEP-AERS.

13. STATISTICAL CONSIDERATIONS

13.1 Study Design

The primary objective of this study is to assess whether there is a difference of immune expression between the two treatment arms through T cell receptor beta (TCRB) clonal expansion in peripheral blood. This study is designed as a two-arm, open label, randomized pilot trial with two experimental arms, where arm A will get one dose of Atezolizumab prior to cisplatin chemotherapy and radiation therapy, and then two subsequent doses of Atezolizumab during the chemotherapy and radiation therapy, and arm B will get 3 doses during the cisplatin chemotherapy and radiation therapy.

Prior to patient registration, eligibility will be reviewed by Entry Form verification. The sequence of treatment assignments will be concealed from institutions and patients until registration with verification of eligibility. Patients will be registered by the participating site through OPEN and randomization will be carried out centrally by the NRG Statistics and Data Management Center. Treatment group assignment will be determined using a procedure that tends to randomly allocate study treatment s at a 1 to 1 ratio.

13.2 Study Endpoints

Primary Endpoint

1) Measurements of TCRB clonal expansion in peripheral blood at day 21.

Secondary Endpoints

1) DLTs occurring during the period time from the start of first dose of atezolizumab (arm A) or the start of CRT treatment (arm B) until 30 days after the completion of CRT.
2) Frequency and severity of adverse events as assessed by CTCAE v5.
3) Measurements of TCR clonality, diversity and frequency in peripheral blood/-tissue at each protocol-specified collecting time point and measures of PET-CT scan at post-therapy 12 weeks (exploratory and optional), and 2-year clinical DFS.

4) Measurements of PD-L1 expression in tissue at each protocol-specified collecting time point, measurements of post-treatment week 12 PET-CT scan (exploratory and optional), and 2-year clinical DFS.

Exploratory Science Endpoints

1) Measurements of biomarkers from blood/ tissue at baseline and on-treatment, measures of PET-CT scan at post-therapy 12 weeks (day 140), and 2-year DFS.
2) Response assessment on the post-treatment week 12 PET-CT scan (exploratory and optional) and 2-year clinical DFS.

13.3 Primary Objectives Study Design

13.3.1 <u>Primary Hypothesis and Endpoints</u>

The primary objective of this study is to determine whether there is a difference of immune expression between the two treatment arms through T cell receptor beta (TCRB) clonal expansion in peripheral blood at day 21. It is hypothesized that a treatment with increased clinical benefit will generate more clonal expansion in peripheral blood or more expansion of tumor-associated clones in peripheral blood. Therefore, the evaluation of the difference of immune expression between the two treatment arms measured by TCRB clonal expansion in peripheral blood will be formulated through hypothesis testing via the difference of the means. The primary hypotheses for this study are

$$H0: \mu_A = \mu_B,$$

$$H1: \mu_A \neq \mu_B,$$

where μ_A and μ_B are the means of the TCRB in peripheral blood for arm A and arm B, respectively.

Assume that the TCRB is normally distributed in arm A and arm B with an equal standard deviation (σ). A two-sided t-test will be used for testing the primary hypotheses at 10% significance level. The clinically interested effect size (δ) is 0.95, i.e., the difference of means is 0.95 times of the standard deviation. A sample size of 40 patients (20 in each treatment arm) will give this study a 90% power to detect an effect size of 0.95 by a two-sided t-test at significance level of 0.1. Figure 1 shows the estimated power of this study versus the effect size by the number of patients enrolled on each arm.

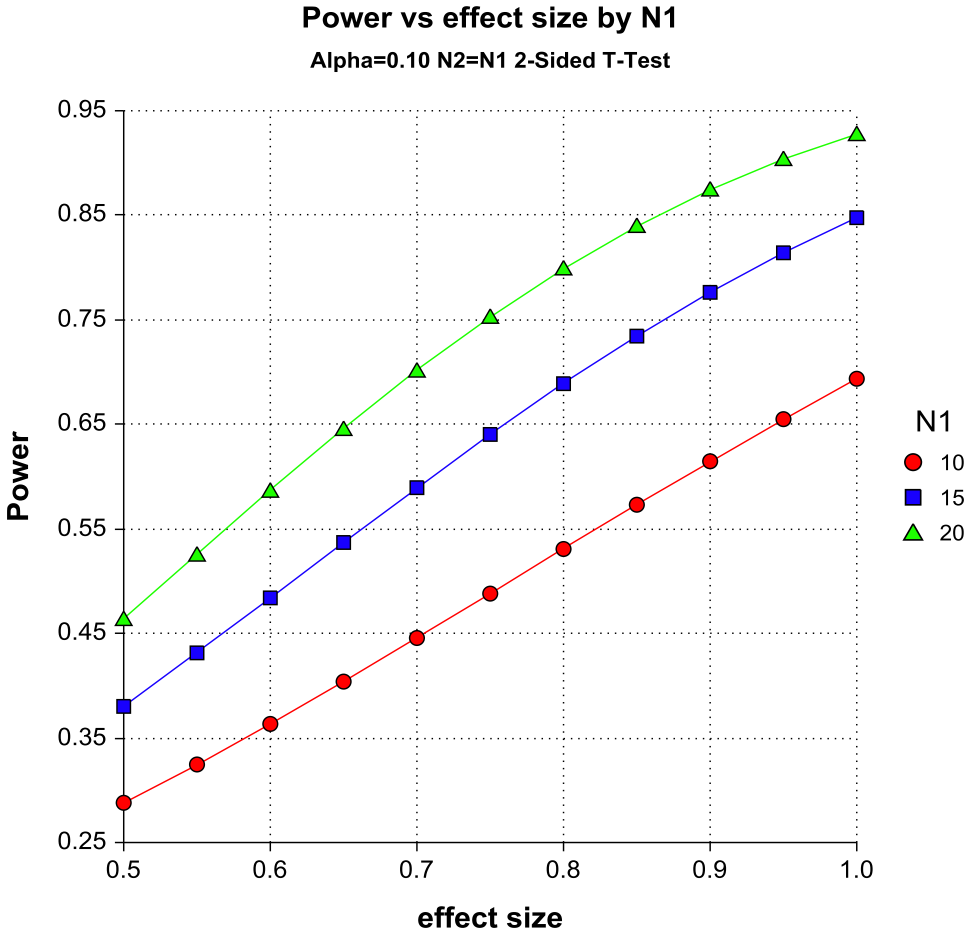

Figure 1

Based on prior experience in GOG-9929, it is expected that at least 50% of the enrolled patients are evaluable for DLT, thus, a sample size of 40 patients (20 in each treatment arm) will ensure at least 10 DLT-evaluable patients for each regimen.

13.3.2 How Primary Endpoints Will Be Analyzed

All eligible patients who received Arm A (dose at day -21 and dose at day 0) and Arm B (dose at day 0) and have TCRB measurements will be evaluable. For the primary objective, there will be a comparison of the arms at day 21 collection by 2-sided t-test at 10% significance level. Based on the mechanism of action of PD-L1 inhibitors, it is unlikely that therapeutic dose would have an immediate effect on the translational efficacy parameters (dose given at day 21). However, to ensure that this does not confound the findings, all labs for efficacy assessment will be collected prior to treatment administration.

13.3.3 Sample Size and Power Calculations:

The sample size and power calculation is based on 2-sided t-test under the assumptions that the TCRB is normally distributed in arm A and arm B with an equal standard

deviation (σ). The target accrual of this study is 40 patients (20 in each treatment arm).

A sample size of 40 patients (20 in each treatment arm) will give this study a 90% power to detect an effect size of 0.95 by a two-sided t-test at significance level of 0.1.

Due to potential attrition, a feasibility assessment of the proportion of patients who are either ineligible or do not have their bio-specimen for evaluation of the primary endpoints submitted will be evaluated at the study accrual of 20 patients. The proportion of patients experiencing at least one DLT in DLT-evaluable patients will be estimated by treatment arm at this time, and the frequency and severity of all toxicities will be tabulated by treatment arm for all patients who receive any study treatment. If either the proportion of attrition is higher than 25% or the proportion of patients with DLT is higher than 30%, then the principal investigators will be contacted, and conference calls will be scheduled among the principal investigators and study team.

If the proportion of patients with attrition is 25% or less, a sample size of 40 (20 in each arm) will results in at least 30 eligible and evaluable patients (15 in each arm) which would give the study at least 81% power to detect the effect size of 0.95 at significance level of 0.1 using a two-sided t-test.

13.4 Study Monitoring of Primary Objectives

(Interim Analysis)

Interim Analysis for the DMC

There is no formal interim analysis planned. The NRG Oncology Data Monitoring Committee (DMC) will review the study twice a year with respect to patient accrual and morbidity. The DMC also will review the study on an "as needed" basis.

13.5 Accrual/Study Duration Considerations

The accrual rate for this study is projected to be approximately 3 patients per month based on GOG-9929 with adjustment of holding time.

This study will take approximately 16 months to accrue the targeted sample size of 40 patients including a 2 to 3 months of start-up time with little to no accrual. The duration of study treatment is about 20 weeks, including FDG PET/CT at post-treatment 12 weeks. Final analysis will require distribution of specimens, resolution of queries, completion of all laboratory tests, and collation of laboratory data with clinical data. These can be accomplished within 12-18 months of final patient entry.

13.6 Dose Level Guidelines

There are no dose level changes defined in this study.

13.7 Secondary or Exploratory Endpoints (including correlative science aims)

13.7.1 Secondary Hypotheses and Endpoints:

The secondary objectives focus on estimation and description; there are no specific hypotheses.

Secondary endpoints see Section 13.2.

13.7.2 Definitions of Secondary Endpoints and How These Will Be Analyzed

DLT is defined in Section 5.7. The number of patients who experience DLTs will be summarized in the DLT-evaluable patients by treatment arm, the corresponding proportion of patients with DLTs and 90% confidence interval will be estimated.

Adverse events are assessed by NCI CTCAE v5. The frequency and maximum severity of acute adverse events will be tabulated by treatment arm graded by CTCAE v5.

All patients who receive any study drug will be evaluable for toxicity. The DLT-evaluable patients will only include those eligible patients who have a DLT and have received at least one dose of Atezolizumab, or complete assigned protocol therapy and are evaluable at the DLT specified time period, where the DLT period for Arm A: start of the priming dose of atezolizumab until 30 days after the completion of CRT; for Arm B: start of CRT until 30 days after the completion of CRT.

Disease-free survival (DFS) is defined as the duration of time from study entry to date of first documented recurrence or progression of disease or death, whichever occurs first. DFS is censored in patients who are alive and disease have not recurred or progressed.

Summary statistics (and graph where it is appropriate) for the measurements of TCR clonality, diversity and frequency in peripheral blood/tissue at each protocol-specified collecting time point, measures of PET-CT scan at post-therapy 12 weeks, and the proportion of patients who are alive and disease-free for at least 2 years will be provided by treatment arm. If feasible, repeated measure techniques will be applied to investigate the changes and the trajectories of TCR clonality, diversity, and frequency in peripheral blood by treatment arm, and mixed modeling may be used to explore the corresponding associations with measurement for PET-CT scan at post-therapy 12 weeks and 2-year DFS.

PD-L1 expression in the tissue by treatment arm will be summarized for each protocol-specified collecting time point. Spearman's correlation coefficient will be used to assess the correlation of PD-L1 expression in the tissue at each protocol-specified collecting time point by arm with DFS at 2 years and measurement for post-treatment PET-CT, respectively. The maximum number of patients with tumor tissues at each collecting time is 20 for each arm.

There are no formal specific hypotheses regarding PD-L1 expression in tumor tissue, but it is hypothesized that a patient with higher pre-treatment PD-L1 expression in tumor tissue will have better clinical responses that are measured by disease-free survival at 2 years and post-treatment PET-CT in the treatment with Pembrolizumab. Figure 2 shows the power to detect a series of Spearman's rank correlation coefficients from the Spearman's rank correlation coefficient (ρ) of 0 by different sample size using an asymptotic one-sided test at 10% significance level. A sample size of 20 will provide

approximately at least 80% power to detect a Spearman's rank correlation coefficient of 0.5 or higher from ρ = 0 using a one-sided test at 10% significance level.

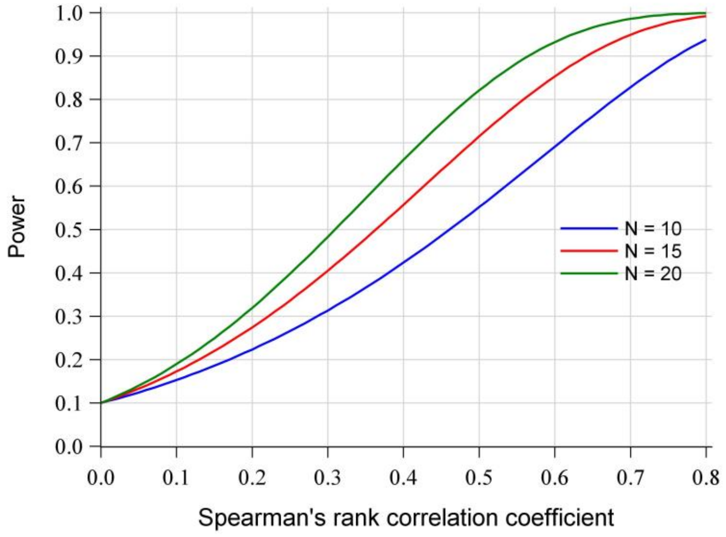

Figure 2

The purpose of secondary objectives is to estimate and generate hypotheses for future study. Therefore, there will be no adjustment for multiple tests. Due to small sample size of this study, exact testing procedure (i.e., exact Spearman correlation test) may be used where it is appropriate.

13.7.3 Interim Analysis for All Other Endpoints (Goals):
No interim analyses are planned for secondary endpoints.

13.7.4 Power Calculations:
As there are no specific hypotheses for the secondary endpoints, no power calculations are done.

13.7.5 Data and Safety Monitoring:
Clinical data collected on this protocol will be reviewed by the study data manager and will also be reviewed by the Study Chairperson in conjunction with the Statistics and Data Management Center (SDMC) on an ongoing basis. In some instances, because of unexpectedly severe toxicity, early closure of a study may be elected.

The frequency and severity of all toxicities are tabulated from submitted case report forms and summarized for review by the study chairperson, disease site committee and the committee charged with monitoring safety in conjunction with each semi-annual meeting.

All serious adverse events (SAEs) are reported to the Study Chair, Sponsor, and regulatory agencies as mandated in the protocol. SAE reports are reviewed by the Study Chair (or designated co-chair) immediately for consideration of investigator notification of a suspected unexpected serious adverse reaction (SUSAR), protocol amendment, and/or

immediate study suspension. All participating institutions will receive notification of the SUSAR from NRG as well as the reason for study suspension (if applicable). Under these circumstances, accrual cannot be re-activated until the study is reviewed by the committee charged with monitoring safety. However, patients currently receiving treatment may continue to receive treatment in accordance with protocol guidelines at the discretion of their physicians, unless directed otherwise.

13.8 Exploratory Hypothesis and Endpoints

Exploratory endpoints see Section 13.2.

Summary statistics (and graphs where it is appropriate) of exploratory endpoints will be done by treatment arm.

Spearman's correlation coefficient will be implemented to explore the associations of biomarkers measured at baseline or on-treatment from blood and tissue with 2-year DFS and PET scan at post-therapy 12 weeks (day 140), respectively, by treatment arm.

Log-rank test or Cox PH model will be used to explore the associations of biomarkers measured at baseline or on-treatment from blood and tissue with DFS by treatment arm.

The association of 2-year DFS with the response assessment on the post-treatment week-12 PET-CT scan in each treatment arm will be examined with Chi-squared tests. The response assessment on the post-treatment week-12 PET-CT scan is evaluated based on the ratio of post-treatment week-12 PET-CT SUVmax to base-line PET-CT scan SUV max: the response will be classified as complete metabolic response for the ratio < 0.34, or classified as partial metabolic response for 0.34 <= the ratio < 0.76, or classified as stable metabolic response for 0.76 <= the ratio < 1.25, or classified as progressive metabolic disease for the ratio >= 1.25 [36]

The purpose of exploratory objective is to explore and possibly generate hypotheses for future study. Therefore there will be no adjustment for multiple tests.

13.9 Gender/Ethnicity/Race Distribution

**Racial Categories**	**DOMESTIC PLANNED ENROLLMENT REPORT**				
	**Ethnic Categories**				
	Not Hispanic or Latino		Hispanic or Latino		Total
	Female	Male	Female	Male	
American Indian/Alaska Native	1		0		1
Asian	3		0		3
Native Hawaiian or Other Pacific Islander	0		0		0

Black or African American	6		0		6
White	20		10		30
More Than One Race	0		0		0
Total	30		8		40

PHS 398 / PHS 2590 (Rev. 08/12 Approved Through 8/31/2015) OMB No. 0925-0001/0002

Stratification Factors
There are no stratification factors in this randomized two-arm trial.

14. REFERENCES

Blank, C., T.F. Gajewski, A. Mackensen. (2005). Interaction of PD-L1 on tumor cells with PD-1 on tumor-specific T cells as a mechanism of immune evasion: implications for tumor immunotherapy. *Cancer Immunol Immunother*. 54:307-314.

Clifford, G. M., J. S. Smith, M. Plummer, N. Munoz and S. Franceschi (2003). "Human papillomavirus types in invasive cervical cancer worldwide: a meta-analysis." Br J Cancer **88**(1): 63-73.

Cogliano, V., R. Baan, K. Straif, Y. Grosse, B. Secretan, F. El Ghissassi and W. H. O. I. A. f. R. o. Cancer (2005). "Carcinogenicity of human papillomaviruses." Lancet Oncol **6**(4): 204.

Davidson, M. T., J. Yuen, D. D'Souza and D. L. Batchelar (2008). "Image-guided cervix high-dose-rate brachytherapy treatment planning: Does custom computed tomography planning for each insertion provide better conformal avoidance of organs at risk?" Brachytherapy **7**: 37-42.

Deng, L., H. Liang, B. Burnette, M. Beckett, T. Darga, R. R. Weichselbaum and Y. X. Fu (2014). "Irradiation and anti-PD-L1 treatment synergistically promote antitumor immunity in mice." J Clin Invest **124**(2): 687-695.

Di Giacomo, A.M., M. Biagioli, M. Maio. (2010). The emerging toxicity profiles of anti–CTLA-4 antibodies across clinical indications. *Semin Oncol.* 37:499-507.

Dovedi, S. J., A. L. Adlard, G. Lipowska-Bhalla, C. McKenna, S. Jones, E. J. Cheadle, I. J. Stratford, E. Poon, M. Morrow, R. Stewart, H. Jones, R. W. Wilkinson, J. Honeychurch and T. M. Illidge (2014). "Acquired resistance to fractionated radiotherapy can be overcome by concurrent PD-L1 blockade." Cancer Res **74**(19): 5458-5468.

Eisenhauer, E.A., P. Therasse, J. Bogaerts, *et al*. (2009). New response evaluation criteria in solid tumours: revised RECIST guideline (version 1.1). *Eur J Cancer*. 45:228-247.

Gao, M., K. Albuquerque, A. Chi and I. Rusu (2010). "3D CT-based volumetric dose assessment of 2D plans using GEC-ESTRO guidelines for cervical cancer brachytherapy." Brachytherapy **9**(1): 55-60.

Grover, S., M. M. Harkenrider, L. P. Cho, B. Erickson, C. Small, W. Small, Jr. and A. N.

Viswanathan (2016). "Image Guided Cervical Brachytherapy: 2014 Survey of the American Brachytherapy Society." Int J Radiat Oncol Biol Phys **94**(3): 598-604.

Haie-Meder, C., R. Potter, E. Van Limbergen, E. Briot, M. De Brabandere, J. Dimopoulos, I. Dumas, T. P. Hellebust, C. Kirisits, S. Lang, S. Muschitz, J. Nevinson, A. Nulens, P. Petrow, N. Wachter-Gerstner and G. E. C. E. W. G. Gynaecological (2005). "Recommendations from Gynaecological (GYN) GEC-ESTRO Working Group (I): concepts and terms in 3D image based 3D treatment planning in cervix cancer brachytherapy with emphasis on MRI assessment of GTV and CTV." Radiother Oncol **74**(3): 235-245.

Hallahan, D., J. Kuchibhotla and C. Wyble (1996). "Cell adhesion molecules mediate radiation-induced leukocyte adhesion to the vascular endothelium." Cancer Res **56**(22): 5150-5155.

Hamanishi, J., M. Mandai, M. Iwasaki, *et al.* (2007). Programmed cell death 1 ligand 1 and tumor-infiltrating CD8+ T lymphocytes are prognostic factors of human ovarian cancer. *Proc Natl Acad Sci USA*. 104:3360-3365.

Hanks, G. E., D. F. Herring and S. Kramer (1983). "Patterns of care outcome studies. Results of the national practice in cancer of the cervix." Cancer **51**(5): 959-967.

Herbst, R.S., J-C. Soria, M. Kowanetz, *et al*. (2014). Predictive correlates of response to the anti-PD-L1 antibody MPDL3280A in cancer patients. *Nature*. 515:563-567.

Hino, R., K. Kabashima, Y. Kato, *et al.* (2010). Tumor cell expression of programmed cell death-1 ligand 1 is a prognostic factor for malignant melanoma. *Cancer.* 116:1757-1766.

Investigator's Brochure, Atezolizumab (MPDL3280A). Version 9, August 2016.

Keir, M.E., M.J. Butte, G.J. Freeman, *et al.* (2008). PD-1 and its ligands in tolerance and immunity. *Annual Rev Immunol*. 26:677-704.

Keys, H. M., B. N. Bundy, F. B. Stehman, L. I. Muderspach, W. E. Chafe, C. L. Suggs, 3rd, J. L. Walker and D. Gersell (1999). "Cisplatin, radiation, and adjuvant hysterectomy compared with radiation and adjuvant hysterectomy for bulky stage IB cervical carcinoma." N Engl J Med **340**(15): 1154-1161.

Kidd, E., B. Siegel, F. Dehdashti and e. al (2010). "Clinical outcomes of definitive intensity-modulated radiation therapy with fluorodeoxyglucose-positron emission tomography simulation in patients with locally advanced cervical cancer." Int J Radiat Oncol Biol Phys **77**: 1085-1091.

Klopp, A., J. Moughan, L. Portelance, B. Miller, M. Salehpour, D. D'Souza, L. Souhami, W. Small, R. Gaur and A. Jhingran (2010). "Hematologic toxicity on RTOG 0418: A phase II study of post-operative IMRT for gynecologic cancer." Int J Radiat Oncol Biol Phys: S121: 258.

Lee, Y., S. L. Auh, Y. Wang, B. Burnette, Y. Wang, Y. Meng, M. Beckett, R. Sharma, R. Chin, T. Tu, R. R. Weichselbaum and Y. X. Fu (2009). "Therapeutic effects of ablative radiation on

local tumor require CD8+ T cells: changing strategies for cancer treatment." Blood **114**(3): 589-595.

Liang, J. A., S. W. Chen, Y. C. Hung, L. S. Yeh, W. C. Chang, W. C. Lin and Y. Y. Chang (2014). "Low-dose, prophylactic, extended-field, intensity-modulated radiotherapy plus concurrent weekly cisplatin for patients with stage IB2-IIIB cervical cancer, positive pelvic lymph nodes, and negative para-aortic lymph nodes." Int J Gynecol Cancer **24**(5): 901-907.

Mayadev, J., A. Viswanathan, Y. Liu, C. S. Li, K. Albuquerque, A. L. Damato, S. Beriwal and B. Erickson (2017). "American Brachytherapy Task Group Report: A pooled analysis of clinical outcomes for high-dose-rate brachytherapy for cervical cancer." Brachytherapy **16**(1): 22-43.

Munoz, N., F. X. Bosch, S. de Sanjose, R. Herrero, X. Castellsague, K. V. Shah, P. J. Snijders, C. J. Meijer and G. International Agency for Research on Cancer Multicenter Cervical Cancer Study (2003). "Epidemiologic classification of human papillomavirus types associated with cervical cancer." N Engl J Med **348**(6): 518-527.

Okazaki, T., T. Honjo. (2007). PD-1 and PD-1 ligands: from discovery to clinical application. *Int Immunol*. 9:813-824.

Osborne, E. M., A. H. Klopp, A. Jhingran, L. A. Meyer and P. J. Eifel (2017). "Impact of treatment year on survival and adverse effects in patients with cervical cancer and paraortic lymph node metastases treated with definitive extended-field radiation therapy." Pract Radiat Oncol **7**(3): e165-e173.

Pavamani, S., D. P. D'Souza, L. Portelance, P. S. Craighead, A. G. Pearce, L. L. Traptow and C. M. Doll (2011). "Image-guided brachytherapy for cervical cancer: a Canadian Brachytherapy Group survey." Brachytherapy **10**(5): 345-351.

Potter, R., P. Georg, J. C. Dimopoulos, M. Grimm, D. Berger, N. Nesvacil, D. Georg, M. P. Schmid, A. Reinthaller, A. Sturdza and C. Kirisits (2011). "Clinical outcome of protocol based image (MRI) guided adaptive brachytherapy combined with 3D conformal radiotherapy with or without chemotherapy in patients with locally advanced cervical cancer." Radiother Oncol **100**(1): 116-123.

Potter, R., C. Haie-Meder and E. Limbergenc (2006). "Recommendations from gynaecological (GYN) GEC ESTRO workinggroup (II): Concepts and terms in 3D image-based treatment planning in cervix cancer brachytherapy—3D dose volume parameters and aspects of 3D image-based anatomy, radiation physics, radiobiology." Radiotherapy and Oncology **78** 67–77.

Potter, R., C. Kirisits, E. F. Fidarova, J. C. A. Dimopoulos, D. Berger, K. Tanderup and J. C. Lindegaard (2008). "Present status and future of high-precision image guided adaptive brachytherapy for cervix carcinoma." Acta Oncologica **47**(7): 1325-1336.

Powles, T., J.P. Eder, G.D. Fine, *et al*. (2014). MPDL3280A (anti-PD-L1) treatment leads to clinical activity in metastatic bladder cancer. *Nature.* 515:558-562.

Rose, P. G., B. N. Bundy, E. B. Watkins, J. T. Thigpen, G. Deppe, M. A. Maiman, D. L. Clarke-Pearson and S. Insalaco (1999). "Concurrent cisplatin-based radiotherapy and chemotherapy for locally advanced cervical cancer." N Engl J Med **340**(15): 1144-1153.

Sharabi, A. B., M. Lim, T. L. DeWeese and C. G. Drake (2015). "Radiation and checkpoint blockade immunotherapy: radiosensitisation and potential mechanisms of synergy." Lancet Oncol **16**(13): e498-509.

Sharon, E., H. Streicher, P. Goncalves and H. X. Chen (2014). "Immune checkpoint inhibitors in clinical trials." Chin J Cancer **33**(9): 434-444.

Snyder, A., T. Nathanson, S. A. Funt, A. Ahuja, J. Buros Novik, M. D. Hellmann, E. Chang, B. A. Aksoy, H. Al-Ahmadie, E. Yusko, M. Vignali, S. Benzeno, M. Boyd, M. Moran, G. Iyer, H. S. Robins, E. R. Mardis, T. Merghoub, J. Hammerbacher, J. E. Rosenberg and D. F. Bajorin (2017). "Contribution of systemic and somatic factors to clinical response and resistance to PD-L1 blockade in urothelial cancer: An exploratory multi-omic analysis." PLoS Med **14**(5): e1002309.

Stone, H. B., L. J. Peters and L. Milas (1979). "Effect of host immune capability on radiocurability and subsequent transplantability of a murine fibrosarcoma." J Natl Cancer Inst **63**(5): 1229-1235.

Thompson, R.H., S.M. Kuntz, B.C. Leibovich, *et al.* (2006). Tumor B7-H1 is associated with poor prognosis in renal cell carcinoma patients with long-term follow-up. *Cancer Res.* 66:3381-3385.

Topalian, S. L., F. S. Hodi, J. R. Brahmer, S. N. Gettinger, D. C. Smith, D. F. McDermott, J. D. Powderly, R. D. Carvajal, J. A. Sosman, M. B. Atkins, P. D. Leming, D. R. Spigel, S. J. Antonia, L. Horn, C. G. Drake, D. M. Pardoll, L. Chen, W. H. Sharfman, R. A. Anders, J. M. Taube, T. L. McMiller, H. Xu, A. J. Korman, M. Jure-Kunkel, S. Agrawal, D. McDonald, G. D. Kollia, A. Gupta, J. M. Wigginton and M. Sznol (2012). "Safety, activity, and immune correlates of anti-PD-1 antibody in cancer." N Engl J Med **366**(26): 2443-2454.

Tsai, K. K., I. Zarzoso and A. I. Daud (2014). "PD-1 and PD-L1 antibodies for melanoma." Hum Vaccin Immunother **10**(11): 3111-3116.

Twyman-Saint Victor, C., A. J. Rech, A. Maity, R. Rengan, K. E. Pauken, E. Stelekati, J. L. Benci, B. Xu, H. Dada, P. M. Odorizzi, R. S. Herati, K. D. Mansfield, D. Patsch, R. K. Amaravadi, L. M. Schuchter, H. Ishwaran, R. Mick, D. A. Pryma, X. Xu, M. D. Feldman, T. C. Gangadhar, S. M. Hahn, E. J. Wherry, R. H. Vonderheide and A. J. Minn (2015). "Radiation and dual checkpoint blockade activate non-redundant immune mechanisms in cancer." Nature **520**(7547): 373-377.

van Dyk, S., D. Byram and D. Bernshaw (2010). "Use of 3D imaging and awareness of GEC-ESTRO recommendations for cervix cancer brachytherapy throughout Australia and New

Zealand." J Med Imaging Radiat Oncol **54**(4): 383-387.

Vargo, J. A., H. Kim, S. Choi, P. Sukumvanich, A. B. Olawaiye, J. L. Kelley, R. P. Edwards, J. T. Comerci and S. Beriwal (2014). "Extended field intensity modulated radiation therapy with concomitant boost for lymph node-positive cervical cancer: analysis of regional control and recurrence patterns in the positron emission tomography/computed tomography era." Int J Radiat Oncol Biol Phys **90**(5): 1091-1098.

Varia, M. A., B. N. Bundy, G. Deppe, R. Mannel, H. E. Averette, P. G. Rose and P. Connelly (1998). "Cervical carcinoma metastatic to para-aortic nodes: extended field radiation therapy with concomitant 5-fluorouracil and cisplatin chemotherapy: a Gynecologic Oncology Group study." Int J Radiat Oncol Biol Phys **42**(5): 1015-1023.

Wolchok, J.D., A. Hoos, S. O'Day, *et al*. (2009). Guidelines for the evaluation of immune therapy activity in solid tumors: immune-related response criteria. *Clin Can Res*. 15:7412-7420.

1. Delgado G, Bundy B, Zaino R, Sevin BU, Creasman WT, Major F. Prospective surgical-pathological study of disease-free interval in patients with stage IB squamous cell carcinoma of the cervix: a Gynecologic Oncology Group study. *Gynecologic oncology.* 1990;38(3):352-357.
2. Rotman M, Sedlis A, Piedmonte MR, et al. A phase III randomized trial of postoperative pelvic irradiation in Stage IB cervical carcinoma with poor prognostic features: follow-up of a gynecologic oncology group study. *Int J Radiat Oncol Biol Phys.* 2006;65(1):169-176.
3. Macdonald OK, Chen J, Dodson M, Lee CM, Gaffney DK. Prognostic significance of histology and positive lymph node involvement following radical hysterectomy in carcinoma of the cervix. *American journal of clinical oncology.* 2009;32(4):411-416.
4. Rose PG, Bundy BN, Watkins EB, et al. Concurrent cisplatin-based radiotherapy and chemotherapy for locally advanced cervical cancer. *N Engl J Med.* 1999;340(15):1144-1153.
5. Keys HM, Bundy BN, Stehman FB, et al. Cisplatin, radiation, and adjuvant hysterectomy compared with radiation and adjuvant hysterectomy for bulky stage IB cervical carcinoma. *N Engl J Med.* 1999;340(15):1154-1161.
6. Varia MA, Bundy BN, Deppe G, et al. Cervical carcinoma metastatic to para-aortic nodes: extended field radiation therapy with concomitant 5-fluorouracil and cisplatin chemotherapy: a Gynecologic Oncology Group study. *International journal of radiation oncology, biology, physics.* 1998;42(5):1015-1023.
7. Clifford GM, Smith JS, Plummer M, Munoz N, Franceschi S. Human papillomavirus types in invasive cervical cancer worldwide: a meta-analysis. *Br J Cancer.* 2003;88(1):63-73.
8. Munoz N, Bosch FX, de Sanjose S, et al. Epidemiologic classification of human papillomavirus types associated with cervical cancer. *N Engl J Med.* 2003;348(6):518-527.
9. Cogliano V, Baan R, Straif K, et al. Carcinogenicity of human papillomaviruses. *Lancet Oncol.* 2005;6(4):204.
10. Topalian SL, Hodi FS, Brahmer JR, et al. Safety, activity, and immune correlates of anti-

PD-1 antibody in cancer. *N Engl J Med.* 2012;366(26):2443-2454.
11. Yang W, Song Y, Lu YL, Sun JZ, Wang HW. Increased expression of programmed death (PD)-1 and its ligand PD-L1 correlates with impaired cell-mediated immunity in high-risk human papillomavirus-related cervical intraepithelial neoplasia. *Immunology.* 2013;139(4):513-522.
12. Stone HB, Peters LJ, Milas L. Effect of host immune capability on radiocurability and subsequent transplantability of a murine fibrosarcoma. *Journal of the National Cancer Institute.* 1979;63(5):1229-1235.
13. Lee Y, Auh SL, Wang Y, et al. Therapeutic effects of ablative radiation on local tumor require CD8+ T cells: changing strategies for cancer treatment. *Blood.* 2009;114(3):589-595.
14. Sharabi AB, Lim M, DeWeese TL, Drake CG. Radiation and checkpoint blockade immunotherapy: radiosensitisation and potential mechanisms of synergy. *Lancet Oncol.* 2015;16(13):e498-509.
15. Hallahan D, Kuchibhotla J, Wyble C. Cell adhesion molecules mediate radiation-induced leukocyte adhesion to the vascular endothelium. *Cancer research.* 1996;56(22):5150-5155.
16. Twyman-Saint Victor C, Rech AJ, Maity A, et al. Radiation and dual checkpoint blockade activate non-redundant immune mechanisms in cancer. *Nature.* 2015;520(7547):373-377.
17. Young KH, Baird JR, Savage T, et al. Optimizing Timing of Immunotherapy Improves Control of Tumors by Hypofractionated Radiation Therapy. *PLoS One.* 2016;11(6):e0157164.
18. Liang JA, Chen SW, Hung YC, et al. Low-dose, prophylactic, extended-field, intensity-modulated radiotherapy plus concurrent weekly cisplatin for patients with stage IB2-IIIB cervical cancer, positive pelvic lymph nodes, and negative para-aortic lymph nodes. *Int J Gynecol Cancer.* 2014;24(5):901-907.
19. Vargo JA, Kim H, Choi S, et al. Extended field intensity modulated radiation therapy with concomitant boost for lymph node-positive cervical cancer: analysis of regional control and recurrence patterns in the positron emission tomography/computed tomography era. *Int J Radiat Oncol Biol Phys.* 2014;90(5):1091-1098.
20. Klopp A, Moughan J, Portelance L, et al. Hematologic toxicity on RTOG 0418: A phase II study of post-operative IMRT for gynecologic cancer. *Int J Radiat Oncol Biol Phys.* 2010:S121: 258.
21. Kidd E, Siegel B, Dehdashti F, al e. Clinical outcomes of definitive intensity-modulated radiation therapy with fluorodeoxyglucose-positron emission tomography simulation in patients with locally advanced cervical cancer. *Int J Radiat Oncol Biol Phys.* 2010;77:1085-1091.
22. Osborne EM, Klopp AH, Jhingran A, Meyer LA, Eifel PJ. Impact of treatment year on survival and adverse effects in patients with cervical cancer and paraortic lymph node metastases treated with definitive extended-field radiation therapy. *Pract Radiat Oncol.* 2017;7(3):e165-e173.
23. Hanks GE, Herring DF, Kramer S. Patterns of care outcome studies. Results of the national practice in cancer of the cervix. *Cancer.* 1983;51(5):959-967.
24. Potter R, Georg P, Dimopoulos JC, et al. Clinical outcome of protocol based image (MRI) guided adaptive brachytherapy combined with 3D conformal radiotherapy with or

without chemotherapy in patients with locally advanced cervical cancer. *Radiotherapy and oncology : journal of the European Society for Therapeutic Radiology and Oncology.* 2011;100(1):116-123.
25. Grover S, Harkenrider MM, Cho LP, et al. Image Guided Cervical Brachytherapy: 2014 Survey of the American Brachytherapy Society. *Int J Radiat Oncol Biol Phys.* 2016;94(3):598-604.
26. Davidson MT, Yuen J, D'Souza D, Batchelar DL. Image-guided cervix high-dose-rate brachytherapy treatment planning:
Does custom computed tomography planning for each insertion provide
better conformal avoidance of organs at risk? *Brachytherapy.* 2008;7:37-42.
27. Gao M, Albuquerque K, Chi A, Rusu I. 3D CT-based volumetric dose assessment of 2D plans using GEC-ESTRO guidelines for cervical cancer brachytherapy. *Brachytherapy.* 2010;9(1):55-60.
28. Haie-Meder C, Potter R, Van Limbergen E, et al. Recommendations from Gynaecological (GYN) GEC-ESTRO Working Group (I): concepts and terms in 3D image based 3D treatment planning in cervix cancer brachytherapy with emphasis on MRI assessment of GTV and CTV. *Radiotherapy and oncology : journal of the European Society for Therapeutic Radiology and Oncology.* 2005;74(3):235-245.
29. Potter R, Haie-Meder C, Limbergenc E. Recommendations from gynaecological (GYN) GEC ESTRO working
group (II): Concepts and terms in 3D image-based treatment planning
in cervix cancer brachytherapy—3D dose volume parameters and
aspects of 3D image-based anatomy, radiation physics, radiobiology. *Radiotherapy and Oncology.* 2006
78 67–77.
30. Pötter R, Kirisits C, Fidarova EF, et al. Present status and future of high-precision image guided adaptive brachytherapy for cervix carcinoma. *Acta Oncologica.* 2008;47(7):1325-1336.
31. van Dyk S, Byram D, Bernshaw D. Use of 3D imaging and awareness of GEC-ESTRO recommendations for cervix cancer brachytherapy throughout Australia and New Zealand. *J Med Imaging Radiat Oncol.* 2010;54(4):383-387.
32. Pavamani S, D'Souza DP, Portelance L, et al. Image-guided brachytherapy for cervical cancer: a Canadian Brachytherapy Group survey. *Brachytherapy.* 2011;10(5):345-351.
33. Mayadev J, Viswanathan A, Liu Y, et al. American Brachytherapy Task Group Report: A pooled analysis of clinical outcomes for high-dose-rate brachytherapy for cervical cancer. *Brachytherapy.* 2017;16(1):22-43.
34. Gyorki DE, Callahan M, Wolchok JD, Ariyan CE. The delicate balance of melanoma immunotherapy. *Clin Transl Immunology.* 2013;2(8):e5.
35. Atezolizumab, being a member of class of agents involved in the inhibition of “immune checkpoints,” may result in severe and possibly fatal immune-mediated adverse events probably due to T-cell activation and proliferation. Immune-mediated adverse reactions have been reported in patients receiving atezolizumab. Adverse events potentially related to atezolizumab may be manifestations of immune-mediated adverse events. In clinical trials, most immune-mediated adverse reactions were reversible and managed with interruptions of atezolizumab, administration of corticosteroids and supportive care.
36. Kunos C, Radivoyevitch T, Abdul-Karim FW, Faulhaber P. 18F-fluoro-2-deoxy-D-

glucose positron emission tomography standard uptake value ratio as an indicator of cervical cancer chemoradiation therapeutic response. *International journal of gynecological cancer : official journal of the International Gynecological Cancer Society*. 2011;21(6):1117-1123.

APPENDIX I – TRANSLATIONAL SCIENCE BIOSPECIMEN PROCEDURES

1. Obtaining a Bank ID for Translational Science Biospecimens

Only one Bank ID (# # # # - # # - G # # #) is assigned per patient. All translational science biospecimens and accompanying paperwork must be labeled with this coded patient number.

A Bank ID is automatically assigned once the Specimen Consent is completed and indicates that a patient has agreed to participate in the translational science component. If a patient has previously been assigned a Bank ID, please ensure the Bank ID appearing in Rave is the same as the previously assigned Bank ID.

Please contact User Support if you need assistance or have assigned more than one Bank ID to a patient (Email: support@nrgoncology.org; Phone: 716-845-7767).

2. Requesting Translational Science Biospecimen Kits

Three single chamber kits will be provided per patient for the collection and shipment of frozen plasma, buffy coat, and whole blood biospecimens. One kit will be provided for each of the following time points:

1. days -21 (regimen A only), 0, and 21;
2. days 42, and 63; and
3. day 140.

Sites can order kits online via the Kit Management system (https://ricapps.nationwidechildrens.org/KitManagement/). Each site may order two kit types per protocol per day (daily max = 6 kits).

Please contact the NRG BB-Columbus if you need assistance (Email: BPCBank@nationwidechildrens.org; Phone: 866-464-2262).

Be sure to plan ahead and allow time for kits to be shipped by ground transportation. Kits should arrive within 3-5 business days.

Note: Unused supplies and kits should be returned to the NRG BB-Columbus. A pre-paid shipping label for the return of unused supplies and kits may be obtained via the Kit Management system. Select "Empty Kit" for package contents when returning unused kits.

3. FFPE Tumor Biospecimens Shipped to the NRG BB-Columbus

Only one block may be submitted per tissue type. All FFPE tissue should be submitted with the corresponding pathology report. **If a pathology report if not available for the biopsy, a copy of the radiology report or operative report from the biopsy procedure <u>must</u> be sent to the NRG BB-Columbus, along with a completed copy of the Biopsy Pathology Verification (Appendix V).**

3.1 FFPE Biospecimen Requirement

3.1.1 Tumor Tissue Type

Formalin-fixed, paraffin embedded (FFPE) tissue should be the most representative of the required type:

<u>**Regimen A Only**</u>

- **D-21 FFPE primary (FP01) or metastatic (FM01) tumor biopsy** should be collected for after randomization, prior to the patient receiving any study treatment (day -21 ± 3 days).

<u>**Regimens A and B**</u>

- **D0 FFPE primary (FP02) or metastatic (FM02) tumor chemoradiation biopsy** should be collected at the start of chemoradiation (day 0 ± 3 days).
- **D28 FFPE primary (FP03) or metastatic (FM03) tumor brachytherapy biopsy** should be collected at the first brachytherapy (day 28 ± 7 days).

3.1.2 FFPE Type

Only <u>blocks</u> will be accepted. Please provide Appendix IV to your pathologist.

3.1.3 Labeling FFPE Biospecimens

A waterproof permanent marker or printed label should be used to label each translational science FFPE biospecimen with:

Bank ID (# # # # - # # - G # # #)
protocol number (NRG-GY017)
specimen code (see section 3.1.1 above)
collection date (mm/dd/yyyy)
surgical pathology accession number
block number

Note: If labeling slides, only label on the top, front portion of the slide. Do not place a label on the back of the slide or over the tissue. The label must fit on the slide and should not be wrapped around the slide or hang over the edge.

3.1.4 Completing Form TR for FFPE Biospecimens

The type of biospecimen (block) should be specified on Form TR.

The time the tissue was in formalin (i.e., fixation time) should be entered as Estimated Processing Time.

4. Plasma and Buffy Coat Biospecimens Shipped to the NRG BB-Columbus

4.1 Plasma and Buffy Coat Biospecimen Requirement

Plasma and buffy coat biospecimens should be collected at the following time points:

<u>**Regimen A Only**</u>

1. day -21, prior to atezolizumab (PB01 for plasma; LB01 for buffy coat);

<u>**Regimens A and B**</u>

2. day 0 ± 3 days (PB02 or plasma; LB02 for buffy coat);

3. day 21 ± 1 day (PB03 for plasma; LB03 for buffy coat);
4. day 42 ± 7 days, at start of brachytherapy (PB04 for plasma; LB04 for buffy coat);
5. day 63 ± 3 days (PB05 for plasma; LB05 for buffy coat); and
6. day 140 ± 7 days, at time of diagnostic PET/CT (PB06 for plasma; LB06 for buffy coat).

4.2 Processing Plasma and Buffy Coat Biospecimens

1. Label six cryovials (five for plasma and one for buffy coat) and a 15mL conical tube as described below. Use 2mL cryovials as plasma and buffy coat will be shipped to the NRG BB-Columbus.
2. Draw 7-10mL of blood into lavender/purple top (K2EDTA) tube(s).
3. Immediately after collection, gently invert the blood collection tube 5-10 times to mix the blood and K2EDTA.
4. Centrifuge the blood at 1000g for 15 minutes at 4ºC (preferred) or room temperature to separate the plasma (top, straw-colored layer) from the buffy coat (middle, white layer) and the red blood cells (bottom, red layer).
5. Transfer the plasma into a pre-labeled 15mL conical tube and gently mix.
6. Quickly, evenly dispense (aliquot) the plasma into five pre-labeled cryovials and cap the tubes securely. Place at least 0.25mL into each cryovial.
7. Transfer the buffy coat to the pre-labeled cryovial.
8. Immediately freeze the plasma and buffy coat in an upright position in a -70ºC to -80ºC freezer or by direct exposure with dry ice until ready to ship. If a -70ºC to -80ºC freezer is not available for storage, store and ship on dry ice within 24 hours of collection.

4.3 Labeling Plasma and Buffy Coat

A waterproof permanent marker or printed label must be used to label each translational science plasma and buffy coat biospecimen with:

Bank ID (# # # # - # # - G # # #)
protocol number (NRG-GY017)
specimen code (WB##; see section 4.1 above)
collection date (mm/dd/yyyy)

5. Whole Blood Shipped to the NRG BB-Columbus

5.1 Whole Blood Biospecimen Requirements

Whole blood should be collected at the following time points:

<u>Regimen A Only</u>

1. day -21, prior to atezolizumab (WB01);

<u>Regimens A and B</u>

2. day 0 ± 3 days (WB02);
3. day 21 ± 1 day (WB03);
4. day 42 ± 7 days, at start of brachytherapy (WB04);
5. day 63 ± 3 days (WB05); and
6. day 140 ± 7 days, at time of diagnostic PET/CT (WB06).

5.2 Processing Whole Blood Biospecimens

1. Label the lavender/purple top (EDTA) collection tube(s) as described below. Multiple tubes may be used to collect the required amount. **Do not use glass blood collection tubes.**
2. Draw 7-10mL of blood into the labeled lavender/purple top tube(s). A minimum of 3mL is needed for processing.
3. Immediately after collection, gently invert the tube 5-10 times to mix the blood and EDTA.
4. Immediately freeze the blood in an upright position in a -70°C to -80°C freezer or by direct exposure with dry ice until ready to ship. If a -70°C to -80°C freezer is not available for storage, store and ship on dry ice within 24 hours of collection.

5.3 Labeling Whole Blood

A waterproof permanent marker or printed label must be used to label each translational science whole blood biospecimen with:

Bank ID (# # # # - # # - G # # #)
protocol number (NRG-GY017)
specimen code (WB##; see section 5.1 above)
collection date (mm/dd/yyyy)

6. Submitting Form TR

A specimen transmittal form (i.e., Form TR) for each biospecimen will be available in the **Translational Research Folder in Rave**, once the Specimen Consent (located in the Baseline Folder) has been completed.

An electronically (i.e., Rave) completed copy of Form TR must accompany each biospecimen shipped to the NRG BB-Columbus. Handwritten forms will not be accepted.

Note: A copy does not need to be sent to the NRG BB-Columbus if biospecimens are not collected.

Form TR **must** be printed from the Translational Research Form screen in Rave using the **"PDF File" link at the top of the form**. Clicking this link will generate a single page PDF. Do not use the "Printable Version" or "View PDF" links at the bottom of the form or any other method to print the form, as these formats will not be accepted.

Note: Biospecimens will not be marked as received in Rave without receipt of a corresponding electronically completed Form TR. **Incomplete forms or those containing incorrect information will not be processed and may result in a data query.**

Retain a printout of the completed form for your records.

Please contact User Support if you need assistance (Email: support@nrgoncology.org; Phone: 716-845-7767).

7. Shipping Translational Science Biospecimens

7.1 General Information for Shipping Biospecimens to the NRG BB-Columbus

- Translational science biospecimens should not be shipped until after patient registration and Bank ID assignment.
- An electronically completed copy of Form TR must be included for each translational science biospecimen.
- All translational science biospecimens should be shipped to:

  NRG BB-Columbus / Protocol NRG-GY017
  Nationwide Children's Hospital
  700 Children's Dr, WA1340
  Columbus, OH 43205
  Phone: 614-722-2865
  FAX: 614-722-2897
  Email: BPCBank@nationwidechildrens.org

7.2 FFPE Tissue Shipped to the NRG BB-Columbus

FFPE tissue and a copy of the corresponding pathology report (or Biopsy Pathology Verification) should be shipped using your own container at your own expense to the NRG BB-Columbus (address in section 7.1).

Do not ship FFPE tissue for Saturday delivery.

7.3 Frozen Biospecimens Shipped to the NRG BB-Columbus

Frozen plasma, buffy coat, and whole blood should be shipped using the biospecimen kits provided to the NRG BB-Columbus (address in section 9.1).

Frozen biospecimens should be shipped **Monday through Thursday for Tuesday through Friday delivery**. Do not ship frozen biospecimens on Friday or the day before a holiday. Note: Saturday delivery is not available for frozen biospecimens.

Frozen biospecimens should be stored in an ultra-cold freezing/storage space (i.e., ultra-cold ≤-70°C freezer, liquid nitrogen, or direct exposure with dry ice) until the biospecimens can be shipped.

7.3.1 Shipping Frozen Translational Science Biospecimens in a Single Chamber Kit

1. Pre-fill the kit chamber about 1/3 full with dry ice.
2. Place the frozen biospecimens from each time point in a separate zip-lock bag.
3. Place the zip-lock bags in the biohazard envelope containing absorbent material. Do not put more than 25 cryovials in a single chamber kit. Put the secondary envelope into a Tyvek envelope. Expel as much air as possible before sealing both envelopes.
4. Place the Tyvek envelope containing the frozen biospecimens into the kit and fill the chamber to the top with dry ice.
5. Insert a copy of Form TR for each biospecimen.
6. Place the cover on top of the kit. Tape the outer box of the kit closed with filament or other durable sealing tape. Please do not tape the inner chamber.

7. Print a pre-paid FedEx air bill using the Kit Management link (https://ricapps.nationwidechildrens.org/KitManagement/). Attach the air bill.
8. Attach the dry ice label (UN1845) and the Exempt Human Specimen sticker.
9. Arrange for FedEx pick-up through your site's usual procedure or by calling 800-238-5355.

8. Banking Translational Science Biospecimens for Future Research

Biospecimens will remain in the NRG BB-Columbus and made available for approved research projects if the patient has provided permission for the use of her biospecimens for future health research.

Note: Testing of banked biospecimens will not occur until an amendment to this treatment protocol (or separate correlative science protocol) is reviewed and approved in accordance with National Clinical Trials Network (NCTN) policies.

The patient's biospecimen consent choices will be recorded on the signed informed consent document and electronically via the Specimen Consent form. At the time of biospecimen selection for project distribution, the most recent consent information will be used.

Sites can amend a patient's choices regarding the future use of her biospecimens at any time if the patient changes her mind.

If the patient revokes permission to use her biospecimens, the NRG BB-Columbus will destroy or return any remaining biospecimens. The patient's biospecimens will not be used for any further research; however, any biospecimens distributed for research prior to revoking consent cannot be returned or destroyed. In addition, the patient cannot be removed from any research that has been done with her biospecimens distributed prior to revoking consent.

Note: If return of biospecimens is requested, shipping will be at the site's expense.

APPENDIX II – PERFORMANCE STATUS CRITERIA

**ECOG Performance Status Scale**		**Karnofsky Performance Scale**	
Grade	Descriptions	Percent	Description
0	Normal activity. Fully active, able to carry on all pre-disease performance without restriction.	100	Normal, no complaints, no evidence of disease.
		90	Able to carry on normal activity; minor signs or symptoms of disease.
1	Symptoms, but ambulatory. Restricted in physically strenuous activity, but ambulatory and able to carry out work of a light or sedentary nature (*e.g.*, light housework, office work).	80	Normal activity with effort; some signs or symptoms of disease.
		70	Cares for self, unable to carry on normal activity or to do active work.
2	In bed <50% of the time. Ambulatory and capable of all self-care, but unable to carry out any work activities. Up and about more than 50% of waking hours.	60	Requires occasional assistance, but is able to care for most of his/her needs.
		50	Requires considerable assistance and frequent medical care.
3	In bed >50% of the time. Capable of only limited self-care, confined to bed or chair more than 50% of waking hours.	40	Disabled, requires special care and assistance.
		30	Severely disabled, hospitalization indicated. Death not imminent.
4	100% bedridden. Completely disabled. Cannot carry on any self-care. Totally confined to bed or chair.	20	Very sick, hospitalization indicated. Death not imminent.
		10	Moribund, fatal processes progressing rapidly.
5	Dead.	0	Dead.

APPENDIX III – RADIATION THERAPY CONTOURS

Example contours for the CTV primary, nodal volumes, PTV boost volumes.

CTV into 3 sub regions: CTVn (nodal), and CTVp1 and CTVp2 (primary). CTVp1 will consist of the gross tumor, cervix, and uterus as shown in Figure 1; CTVp2 consists of the parametria and superior third of the vagina (or half of the vagina, if the vagina is clinically involved), as shown in figure 2.

CTVn will include the PALN region to the vertebral level of L1/L2 interspace or 3 cm cranial to gross PALN disease, common, external, and internal iliac and presacral lymph nodes. It is acceptable to include the mesorectal nodes in CTVn. The upper border of the CTVn should not extend above the confluence of the common iliac arteries with the aorta (i.e., aortic bifurcation), and should begin no lower than superior border of L5. The pelvic

The CTVn will be obtained by ensuring an approximately 7 mm margin around the vessels, plus extension to include any adjacent visible lymph nodes, lymphoceles, or pertinent surgical clips. The presacral nodes should be contoured until the superior border of the S3 vertebral body is reached; below this point the nodal volume can be separated into two structures. The external iliac nodes should be contoured to the superior aspect of the femoral head. CTVn should be modified to exclude bone, muscle, and bowel. The CTV should not extend inferior to the ischial tuberosities.

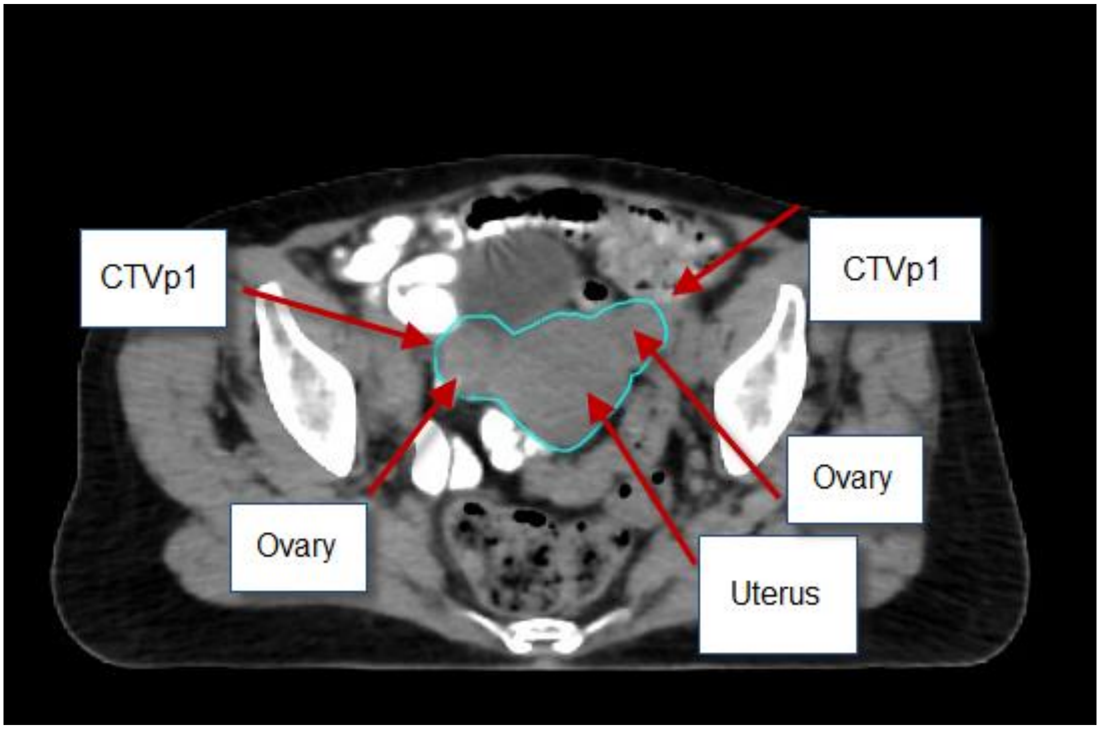

Figure 1: CTVp1

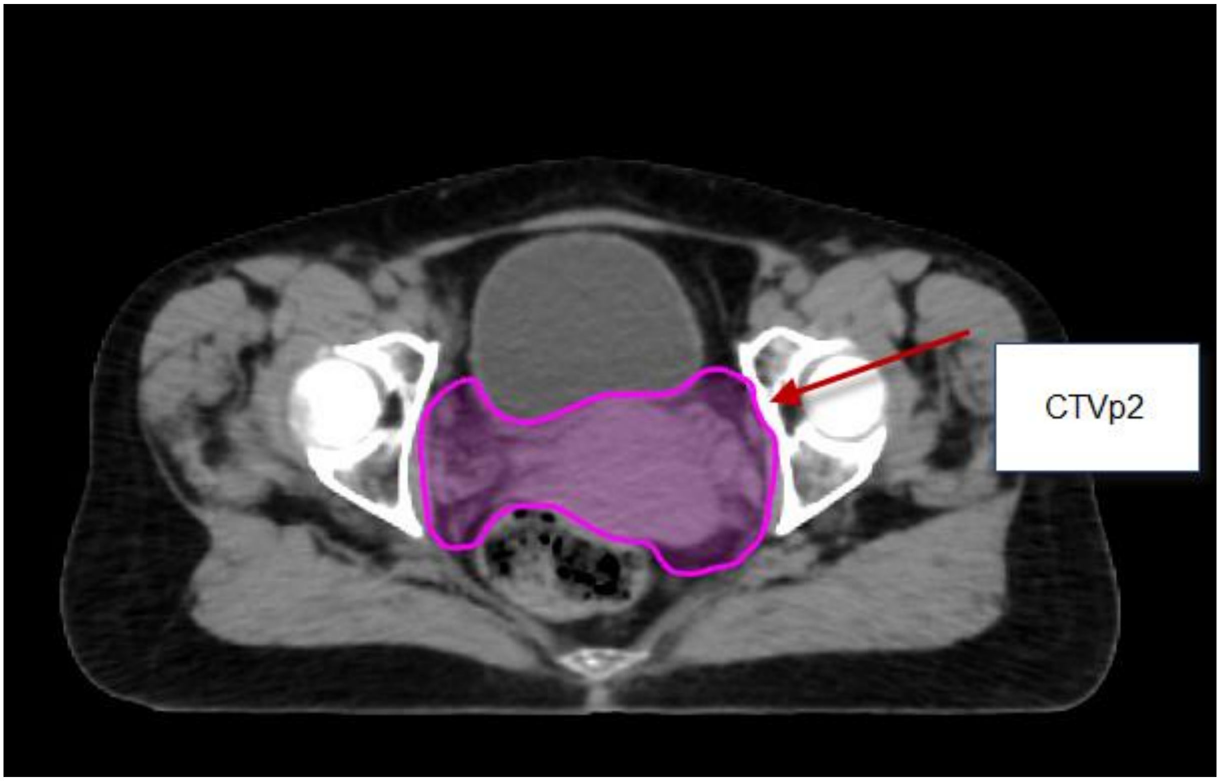

Figure 2: CTVp2

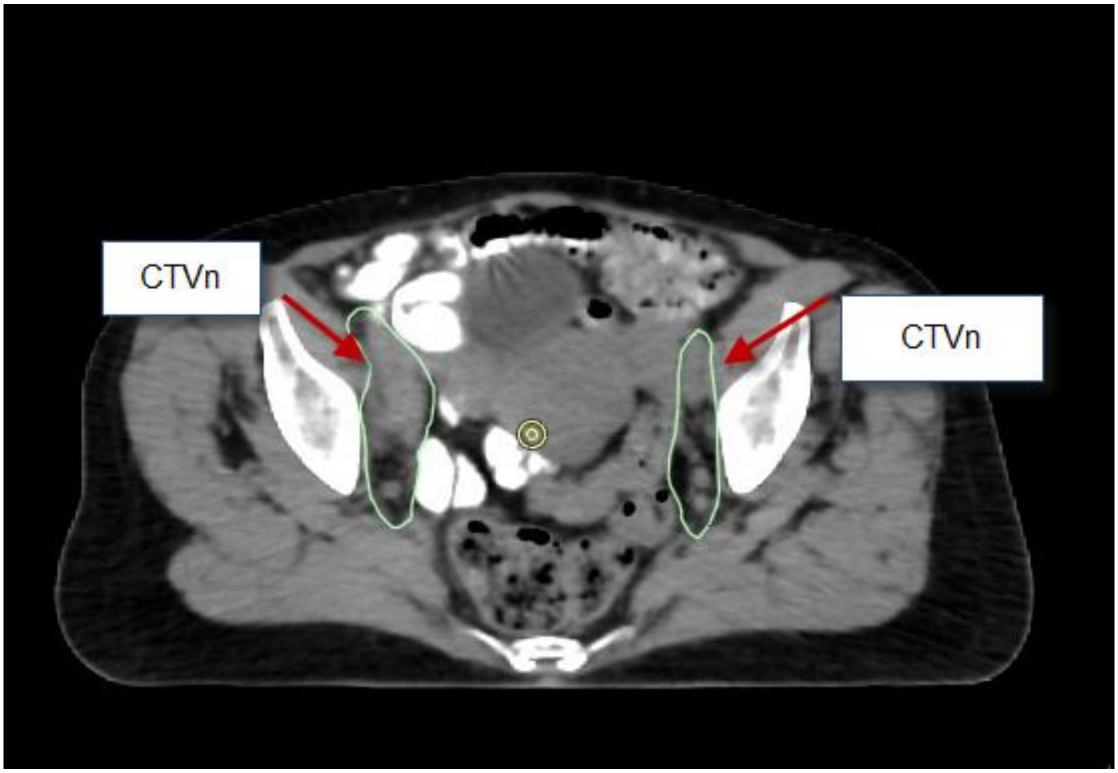

Figure 3: CTVn for pelvic LN locations

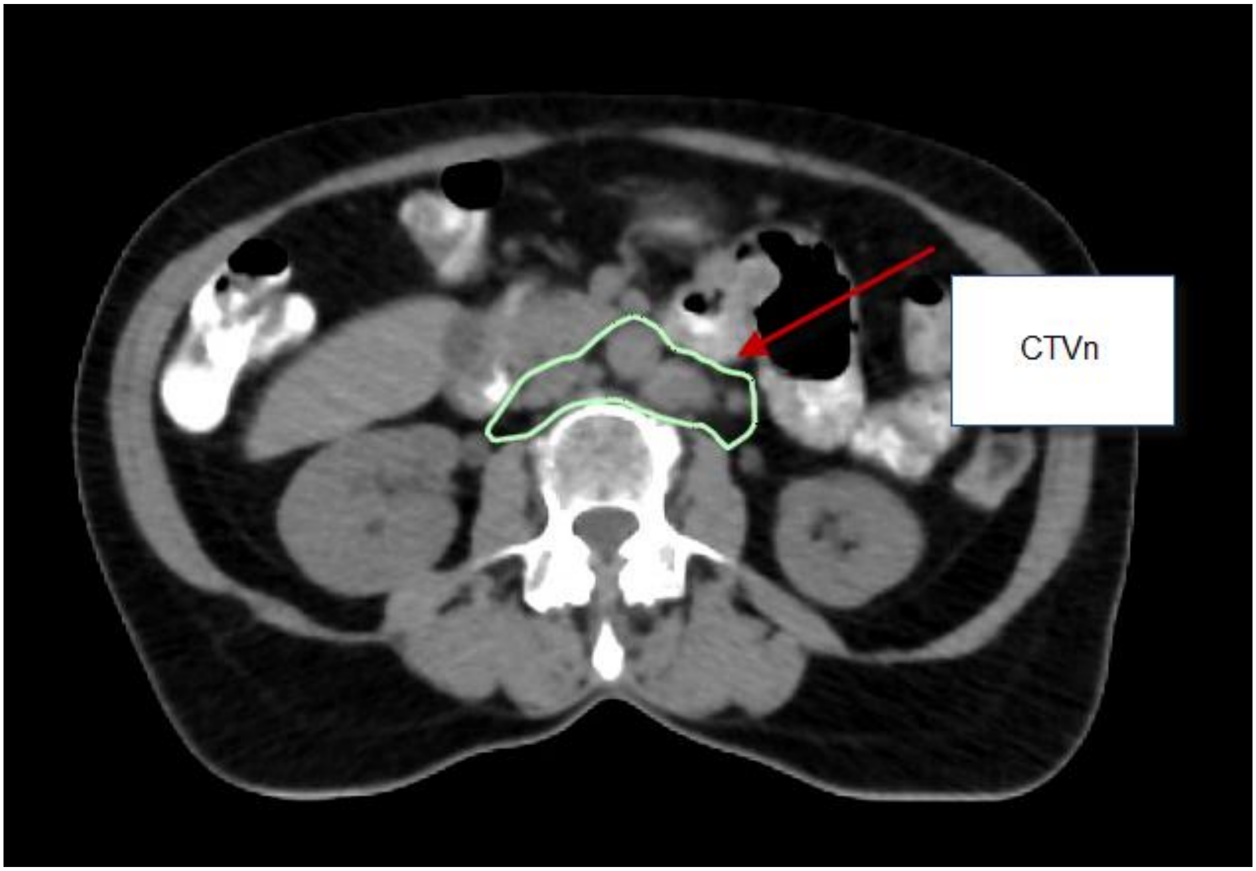

Figure 4: CTVn for PALN locations

CTV_5400: Gross pelvic lymph nodes (obturator LN, internal or external iliac LN) that will receive BT contribution. Parametrial boosts will be included in this volume. The PTV_5400 is a 7mm uniform expansion around CTV_5400. CTV_5800: Gross PALN or pelvic lymph nodes (common iliac high or low internal or external iliac LN) that will NOT receive BT contribution. PTV_5800 is a 7mm uniform expansion around CTV_5800.

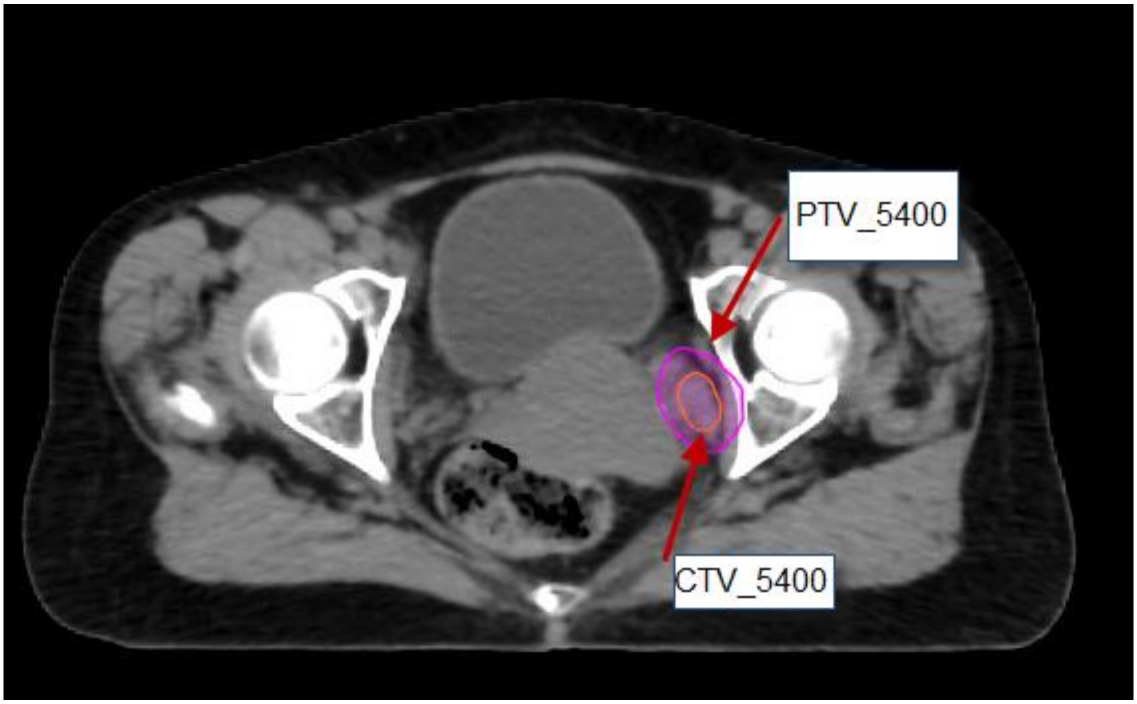

Figure 5: CTV_5400 and PTV_5400 for a pelvic LN boost

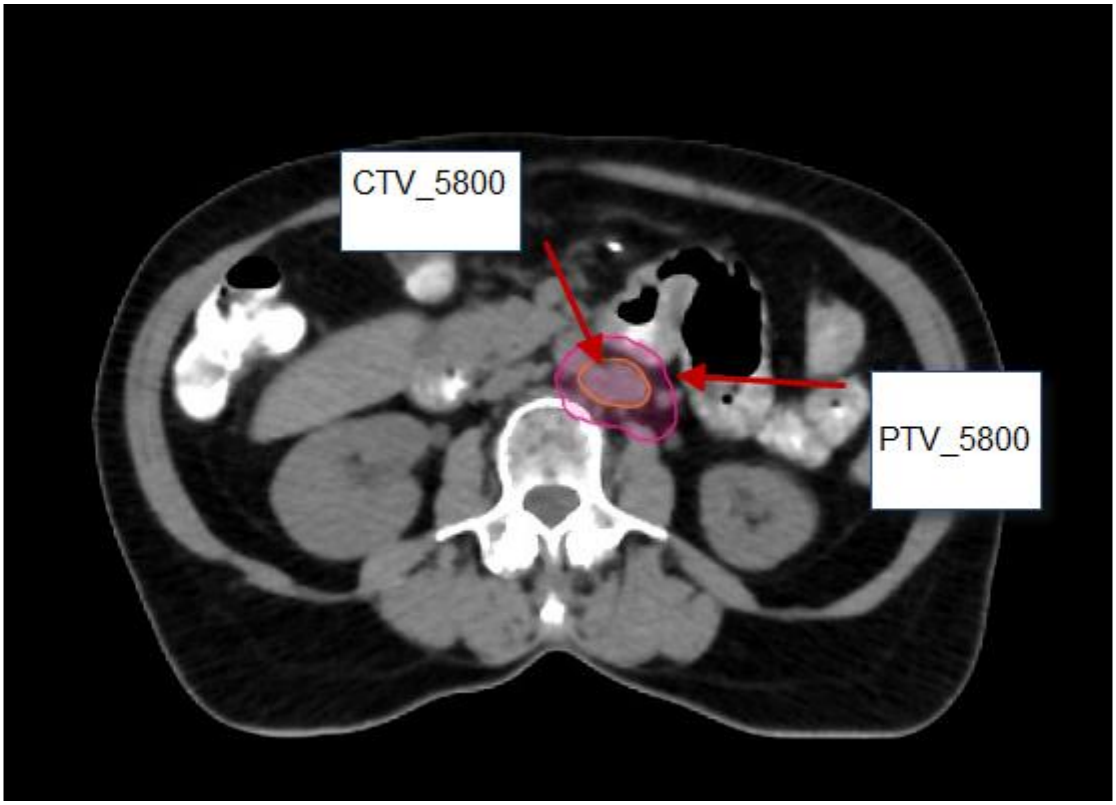

Figure 6: CTV_5800 and PTV_5800 for a PALN boost

APPENDIX IV – LETTER TO PATHOLOGISTS

Dear Pathologist,

Your site is a participant in **NRG GY017**, "Anti PD-L1 (Atezolizumab) as an Immune Primer and Concurrently with Extended Field Chemoradiotherapy for Node Positive Locally Advanced Cervical Cancer."

This study includes **integral and integrated biomarker testing.**

- The integral testing required to complete the **primary objective** of the study will use DNA isolated from formalin-fixed, paraffin-embedded (FFPE) tumor biopsies to examine T cell repertoires.
- The integrated testing required to complete a **secondary objective** will use FFPE tumor biopsies to stain for PD-L1 at a sponsor-designated laboratory. The PD-L1 testing requirements mandate that slides used for testing must be *fresh cut*.

Given the biospecimen requirements for this biomarker testing, **NRG GY017 requires all sites submit FFPE blocks only (i.e., unstained slides will not be accepted).** Blocks may be submitted on a permanent or temporary basis.

If submitted on a temporary basis, blocks will be returned after the completion of the integral and integrated biomarker testing. The NRG Biospecimen Bank (BB)-Columbus will section blocks every six months. At the time of sectioning, the NRG BB-Columbus will fresh cut ten consecutive unstained sections (5µm each) for shipment to the designated laboratories.

If return of the block is requested, the NRG BB-Columbus will contact your institution for a Fed Ex Account number and shipping address *after* completion of the integral and integrated biomarker testing.

If you should have any questions, please do not hesitate to contact Drs. Jyoti Mayadev (PI) and Heather Lankes (Translational Research Scientist).

We thank you in advance for your participation in this trial and your commitment to the successful completion of this study's objectives.

Sincerely,

Jyoti Mayadev, MD
Heather A Lankes, PhD, MPH

APPENDIX V - BIOPSY PATHOLOGY VERIFICATION

A copy of the corresponding pathology report must be shipped with all tissue biospecimens sent to the NRG BB-Columbus.

If a pathology report is not available for the biopsy, a copy of the radiology report or operative report from the biopsy procedure must be sent to the NRG BB-Columbus. A completed copy of this appendix (i.e., Biopsy Pathology Verification) must also be submitted to the NRG BB-Columbus.

Note: If this information is not provided with the biopsy biospecimen, it will not be accepted by the NRG BB-Columbus.

Please have the pathologist responsible for signing out this patient's case complete the following:

Bank ID: __ __ __ __ - __ __ - G __ __ __

Study ID: __ __ __ __ __ - G Y 0 1 7 - __ __ __ __ __

Date of Procedure (mm/dd/yyyy): ____

Tissue Type (circle one): Recurrent Primary Recurrent Metastatic

Site Tissue Taken From: ____

Diagnosis: ____

Recurrent disease documented by: ____

I agree that this tissue may be released for research purposes only and that the release of this tissue will not have any impact on the patient's care.

___________________________________ ______________________
Pathologist's Signature Date

____
Pathologist's Printed Name

APPENDIX VI – NCI/DCTD COLLABORATIVE AGREEMENTS LANGUAGE

Collaborative Agreements Language

Protocols that involve agent(s) covered by a collaborative agreement with a biotech/pharma company(ies) must incorporate the NCI/ DCTD Collaborative Agreement Language shown below.

The agent(s) supplied by CTEP, DCTD, NCI used in this protocol is/are provided to the NCI under a Collaborative Agreement (CRADA, CTA, CSA) between the Pharmaceutical Company(ies) (hereinafter referred to as "Collaborator(s)") and the NCI Division of Cancer Treatment and Diagnosis. Therefore, the following obligations/guidelines, in addition to the provisions in the "Intellectual Property Option to Collaborator" (http://ctep.cancer.gov/industryCollaborations2/intellectual_property.htm) contained within the terms of award, apply to the use of the Agent(s) in this study:

1. Agent(s) may not be used for any purpose outside the scope of this protocol, nor can Agent(s) be transferred or licensed to any party not participating in the clinical study. Collaborator(s) data for Agent(s) are confidential and proprietary to Collaborator(s) and shall be maintained as such by the investigators. The protocol documents for studies utilizing Agents contain confidential information and should not be shared or distributed without the permission of the NCI. If a copy of this protocol is requested by a patient or patient's family member participating on the study, the individual should sign a confidentiality agreement. A suitable model agreement can be downloaded from: http://ctep.cancer.gov.

2. For a clinical protocol where there is an investigational Agent used in combination with (an)other Agent(s), each the subject of different Collaborative Agreements, the access to and use of data by each Collaborator shall be as follows (data pertaining to such combination use shall hereinafter be referred to as "Multi-Party Data"):

a. NCI will provide all Collaborators with prior written notice regarding the existence and nature of any agreements governing their collaboration with NCI, the design of the proposed combination protocol, and the existence of any obligations that would tend to restrict NCI's participation in the proposed combination protocol.

b. Each Collaborator shall agree to permit use of the Multi-Party Data from the clinical trial by any other Collaborator solely to the extent necessary to allow said other Collaborator to develop, obtain regulatory approval or commercialize its own Agent.

c. Any Collaborator having the right to use the Multi-Party Data from these trials must agree in writing prior to the commencement of the trials that it will use the Multi-Party Data solely for development, regulatory approval, and commercialization of its own Agent.

3. Clinical Trial Data and Results and Raw Data developed under a Collaborative Agreement will be made available to Collaborator(s), the NCI, and the FDA, as appropriate and unless

additional disclosure is required by law or court order as described in the IP Option to Collaborator (http://ctep.cancer.gov/industryCollaborations2/intellectual_property.htm). Additionally, all Clinical Data and Results and Raw Data will be collected, used and disclosed consistent with all applicable federal statutes and regulations for the protection of human subjects, including, if applicable, the *Standards for Privacy of Individually Identifiable Health Information* set forth in 45 C.F.R. Part 164.

4. When a Collaborator wishes to initiate a data request, the request should first be sent to the NCI, who will then notify the appropriate investigators (Group Chair for Cooperative Group studies, or PI for other studies) of Collaborator's wish to contact them.

5. Any data provided to Collaborator(s) for Phase 3 studies must be in accordance with the guidelines and policies of the responsible Data Monitoring Committee (DMC), if there is a DMC for this clinical trial.

6. Any manuscripts reporting the results of this clinical trial must be provided to CTEP by the Group office for Cooperative Group studies or by the principal investigator for non-Cooperative Group studies for immediate delivery to Collaborator(s) for advisory review and comment prior to submission for publication. Collaborator(s) will have 30 days from the date of receipt for review. Collaborator shall have the right to request that publication be delayed for up to an additional 30 days in order to ensure that Collaborator's confidential and proprietary data, in addition to Collaborator(s)'s intellectual property rights, are protected. Copies of abstracts must be provided to CTEP for forwarding to Collaborator(s) for courtesy review as soon as possible and preferably at least three (3) days prior to submission, but in any case, prior to presentation at the meeting or publication in the proceedings. Press releases and other media presentations must also be forwarded to CTEP prior to release. Copies of any manuscript, abstract and/or press release/ media presentation should be sent to:

Email: ncicteppubs@mail.nih.gov

The Regulatory Affairs Branch will then distribute them to Collaborator(s). No publication, manuscript or other form of public disclosure shall contain any of Collaborator's confidential/ proprietary information.
